# Integrated Analysis of Transcriptome mRNA and miRNA Profiles Reveals Self-Protective Mechanism of Bovine MECs Induced by LPS

**DOI:** 10.3389/fvets.2022.890043

**Published:** 2022-06-23

**Authors:** Ling Chen, Xiaolin Liu, Zhixiong Li, Jian Wang, Rongfu Tian, Huilin Zhang

**Affiliations:** ^1^School of Modern Agriculture and Biotechnology, Ankang University, Ankang, China; ^2^College of Animal Science and Technology, Northwest A&F University, Xianyang, China; ^3^College of Life Science and Technology, Southwest University for Nationalities, Chengdu, China

**Keywords:** LPS, bMEC, mastitis, mRNA profile, miRNA profile, self-protection

## Abstract

Many studies have investigated the molecular crosstalk between mastitis-pathogens and cows by either miRNA or mRNA profiles. Here, we employed both miRNA and mRNA profiles to understand the mechanisms of the response of bovine mammary epithelial cells (bMECs) to lipopolysaccharide (LPS) by RNA-Seq. The total expression level of miRNAs increased while mRNAs reduced after LPS treatment. About 41 differentially expressed mRNAs and 45 differentially expressed miRNAs involved in inflammation were screened out. We found the NFκB-dependent chemokine, *CXCL1, CXCL3, CXCL6, IL8*, and *CX3CL1* to be strongly induced. The anti-apoptosis was active because *BCL2A1* and *BIRC3* significantly increased with a higher expression. The effects of anti-microbe and inflammation were weakly activated because *TNF, IL1, CCL20, CFB, S100A, MMP9*, and *NOS2A* significantly increased but with a low expression, *IL6* and β*-defensin* decreased. These activities were supervised by the *NFKBIA* to avoid excessive damage to bMECs. The *bta-let-7a-5p, bta-miR-30a-5p, bta-miR-125b*, and *bta-miR-100* were essential to regulate infection process in bMECs after LPS induction. Moreover, the lactation potential of bMECs was undermined due to significantly downregulated *SOSTDC1, WNT7B, MSX1*, and *bta-miR-2425-5p*. In summary, bMECs may not be good at going head-to-head with the pathogens; they seem to be mainly charged with sending out signals for help and anti-apoptosis for maintaining lives after LPS induction.

## Introduction

Mastitis is the most frequent disease afflicting dairy cows and it has well-known detrimental effects on animal wellbeing and dairy farm profitability, including decreased milk production and quality, increased discarded milk, cow mortality, and cull rate. Metagenomic studies indicate that diverse bacterial groups are found in the mammary secretions of clinically healthy quarters, whereas the microbiota of mastitic quarters, or those with a history of mastitis, are considerably less diverse (1), which is due to the ability of the majority of mastitis pathogens to survive and proliferate within the intramammary ecosystem at a relatively fast pace (2). For the host, mastitis outcomes depend not only on the virulence of the bacteria (3) but also on the strength and timely development of the defense mechanism of the host (4). Thus, genetic improvement of the host is considered to be an interesting option for diminishing the frequency of mastitis.

The immune response associated with mastitis is a very complex biological process, involving not only resident and recruited immune cells, but also mammary epithelial cells (5, 6). The mammary epithelium is not just a passive physical barrier, but a critical regulator within the establishment of the immune response toward mastitis, *via* major activation of the transcription factor, NFκB, and the intensity of the response of the mammary epithelial cells (MECs) depends upon the invading pathogen (7, 8). *Escherichia coli* and *Staphylococcus aureus* are well-studied major pathogens involved in mastitis and often cause different immune responses in bMECs (8). It has previously been reported that the induction of tumor necrosis factor-alpha (TNFα), interleukin 1 (IL1), and activated NF-κB in bMECs and udder stimulated with *E. coli* was stronger than that stimulated with *S. aureus* (8, 9). Lipopolysaccharide (LPS) is a glycolipid derived from the outer membrane of gram-negative bacteria (including *E. coli*) that is capable of eliciting a robust pro-inflammatory response in a wide variety of mammalian cells and is dose-dependent (10).

Following infection, an immune response is initiated by cell surface receptors, particularly toll-like receptors (TLRs). Multiple TLRs respond to different pathogens and trigger downstream signaling, which leads to the production of cytokines and chemokines (11). For example, TLR4 recognizes the LPS of gram-negative microbes, while TLR2, which forms a heterodimer with TLR1 or TLR6, responds to peptidoglycan (PGN) and lipoteichoic acid (LTA) of multiple gram-positive microbes. TLR4 requires the help of LPS binding protein (LBP), cluster of differentiation 14 (CD14), and myeloid differentiation protein 2 (MD2) (12) to activate downstream signaling, which can occur *via* an adaptor protein, myeloid differentiation factor 88 (MyD88)-dependent and -independent cascades (13) leading to (1) a rapid activation of nuclear factor, NF-κB and (2) interferon regulatory factor-3 or a more moderate, delayed NF-κB activation, respectively (14). Two adaptor proteins, MyD88 and TRIF, are the key regulators of the TLR signal transduction pathway.

Characterization of the transcriptomic response to infection in different tissues is an effective approach to understanding the response mechanism of dairy mastitis. In the past decades, a large number of mastitis-related mRNAs and miRNAs have been identified by the Transcriptome-Seq in infected bMECs (9, 15–17), udder tissues (18–20), milk whey (21), milk-derived exosomes (22), whole blood (23–25) and blood-isolated monocytes (26) in the context of *S. aureus, Streptococcus uberis, Streptococcus agalactiae*, and *E. coli*. MECs have a strong capacity to mount differing innate immune responses to gram-positive and gram-negative bacterial challenges in their own way. The initial response of these cells is probably crucial to delay invading bacteria while secreting large amounts of cytokines and chemokines for leukocyte recruitment and activation (27). However, they can also express factors contributing directly to fighting off pathogens, including the bactericidal defensins (LAP and BNBD5), some complement factors, and acute-phase proteins (5, 7).

The transcriptome is dynamic and responsive. Numerous biological and physical entities, as well as temporal and environmental factors, can alter the transcriptome signature. Capturing subtle transcriptomic changes can provide insight into molecular mechanisms underlying the biological processes. However, most of the data mentioned above, including mRNome and miRNome were acquired separately from different labs and conditions, such as different microarray platforms (28), non-standardized analysis tools, and statistical cutoffs (29); large biological variations in out-bred populations commonly used in challenge experiments, small sample size, and sampling bias in the mammary gland, make a direct comparison of transcriptome results among the results published by various labs difficult. Therefore, under identical experimental conditions, combining mRNome and miRNome analysis with bioinformatics is more appropriate to avoid these mutation conditions and generate a more complete and accurate gene network list (20).

Hence, the current investigation has examined the transcriptomic mRNAs and miRNAs profiles of MAC-T cells, an SV40-immortalized bovine mammary epithelial cell line to discover its innate immune responses, and lactation activity after LPS-challenge. The discovery and holistic analysis of an extensive systemic reaction in the mammary epithelial cells significantly expand the knowledge of host–pathogen interactions in mastitis which may be relevant for the development of novel therapies and genetic selection toward mastitis resistance.

## Materials and Methods

### Cell Culture and LPS Challenge

MAC-T cells were purchased from ATCC (Manassas, USA). The cells were seeded at a concentration of 1.5 × 10^5^ cells in 10 cm^2^ cell culture plate (Nunc, Denmark) and incubated in a growth medium at 37°C with 5% of CO_2_. The growth medium was RPMI 1640 (Hyclone, Logan, USA) containing 10% of fetal bovine serum (FBS; Sijiqing, Tianhang Biotechnology Co., Ltd, Hangzhou, China) and 1% of penicillin-streptomycin solution (Hyclone, USA). Adherent cells were released by 0.25% of trypsin (Hyclone, USA).

Crude LPS purified by phenol extraction from *E. coli* O55:B5 (L2880; Sigma, St. Louis, USA) was the induction agent, which is commonly used for the experimental modeling of the acute-phase response. Before stimulation, the growth medium was replaced by another medium containing RPMI 1640, 5% of FBS only for 24 h. Cells at 70% confluence were treated with a stimulation medium consisting of RPMI 1640, 5% FBS, and 20 μg/ml of crude LPS (O55:B5). After incubation for 0, 4, 8, and 12 h in triplicates, cell culture supernatant was removed, and cells were washed with 1 × PBS. Finally, cells were collected and suspended in 1 × PBS containing 0.5 M of EDTA for RNA extraction.

### Bovine MEC RNA Isolation

Total RNA of bMECs was extracted using the Trizol reagent (Invitrogen, Carlsbad, CA, USA) following the manufacturer's procedure. The total RNA quantity and purity of bMECs were analyzed by Bioanalyzer 2100 and RNA 6000 Nano LabChip Kit (Agilent, CA, USA) with RIN number > 7.0. Each RNA sample was diluted with RNase-free ddH2O and was used either immediately or frozen at −80°C for future use. Total RNA of MECs at each time-point in triplicates was used for sequencing and QT-PCR experiments.

### The mRNA Library Construction

Total RNA of MECs at each time-point in triplicates was mixed as 0 (control), 4, 8, and 12 h samples, respectively, for sequencing. Approximately, 10 μg of total RNA representing a specific LPS-challenged bMECs sample was subjected to isolate Poly (A) mRNA with poly-T oligo-attached magnetic beads (Invitrogen). Following purification, the mRNA is fragmented into small pieces using divalent cations under elevated temperatures. Then the cleaved RNA fragments were reverse-transcribed to create the final cDNA library in accordance with the protocol for the mRNA-Seq sample preparation kit (Illumina, San Diego, USA), and the average insert size of the paired-end libraries was 300 bp (±50 bp). Then, we performed the paired-end sequencing on an Illumina Hiseq2000/2500 at the LC Sciences (Houston, USA) following the vendor's recommended protocol.

### Sequencing and Primary Analysis

A total of 35, 36, 91, and 85 million paired-end raw data reads were generated, respectively (Table 1). Prior to assembly, the low-quality reads were removed. After that, a total of 4.38, 4.42, 11.23, and 10.58 G bp of cleaned, paired-end reads representing 0, 4, 8, and 12 h LPS-challenged bMECs samples were produced. The clean/valid sequence data have been submitted to the NCBI short-read archive (SRA) with accession numbers, SRX6610783, SRX6610784, SRX6610785, and SRX6610786 for mRNA-Seq.

**Table 1:** The results of transcriptome sequencing data.

**Sample**	**Raw data**	**Valid data**	**Q20%**	**Total transcripts**
	**Reads**	**Reads**		
0 h	35,336,302	35,058,102	99.38	49,092
4 h	35,636,074	35,366,388	99.47	49,192
8 h	90,591,310	89,805,142	98.56	52,102
12 h	85,460,656	84,636,532	98.69	52,019

*Q20% represents the proportion of raw data with nucleotide q-quality > 20*.

### RNA-Seq Reads Mapping, Transcript Abundance Estimation, and Differential Expression Testing

We aligned reads of four bMEC samples to the UCSC (http://genome.ucsc.edu/) bovine reference genome using Tophat package. The aligned read files were processed by Cufflinks (https://github.com/santosjorge/cufflinks), which uses the normalized RNA-seq fragment counts to measure the relative abundances of the transcripts. The unit of measurement is fragment per kilobase of exon per million (FPKM) fragments mapped. Secondly, Cuffmerge was used to co-merge all transcripts of four bMEC samples to generate unique transcripts. The downloaded UCSC GTF file was passed to Cuffdiff along with the original alignment (SAM) files produced by Tophat. Cuffdiff re-estimates the abundance of the transcripts listed in the GTF file using alignments from the SAM file and concurrently tests for differential expression. Only the comparisons with a “q-value” < 0.01 and status marked as “OK” in the Cuffdiff output were regarded as showing differential expression (provided by LC Sciences, Houston, USA).

The differential genes are selected based on the following criteria: (1) if the change in gene expression levels between two samples is greater than or equal to two-fold (|log_2_(fold change) |≥1); (2) if *p* ≤ 0.05 in a Fisher's exact test based on the edgeR software; (3) if the transcripts level (FPKM) at each sample is ≥10.

### Small RNA Library Construction, Sequencing, and Primary Analysis

Approximately, 1 ug of total RNA in each sample was used to prepare a small RNA library according to the protocol of TruSeq Small RNA Sample Prep Kits (Illumina, San Diego, USA). And then we performed the single-end sequencing (36 bp) on an Illumina Hiseq2500 at the LC-BIO (Hangzhou, China) following the recommended protocol.

### Small RNA Data Processing

Data processing followed the procedures as provided by LC Sciences Service. Briefly, the raw reads were subjected to the Illumina pipeline filter, and then the dataset was further processed with an in-house program, ACGT101-miR (LC Sciences, Houston, USA) to remove adapter dimers, junk, low complexity, common RNA families (rRNA, tRNA, snRNA, and snoRNA), and repeats. The clean/valid sequence data have been submitted to the NCBI short read archive (SRA), and the accession numbers are SRX6610779, SRX6610780, SRX6610781, and SRX6610782 for miRNA-seq.

Subsequently, unique sequences with lengths in approximately 18–26 nucleotides were mapped to specific species precursors in miRbase 22.0 by BLAST search to identify known miRNAs and novel 3p- and 5p- derived miRNAs. Length variation at both 3′ and 5′ ends and one mismatch inside of the sequence were allowed in the alignment. The unique sequences mapping to specific species matured miRNAs in hairpin arms were identified as known miRNAs. The unique sequences mapping to the other arm of the known specific species precursor hairpin opposite to the annotated mature miRNA-containing arm were considered to be novel 5p- or 3p- derived miRNA candidates.

The remaining sequences were mapped to other precursors of selected species (with the exclusion of specific species) in miRBase 22.0 by BLAST search, and the mapped pre-miRNAs were further BLASTed against the specific species genomes to determine their genomic locations. We defined the above two as known miRNAs. The unmapped sequences were BLASTed against the specific genomes and were defined as new miRNAs (Provided by LC Sciences, Houston, Texas, USA).

### Analysis of Differential Expressed miRNAs and Prediction of the Targeted Genes

The miRNA differential expression based on normalized deep-sequencing counts was analyzed by selectively using Fisher's exact test based on the edgeR software. The significance threshold was set to be 0.05 (*p* ≤ 0.05) in each test. The miRNAs were regarded as differentially expressed when |log_2_(fold change between samples)|≥1, and the miRNA counts at each sample were ≥10. To predict the targeted genes of differentially expressed miRNAs, two computational target prediction algorithms (TargetScan 7.1 and miRanda 3.3a) were used to identify the miRNA binding sites. Finally, the data predicted by both algorithms were combined and the overlaps were calculated.

### Bioinformatics Analysis of Differentially Expressed mRNA/miRNA

Gene ontology (GO) annotation and Kyoto Encyclopedia of Genes and Genomes (KEGG) analysis were, respectively, performed with DAVID 6.8 (https://david.ncifcrf.gov/) and KOBAS 3.0 (http://kobas.cbi.pku.edu.cn/index.php). Then, the GO terms and KEGG pathways with adjusted *p* ≤ 0.05 were significantly enriched in differentially expressed mRNA (DEGs) or differentially expressed miRNAs (DEMs). The results of this study were visualized with the ggplot2 package of R software (version 4.1.1) except for the network diagram, which was depicted by Cytoscape software (version 3.7.0; https://cytoscape.org/), including the interactions between DEGs and DEMs, and the relationship of DEGs with GO/KEGG terms.

### The cDNA Synthesis and Real-Time Quantitative PCR

The cDNAs for 12 bMECs samples (0, 4, 8, and 12 h in triplicates) were performed using the FastQuant RT Kit (with gDNase) (TIANGEN, Biotech Co., Ltd, Beijing, China) according to the manufacturer's instructions. Primers for mRNAs were designed on the basis of the published mRNA sequences. Primers for microRNAs were designed according to sequencing results. All primers were synthesized by Genscript Biotech Corporation (Nanjing, China) (Supplementary Table 1). Stem-loop real-time quantitative PCR (qPCR) was used to analyze miRNAs. The details of the process for cDNA synthesis were performed in two steps: (1) gDNA Eraser: the gDNA Eraser reaction mixture (10 μL of total volume contained 500 ng of total RNA, 2.0 μL of 5 × gDNA Buffer, 6.0–8.0 μL of RNase-free H_2_O) was mixed with the total RNA extracted from the bMEC. The mixture was incubated at 42°C for 3 min to promote the degradation of gDNA; (2) First-strand cDNA synthesis: 10 μL of total reverse transcription reaction mixture (2.0 μL of 50.0 pmol FQ-RT Primer (Oligo dT and R6 mix for mRNA, stem-loop primer for miRNA) mix, 2.0 μL of 10xFast RT Buffer, 1.0 μL of RT Enzyme Mix, and 5.0 μL of RNase-free ddH_2_O) was added to 10 μL of gDNA Eraser reaction mixture. Then, the mixture was incubated at 42°C for 15 min, 95°C for 3 min, and then immediately placed on ice. The cDNA was tested by PCR amplification with β-actin primer, then frozen at −20°C for further use.

The qPCR was performed in three independent experiments. β-actin was used as the internal control for qPCR detection of miRNAs and mRNAs. The qPCR was performed in a CFX96 Touch Real-Time PCR Instrument (BIO-RAD, USA) with the UltrSYBR Mixture kit (ComWin, Biotech Co., Ltd., Beijing, China) in a total volume of 25 μL (12.5 μL of 2 × UltraSYBR Mixture, 1.0 μL of PCR Forward Primer (10 μM), 1.0 μL of PCR Reverse Primer (10 μM), 100 ng of cDNA, and Rnase-free H_2_O to a final volume of 25 μL). The reaction mixtures were incubated at 95°C for 10 min, followed by 39 cycles of 95°C for 10 s, 60°C for 30 s, and 72°C for 32 s. The primers used in this assay are listed in Supplementary Table 1. At the end of each run, a dissociation melting curve of the product was determined. All melting curves showed a single peak and were consistent with the presence of a single amplicon. All amplifications were run in triplicate, and any ambiguous curves were excluded.

### Data Analysis for qPCR

The relative expression levels of mRNAs and miRNAs were calculated using 2^−ΔΔct(Samples−0h)^ = 2^−[Δct(Samples)−Δct(0h)]^. Gene expression levels were evaluated in three replicates. All gene expression data were expressed as mean ± SD. GraphPad Prism 6.0 software (GraphPad Software, Inc., La Jolla, CA, USA) was used for statistical analysis and the Student's *t*-test was used to test the significance of differences. If the corrected *P*-value was <0.05, the difference was deemed significant; if the corrected *P*-value was <0.01, then the difference was deemed extremely significant.

## Results

### Overview of Transcriptome Sequencing Data in LPS-Induced Bovine MEC

**Figure 1:**
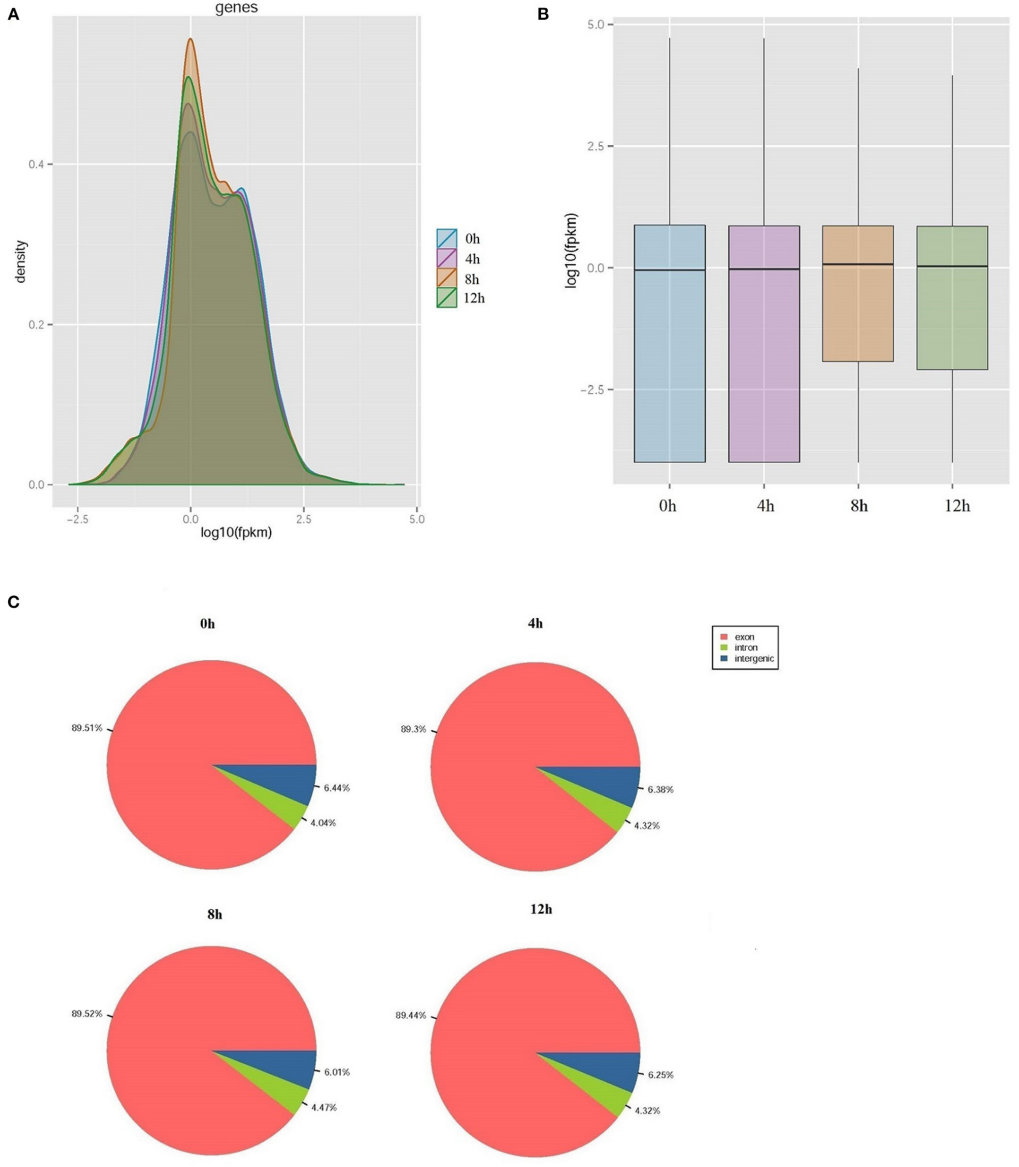
Overview of transcriptome sequencing data. **(A)** The relative expression density of transcripts; **(B)** The relative expression level of transcripts; **(C)** The genome distribution of clean reads in lipopolysaccharide (LPS)-induced bovine mammary epithelial cells at 0, 4, 8, and 12 h, respectively.

The proportion of raw data with nucleotide q-quality > 20 was more than 98% at four-time points. A total of 35,058,102; 35,366,388; 89,805,142, and 84,636,532 corresponding clean reads were produced from LPS-induced bMECs at 0, 4, 8, and 12 h, respectively (Table 1). The number of transcripts in 8 and 12 h group were far more than that of the 0 and 4 h group, and the expression level was more focused (Figures 1A,B). Correspondingly, 49,092; 49,192; 52,102, and 52,019 transcripts were obtained in LPS-induced bMECs at 0, 4, 8, and 12 h, respectively. For the genome distribution results, most clean reads were located in the exonic region, and only a few reads were found in the intergenic and intronic regions in the four-time points (Figure 1C).

### Gene Ontology and KEGG Enrichment Analysis of DEGs

**Figure 2:**
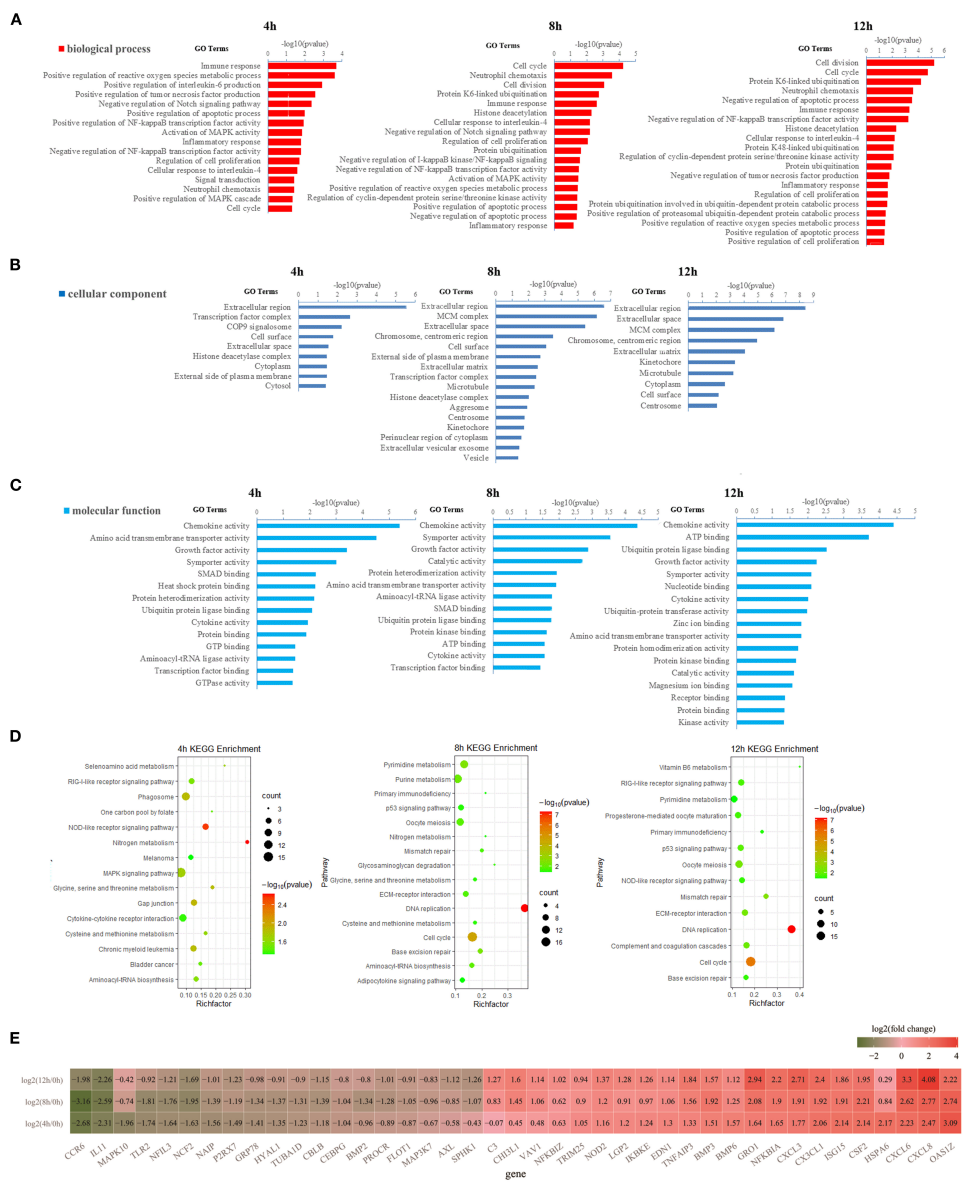
Gene ontology (GO) and Kyoto Encyclopedia of Genes and Genomes (KEGG) enrichment analysis of DEGs. **(A)** The significantly enriched GO terms in the biological process at 4, 8, and 12 h, respectively. **(B)** The significantly enriched GO terms in cellular components at 4, 8, and 12 h, respectively. **(C)** The significantly enriched GO terms in molecular function at 4, 8, and 12 h, respectively. **(D)** The significantly enriched KEGG pathway at 4, 8, and 12 h, respectively. **(E)** The heatmap of differentially expressed genes (DEGs) involved in immune response, inflammatory response, chemokine activity, cytokine activity, NOD-like receptor (NLR) signaling pathway, phagosome, RIG-I-like receptor signaling pathway, and COP9 signalosome. In **(E)**, the dark green represents down-regulationand the red represents upregulation.

About 291, 456, and 379 DEGs at 4, 8, and 12 h compared to 0 h were obtained according to the **|**log_2_(fold change) **|** ≥ 1, *p* < 0.05 and FPKM ≥ 10 in LPS-induced bMECs and 200, 303, and 222 genes were downregulated, whereas 91, 153, and 157 were upregulated at 4, 8, and 12 h, respectively (Supplementary Table 2); more genes were differentially regulated at 8 and 12 h. Then functional enrichment analysis of the DEGs was performed. A total of 39, 47, and 47 GO terms belonging to biological process, cellular component, and molecular function were significantly enriched at 4, 8, and 12 h cells (*p* < 0.05), respectively (Figures 2A–C). It was noteworthy that the immune response (GO:0006955) and positive regulation of reactive oxygen species (ROS) and metabolic process (GO:2000379) were more significantly enriched than cell cycle (GO:0007049) and neutrophil chemotaxis (GO:0030593) at 4 h bMECs (Figure 2A). The ROS is one of the inflammatory mediators, and the release of ROS is an important part of the inflammatory response during dairy mastitis (30). Further, several key terms were enriched simultaneously in 4, 8, and 12 h cells, such as regulation of cell proliferation (GO:0042127), cellular response to interleukin-4 (GO:0071353), negative regulation of NF-κB transcription factor activity (GO:0032088), inflammatory response (GO:0006954), ubiquitin-protein ligase binding (GO:0031625), cytokine activity (GO:0005125), symporter activity (GO:0015293), and chemokine activity (GO:0008009) (Figures 2A–C).

Positive regulation of NF-κB transcription factor activity (GO:0051092), positive regulation of interleukin-6 production (GO:0032755), positive regulation of MAPK cascade (GO:0043410), positive regulation of tumor necrosis factor (TNF) production (GO:0032760), and COP9 signalosome (GO:0008180) were only significantly enriched at 4 h (Figures 2A–C). The NFκB pathway significantly regulated the process of mastitis (9, 31). The TNF plays a key role in the synchronization of the immune response of the mammary parenchyma of cow against mastitis-causing bacteria (18). In contrast, negative regulation of TNF production (GO:0032720), positive regulation of proteasomal ubiquitin-dependent protein catabolic process (GO:0032436), and protein K48-linked ubiquitination (GO:0070936), were only significantly enriched at 12 h cells (Figure 2A). The process of ubiquitination has recently emerged as an important regulator of inflammasome assembly, which is the critical component of the immune system to sense and respond to danger appropriately maintaining immune homeostasis (32).

Meanwhile, KEGG enrichment results of the DEGS indicated that the NOD-like receptor (NLR) signaling pathway (ko04621) is the most significantly enriched at 4 h LPS-induced bMECs compared to 8 and 12 h, while the process of cytokine-cytokine receptor interaction (ko04060) and phagosome (ko04145) are only significantly enriched at 4 h (Figure 2D). As known, NLRs specifically sense pathogen-associated molecular patterns and respond by activating other signaling regulators, including Rip2 kinase, NF-κB, MAPK, and ASC/caspase-1, leading to the secretion of various cytokines (33), which is a response from the early stage of LPS induction. Additionally, the p53 signaling pathway (ko04115) is significantly enriched at 8 h and 12 h cells (Figure 2D). The p53 regulates a wide variety of cellular processes including apoptosis, cell cycle arrest, and acceleration of DNA repair following cellular stress. It seems that bMECs put more focus on balancing the pro-apoptosis and anti-apoptosis at the late stage of LPS-induced bMECs from 8 to 12 h.

### The Differentially Expressed mRNAs Involved in Inflammation and Immunity of bMECs

The expression pattern of genes involved in immune response, inflammatory response, chemokine activity, cytokine activity, NLR signaling pathway, phagosome, RIG-I-like receptor signaling pathway, and COP9 signalosome is displayed in Figure 2E and Supplementary Figure 1. Among these genes, *CCR6, IL11, MAPK10, TLR2, NFIL3, NCF2, NAIP, P2RX7, GRP78, HYAL1, TUBA1D, CBLB, CEBPG, BMP2, PROCR, FLOT1, MAP3K7, AXL*, and *SPHK1* are significantly downregulated [log_2_(fold change) ≤ −1 and *p* ≤ 0.05] after LPS-induction; meanwhile *C3, CHI3L1, VAV1, NFKBIZ, TRIM25, NOD2, LGP2, IKBKE, EDN1, TNFAIP3, BMP3, BMP6, GRO1, NFKBIA, CXCL3, CX3CL1, ISG15, CSF2, HSPA6, CXCL6, CXCL8*, and *OAS1Z* are significantly upregulated [log_2_(fold change) ≥ 1 and *p* ≤ 0.05] (Figure 2E).

For the upregulated genes, the mRNA level of *NOD2, TNFAIP3, GRO1, NFKBIA, CXCL3, CX3CL1, CXCL6*, and *CXCL8* are significantly increasing in turn at 4 h, 8 h, and 12 h bMECs compared to 0 h; the expression of *CXCL8, CXCL6*, and *GRO1* at 12 h are even significantly higher than that at 4 h and/or 8 h {log_2_[12 h/(4 or 8 h)] ≥ 1 and *p* ≤ 0.05} (Supplementary Figure 1). Although the mRNA level of *OAS1Z, CSF2, ISG15, BMP6, BMP3*, and *EDN1* at 4, 8, and 12 h are significantly higher than that at 0 h LPS-induced bMECs [log_2_(fold change) ≥ 1 and *p* ≤ 0.05], they began to regress at 8 or 12 h compared to 4 h, such as *OAS1Z* and *ISG15* began to regress at 8 h {−1 < log_2_[(12 and 8 h)/4 h] <0}, and *CSF2* and *BMP3* began to regress at 12 h {−1 < log_2_[12 h/(8 or 4 h)] <0} (Supplementary Figure 1). Meanwhile, *C3* and *NFKBIZ* are only significantly higher at 12 h, *HSPA6* and *TRIM25* are significantly upregulated at 4 h, *CHI3L1* and *VAV1* are significantly upregulated at 8 and 12 h (Figure 2E; Supplementary Figure 1).

For the downregulated genes, the expression of *CCR6, IL11, NFIL3, NCF2, NAIP, P2RX7*, and *CBLB* at 4, 8, and 12 h are significantly less than that at 0 h cells [log_2_(fold change) ≤ −1 and *p* ≤ 0.05]. *MAPK10* only significantly decreased at 4 h. *AXL* is significantly downregulated at 12 h. *BMP2* and *FLOT1* are significantly reduced at 8 h cells. *TLR2, GRP78, HYAL1, TUBA1D*, and *CEBPG* are significantly downregulated at 4 and 8 h cells, while *SPHK1* and *PROCR* are just not significantly downregulated at 4 h cells (Figure 2E; Supplementary Figure 1).

### The Relative Expression Level of mRNAs by qPCR

**Figure 3:**
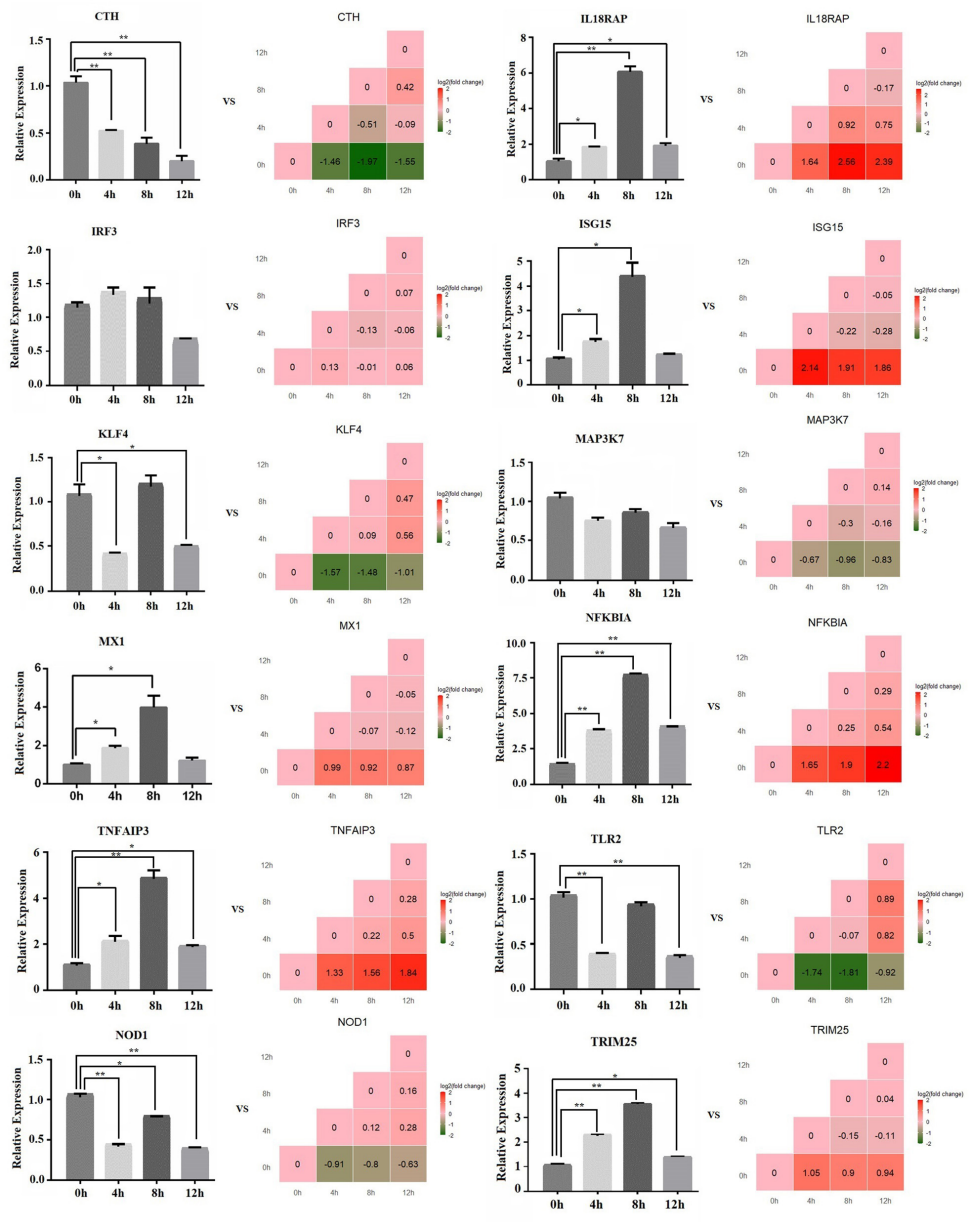
The relative expression level of mRNA by qPCR. The bar graph shows the qPCR result, and the stair graph shows the sequencing result; 0, 4, 8, and 12 h represent the LPS-induced 0, 4, 8, and 12 h bMECs; “*” represents *P* ≤ 0.05; “**” represents *P* ≤ 0.01.

The relative expression of 12 mRNAs was analyzed by qPCR at 0, 4, 8, and 12 h LPS-induced bMECs. Overall, the qPCR results showed an identical trend with the sequencing results in 0, 4, 8, and 12 h cells, *CTH, KLF4, MAP3K7, NOD1*, and *TLR2* decreased, while *IL18RAP, ISG15, MX1, NFKBIA, TNFAIP3*, and *TRIM25* increased (Figure 3), but the discrepancy still exists. For example, the expression of *KLF4* at 8 h was not significantly downregulated and even increased, and TLR2 did not significantly downregulate at 8 h; these discrepancies between the qPCR and sequencing results may be due to experimental errors.

### An Overview of miRNA Profile

To further characterize the transcriptome in bMECs after LPS stimulation, a total of 12.5, 13, 12, and 11 million raw reads of miRNAs were obtained from 0, 4, 8, and 12 h LPS-induced bMECs, and the valid reads accounted for 91.99, 88.84, 87.97, and 80.63%, respectively (Table 2). About 1,215 unique miRNAs were identified including 247 known miRNAs, 460 novel miRNAs, and 508 miRNAs with differences. And the number of types for miRNA was slightly different at 0, 4, 8, and 12 h (Table 3; Supplementary Table 3). Comparing miRNAs and mRNAs, the total expression level of miRNAs increased while mRNA reduced after LPS induction (Table 4).

**Table 2:** Statistics of transcriptomic small RNA reads.

**Lib**	**0 h**		**4 h**		**8 h**		**12 h**	
	**Total**	**% of**	**Total**	**% of**	**Total**	**% of**	**Total**	**% of**
Raw reads	12,501,170	100	13,000,222	100	12,061,098	100	11,216,042	100
Valid reads	11,500,392	91.99	11,549,613	88.84	10,609,997	87.97	9,043,601	80.63

**Table 3:** The number of types for miRNA at 0, 4, 8, and 12 h, respectively.

**Sample**	**Known** **microRNA**	**microRNA** **with differences**	**Novel** **microRNA/predicted** **microRNA**	**Total**
0 h	242	492	411	1,144
4 h	242	497	434	1,173
8 h	243	493	423	1,159
12 h	243	494	406	1,143
Total types	247	508	460	1,215

*1. Novel microRNA, miRNA identified in this study and not reported in miRBase, mainly for newly reported 5p or 3p sequence; 2. MicroRNAs with differences, confirming miRNA sequences in miRBase, but different sequences are reported in our study; 3. Known microRNA, confirming miRNA sequences in miRBase*.

**Table 4:** The total expression level of miRNAs and mRNAs at 0, 4, 8, and 12 h, respectively.

**Sample**	**miRNAs/copies**	**mRNAs/FPKM**
0 h	6,500,331	797,968
4 h	7,487,961	785,310
8 h	7,179,674	776,069
12 h	7,518,120	740,538

*FPKM presents fragment per kilobase of exon per million fragments mapped*.

**Figure 4:**
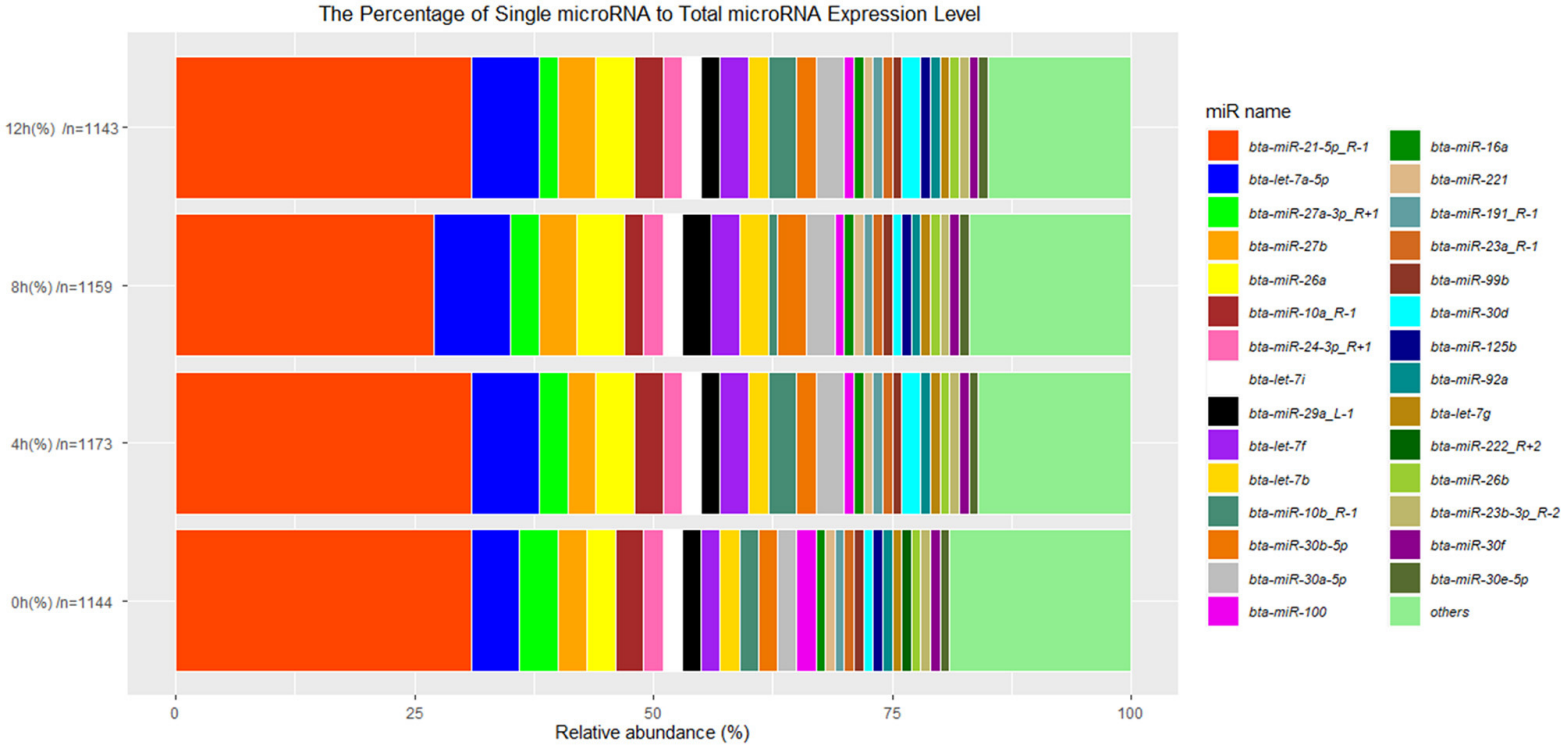
The 29 top-expressed miRNAs at 0, 4, 8, and 12 h LPS-induced bMECs. “*n*” represents the number of identified miRNAs at 0, 4, 8, and 12 h LPS induced bMECs.

Additionally, 29 top-expressed miRNAs account for 81, 84, 83, and 85% in the expression of total miRNAs at 0, 4, 8, and 12 h cells, respectively (Figure 4), which are similar to the previous results in bovine MAC-T cells challenged with *E. coli* or *S. aureus* (16). The expression of *bta-miR-21-5p_R-1* is more than 30% in total miRNAs and is the highest one (Figure 4), which is also in line with the result in primary bMECs (15), while *bta-miR-21-5p_R-1* is not the highest expressed miRNAs at peripheral blood in Chinese Holstein cows (23, 24), bovine milk-derived exosomes (22), bovine milk (21), and bovine mammary glands (19). This indicated that *bta-miR-21-5p_R-1* was the unique and essential miRNA to maintain the function of bMECs.

### Differentially Expressed miRNAs and Inflammation/Immunity-Related Differentially Expressed miRNAs

**Figure 5:**
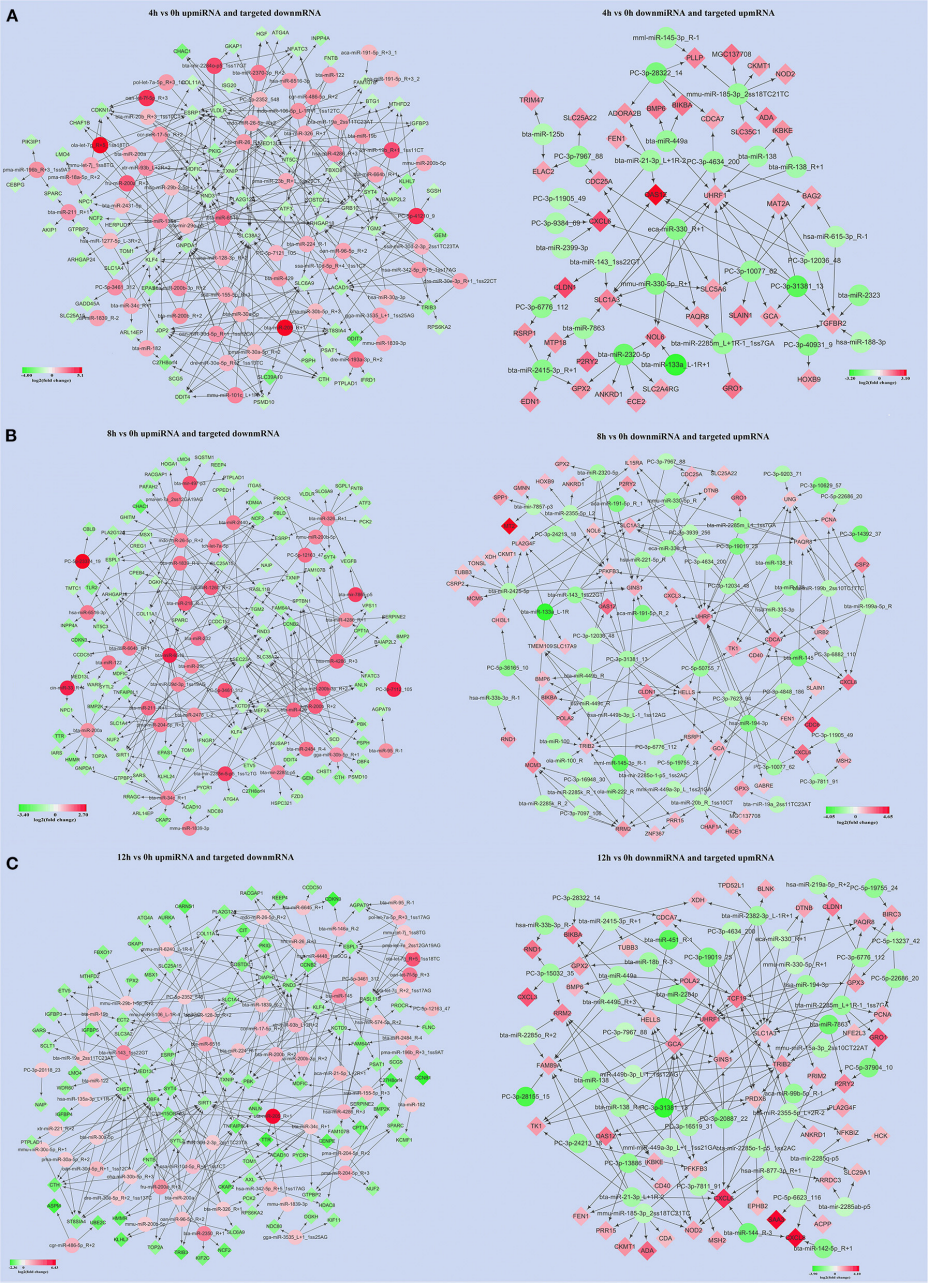
The significantly up/downregulated miRNAs along with significantly down/upregulated targeted mRNAs were screened out at 4, 8, and 12 h LPS-induced bMECs compared to 0 h, respectively. **(A)** The results were at 4 h; **(B)** the results were at 8 h; **(C)** the results were at 12 h. Green represents downregulation and red represents upregulation.

About 119, 112, and 128 significantly DEMs at 4, 8, and 12 h LPS-induced bMECs compared to 0 h were obtained (Supplementary Table 4), and genes potentially targeted by DEMs were predicted. Together with the DEMS (Supplementary Table 2), 65 upregulated miRNAs along with 71 downregulated targeted mRNAs and 29 down-regulated miRNAs along with 39 upregulated targeted mRNAs at 4 h (Figure 5A), 39 upregulated miRNAs along with 106 downregulated targeted mRNAs, and 57 down-miRNAs along with 61 upregulated targeted mRNAs at 8 h (Figure 5B), 63 upregulated miRNAs along with 92 downregulated targeted mRNAs and 48 downregulated miRNAs along with 54 upregulated targeted mRNAs at 12 h (Figure 5C) were screened out in LPS-induced bMECs.

**Figure 6:**
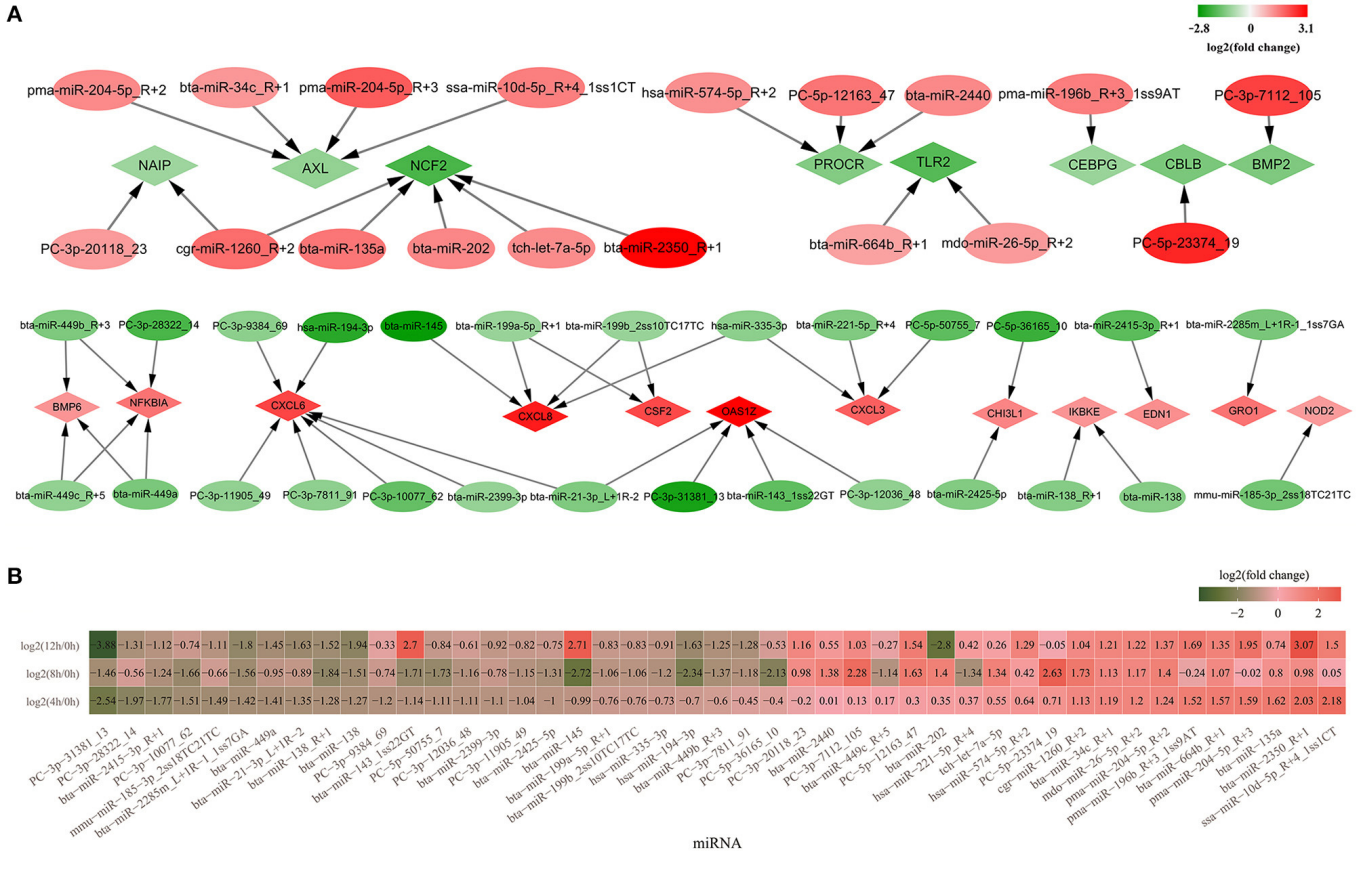
The differentially expressed miRNAs involved in inflammation and immunity. **(A)** Forty-five significantly up- or down regulated miRNAs involved in inflammation and immunity and their targeted mRNAs. Diamond represents mRNAs and the oval represents miRNAs; **(B)** The heatmap of relative expression for 45 significantly up/downregulated miRNAs in 4, 8, and 12 h bMECs. Green represents the downregulation and red represents the upregulation.

Subsequently, 18 significantly upregulated and 27 significantly downregulated miRNAs involved in inflammation and immunity were picked out. These miRNAs targeted 8 significantly downregulated mRNAs (*CBLB, CEBPG, PROCR, TLR2, BMP2, NCF2, AXL*, and *NAIP*) and 12 upregulated mRNAs (*OAS1Z, BMP6, GRO1, CHI3L1, CXCL6, CXCL3, CXCL8, CSF2, EDN1, NFKBIA, NOD2, IKBKE*) (Figure 6A). The expression levels of the 45 significantly up/downregulated miRNAs were analyzed (Figure 6B; Supplementary Figure 2).

Among the 27 significantly downregulated miRNAs, *PC-3p-31381_13, bta-miR-2415-3p_R*+*1, bta-miR-2285m_L*+*1R-1_1ss7GA, bta-miR-138_R*+*1*, and *bta-miR-138* significantly decreased throughout the LPS induction period. *PC-3p-10077_62, PC-5p-50755_7, PC-3p-12036_48, PC-3p-11905_49, bta-miR-2425-5p, bta-miR-145*, and *bta-miR-143_1ss22GT* were significantly downregulated at 4 and 8 h. Moreover, it was suddenly increased at 12 h cells for *bta-miR-143_1ss22GT* [log_2_(12/0 h) = 2.7, log_2_(12/4 h) = 3.7, log_2_(12/8 h) = 4.41] and *bta-miR-145* [log_2_(12/0 h) = 2.71, log_2_(12/4 h) = 3.84, log_2_(12/8 h) = 5.43] (Figure 6B; Supplementary Figure 2). *Hsa-miR-194-3p, bta-miR-449b_R*+*3*, and *PC-3p-7811_91* were downregulated at 8 and 12 h cells. *PC-3p-9384_69* and *bta-miR-2399-3p* were significantly decreased only at 4 h, while *bta-miR-199a-5p_R*+*1, bta-miR-199b_2ss10TC17TC, hsa-miR-335-3p, PC-5p-36165_10, bta-miR-449c_R*+*5*, and *hsa-miR-221-5p_R*+*4* significantly went down at 8 h cells. There is a deviation in the expression levels of *PC-3p-28322_14, mmu-miR-185-3p_2ss18TC21TC, bta-miR-449a*, and *bta-miR-21-3p_L*+*1R-2*, which were decreased significantly at 4 and 12 h corresponding to 0 h, but not at 8 h cells (Figure 6B; Supplementary Figure 2).

For the 18 significantly upregulated miRNAs, *bta-miR-664b_R*+*1, pma-miR-204-5p_R*+*2, mdo-miR-26-5p_R*+*2, bta-miR-34c_R*+*1*, and *cgr-miR-1260_R*+*2* were significantly higher at 4, 8, and 1 2h. *PC-3p-7112_105* and *PC-5p-12163_47* were significantly increased from 8 to 12 h. But the expression levels of *pma-miR-196b_R*+*3_1ss9AT, pma-miR-204-5p_R*+*3, bta-miR-2350_R*+*1*, and *ssa-miR-10d-5p_R*+*4_1ss1CT* at 8 h differed from that at 4 and 12h, which were not significantly upregulated at 8 h LPS-induced cells (Figure 6B). There is a striking difference for *bta-miR-202*, which was significantly increased at 8 h [log_2_ (8/0 h) = 1.4] while significantly reduced at 12 h cells [log_2_ (12/0 h) = −2.8; log_2_ (12/4 h) = −3.15; log_2_(12/8 h) = −4.2] (Supplementary Figure 2). In a word, there is a great variability for the expression level of several miRNAs during LPS induced bMECs.

### The Relative Expression Level of miRNAs by qPCR

**Figure 7:**
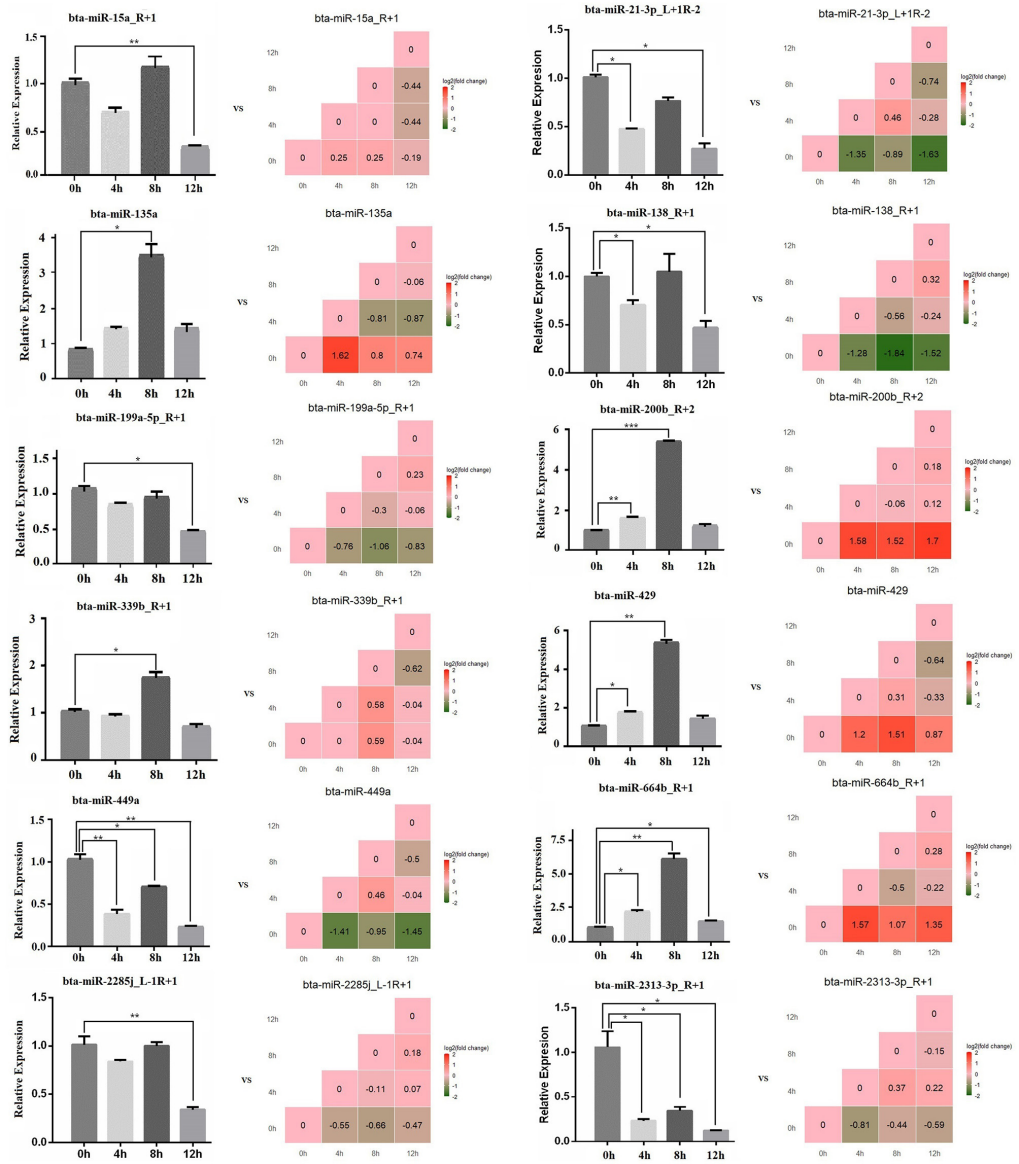
The relative expression level of microRNA by qPCR. The bar graph is the qPCR result, and the stair graph is the sequencing result; 0, 4, 8, and 12 h represent the LPS-induced 0, 4, 8, and 12 h bMECs; “*” represents *P* ≤ 0.05; “**” represents *P* ≤ 0.01; “***” represents *P* ≤ 0.001.

The expression level of 12 miRNAs (*bta-miR-664b_R*+*1, bta-miR-21-3p_L*+*1R-2, bta-miR-2313-3p_R*+*1, bta-miR-2285j_L-1R*+*1, bta-miR-199a-5p_R*+*1, bta-miR-339b_R*+*1, bta-miR-429, bta-miR-200b_R*+*2, bta-miR-135a, bta-miR-15a_R*+*1, bta-miR-138_R*+*1*, and *bta-miR-449a*) were measured in LPS-induced bMECs by qPCR. We found that the qPCR results coincide with the sequencing results in LPS-induced bMECs, although there are still some deviations between sequencing and qPCR results. For example, the expression of *bta-miR-15a_R*+*1* significantly decreased at 12 h by qPCR while not by sequencing, *bta-miR-135a* significantly increased at 8 h by qPCR and it went up at 4 h by sequencing; *bta-miR-138_R*+*1* was significantly reduced after LPS induction by sequencing while not at 8 h cells by qPCR. These deviations may be due to two reasons, the one is the experiment error, and the second is that significance criteria are inconsistent in the processing of sequencing data and quantitative data by different software (Figure 7).

### The GO Terms Related to Mammary Development and Milk Synthesis in LPS-Induced BMECs

**Figure 8:**
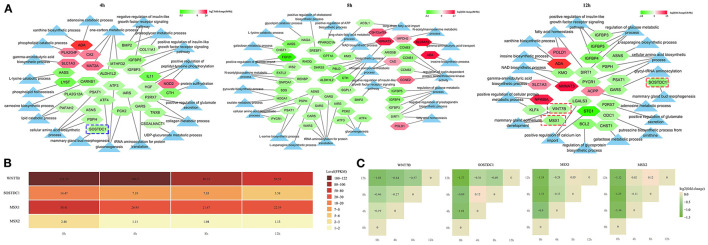
The expression network of the nutrition metabolism pathway-related genes. **(A)** The light blue triangle represents GO term related to the nutrition metabolism; the pentagon represents the differentially expressed mRNAs related to the nutrition metabolism pathway; **(B)** The expression level in fragment per kilobase of exon (FPKM), of 4 genes in LPS-induced bMECs; **(C)** The stair graphic of the relative differential relationship for 4 genes between different timepoints, such as log2(12/0 h), log2(8/0 h), log2(4/0 h), log2(12/8 h), log2(12/4 h), and log2(8/4 h) values. 0, 4, 8, and 12 h represent the LPS-induced 0, 4, 8, and 12 h bMECs; Green represents downregulation and red represents upregulation in **(A,C)**.

The GO terms about the nutrition metabolism were also screened out for analyzing the milk synthesis ability of bMECs during the LPS-induced period. The number of significantly downregulated mRNAs was far more than the upregulated (Figure 8A), including 26 down- and 6 upregulated mRNAs at 4 h, 42 down- and 8 upregulated mRNAs at 8 h, and 20 down- and 6 upregulated mRNAs at 12 h. Especially at 8 h, most of the nutrition metabolism related-mRNAs showed a significant decrease, which resulted in inactive biosynthetic processes, such as the cellular amino acid biosynthesis process (GO:0008652), gluconeogenesis (GO:0006094), pyruvate biosynthetic process (GO:0042866), L-asparagine biosynthesis process (GO:0070981), L-serine biosynthesis process (GO:0006564), long-chain fatty acid metabolic process (GO:0001676), regulation of glucose metabolic process (GO:0010906), positive regulation of glucose import (GO:0046326), and galactose metabolic process (GO:0006012) (Figure 8A).

Notably, the *SOSTDC1* gene enriched at the mammary gland bud morphogenesis (GO:0060648) was significantly downregulated at 4 h and 12 h (Figures 8B,C). SOSTDC1 regulates the number and size of mammary buds (34). The mammary gland bud morphogenesis is important for the early stages of the mammary pool and mammary duct development that will directly determine the lactation capacity of the mammary. Furthermore, the *WNT7B* and *MSX1* genes enriched in the mammary gland epithelium development (GO:0061180) were significantly downregulated at 12 h cells (Figures 8B,C). During puberty, the expression of WNT7B is enriched in the terminal end bud epithelium and plays a role in mammary duct branching morphogenesis (35). MSX1 and MSX2 appear to have necessary but redundant functions during the formation of the buds (36). In the present study, *MSX2* is also significantly decreased after LPS induction although the expression level is lower than the *MSX1* gene (Figures 8B,C).

These results demonstrated that the milk synthesis ability of bMECs will be undermined after LPS challenge and *SOSTDC1, WNT7B, MSX1*, and *MSX2* may be the important regulated genes for lactation capacity.

### The Expression of TLR Signaling Cascades in LPS-Induced bMECs

**Figure 9:**
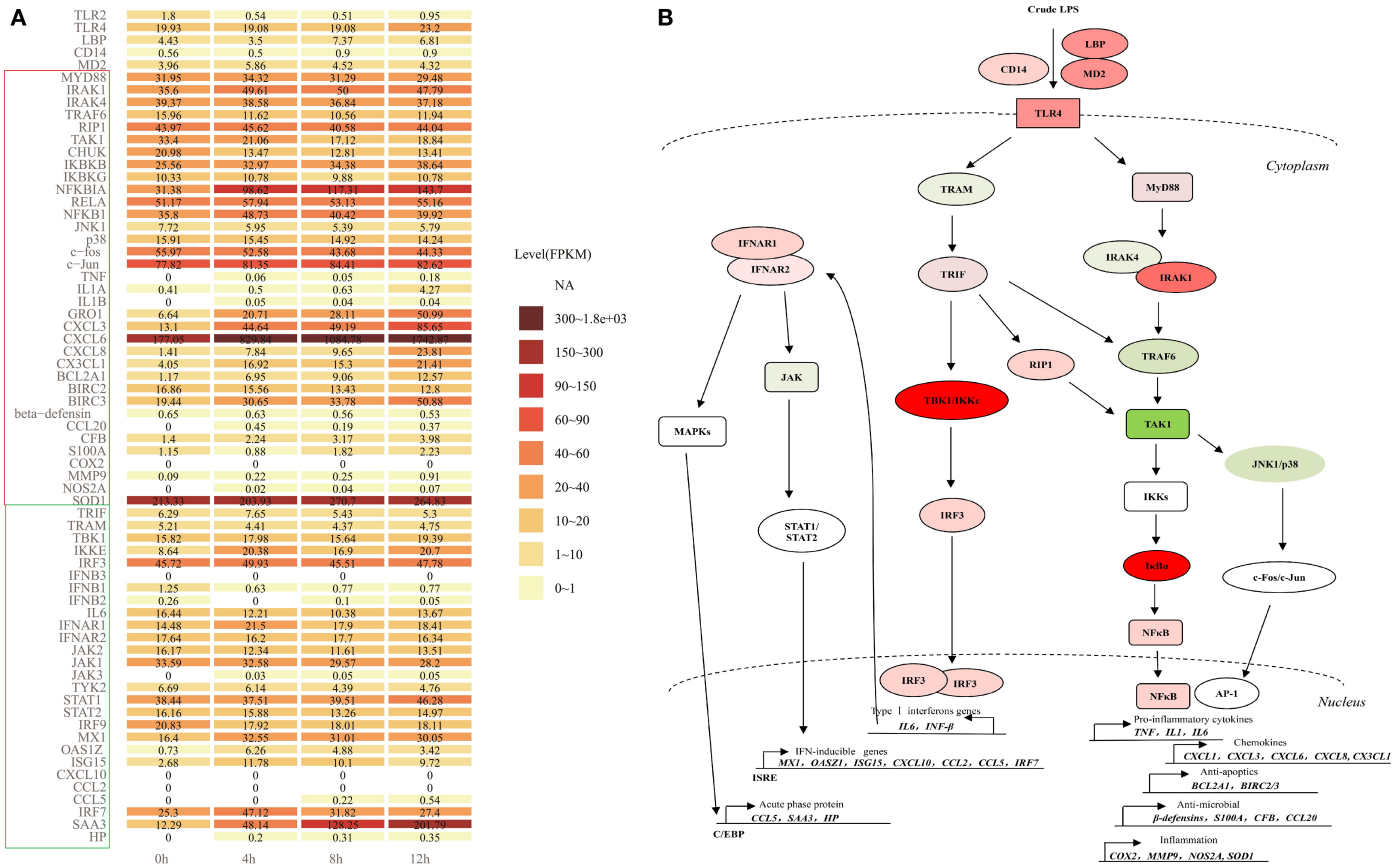
The TLR4 signaling cascades in LPS-induced bMECs. **(A)** The expression level (FPKM) of all members involved in myeloid differentiation factor 88 (MyD88)-dependent and independent pathway in LPS-induced bMECs; **(B)** TLR4 signaling following MyD88- dependent and independent pathways. First, signaling *via* MyD88 subsequently recruits IRAK1, IRAK4 (IL1R associated kinase family), and TNF receptor-associated factor 6 (TRAF6), forms a complex with TAK1 (mitogen-activated protein kinase kinase kinase 7, MAP3K7), then activate TAK1, which phosphorylates IκB kinases (IKKs, IKK1/IKK2/NEMO complex) and MAP kinases (e.g., JNK, p38). TAK1 can also be induced by receptor-interacting protein 1 (RIP1). IKKs phosphorylate the IκBα, then leads to IκBα degradation, enabling the nuclear translocation of NFκB, ultimately beginning the transcription activation of pro-inflammatory cytokines (TNFα, IL1, and IL6), chemokines (GRO1, CXCL3, CXCL6, CXCL8, and CX3CL1), anti-apoptotic genes (BCL2A1 and BIRC2/3), anti-microbial genes (β-defensins, S100A, CFB, and CCL20) and inflammatory genes (COX2, MMP9, SOD, and NOS2A). Another pathway AP-1 transcription factor formed by c-Fos and c-Jun can be activated by JNK1/p38 complex, then initiate the production of cytokines and chemokines like NF-kB signaling. Second, the TRIF-dependent cascade through the bridging adaptor, TRIF-related adaptor molecule (TRAM) by the delayed mode in either a TRAF6 or RIP1-dependent manner activate TAK1 and trigger NFκB and AP-1 pathway. TRIF also interacts with TRAF-associated NFκB activator-binding kinase 1 (TBK1) and IκB kinase epsilon (IKKε), which phosphorylate IRF3, leading to induction of typeIIFN genes (IFNβ/IL6). The IL6 or IFNβ binds to its receptor, IFNAR1/2 causing the activation of JAKs, eventually activating STAT1. The STAT1 binds as a homodimer to the gamma interferon-activating sequence or trimerizes with STAT2 and IRF9 to form interferon-stimulated transcription factor 3 (ISGF3), which can bind to interferon-stimulated response element (ISRE)-mediated expression of secondary response genes, such as MX1, OASZ1, ISG15, CXCL10, CCL2, CCL5, and IRF7. Binding IFNAR1/2 also activates MAPKs, then trigger C/EBPβ to induce the expression of acute-phase proteins, such as CCL5, SAA3, and HP. Red represents increased expression; green represents decreased expression in **(B)**.

Crude LPS also contains bacterial lipoproteins and stimulates both the TLR4 and TLR2 activation pathways (9). In the present study, TLR2 significantly decreased, and the expression level is low in LPS-induced bMECs, while the expression level of TLR4 is much higher with a slight increase at 12 h (Figure 9A). So, TLR4 pathway may be the main regulatory signaling in bMECs after the crude LPS challenge.

Here, we display the expression level of all the members involved in TLR4 signaling cascades (Figure 9A). After LPS induction, bMECs should respond to the stimulation through the TLR4 pathway following MyD88-dependent and TRAM/TRIF-dependent signaling cascades (13) (Figure 9B). For extracellular signal transduction, LPS-signal presenting molecules *LBP, CD14, MD2*, and *TLR4* all showed increased expression.

The expression of *MyD88, IRAK1, IRAK4*, and *TRAF6* changed non-significantly, while the hub molecular *TAK1* for AP-1 and NFκB pathways was significantly downregulated (Figure 2E). The *RIP1* shows no significant change but with a slight increase (Figure 9A). The expression levels of *IKBKB, NFKBIA, RELA*, and *NFKB1* increased, while that of *CHUK* (*IKK1*) was decreased. Kinases IKK1 is believed to be a negative regulator of TLR signaling by sequestering NEMO (IKBKG) away from IKK2 (IKBKB), and IKK1 knockout cells show overt inflammatory cytokine production (37). So decreased *CHUK* promotes inflammation in LPS-induced bMECs. In contrast, AP-1 pathway is less active, because the kinases complex *JNK1/p38* and *c-fos* all were decreased, although *c-Jun* slightly was increased (Figure 9A). Thus, the downstream response genes are mainly induced by the transcription factor NF-κB (Figure 9B).

Pro-inflammatory cytokines (*IL1*α, *IL1*β, and *TNF*α) were significantly increased but the expression level was lower in induced cells, especially *IL1*α. The expression of *IL6* was relatively higher in control cells, but it decreased after the LPS challenge. Chemokines *GRO1, CXCL3, CXCL6, CXCL8*, and *CX3CL1* significantly upregulated after LPS induction and kept higher expression, particularly *CXCL6*. The anti-apoptotic effect may be significantly active because *BCL2A1* and *BIRC3* significantly increased with a higher expression. The anti-microbial and inflammatory effects were also activated but weak because the involved genes significantly increased while in a low expression, such as *CCL20, CFB, S100A, MMP9*, and *NOS2A*. The β*-defensin* even slightly decreased (Figure 9A). After the LPS was challenged, the oxidative stress activity of bMECs is strengthened with the increasing *SOD1* (Cu, Zn-superoxide dismutase) (Figure 9A), which helps protect mitochondria from oxidative damage during disease.

After LPS induction, *TRIF* increased at 4 h LPS-induced bMECs but decreased at 8 h and 12 h cells, and *TRAM* slightly decreased after LPS induction (Figure 9A). *TBK1* and *IRF3* increased but not significantly and with a high expression level at bMECs (Figure 9A), while *IKBKE* significantly increased (Figure 2E; Supplementary Figure 1). However, the primary response genes *IL6, IFNB1*, and *IFNB2* showed decreased, *IL6* with a relatively higher expression as previously mentioned (Figure 9A). The basic existing IL6 or IFNB in bMECs will bind to its receptor, IFNAR1 and IFNAR2 causing the activation of the Janus tyrosine kinases (JAKs), eventually activating STAT1 (Figure 9B). The expression of *JAK1, JAK2*, and *TYK2* decreased, while *JAK3* was expressed in trace after LPS induction. The *STAT1* is slightly increased from LPS-induced 8 h cells (Figure 9A).

The STAT1 binds as a homodimer to the gamma interferon-activating sequence or trimerizes with STAT2 and IRF9 to form ISGF3 (interferon-stimulated transcription factor 3), which will mediate the expression of IFN-inducible secondary response genes (Figure 9B). *STAT2* and *IRF9* slightly decreased with relatively higher expression, while secondary response genes, such as *MX1, OASZ1, ISG15, CCL5*, and *IRF7* increased; apparently, *CXCL10* and *CCL2* were not detected. Moreover, *SAA3* and *HP* also significantly increased, especially *SAA3* with a high expression level (Figure 9A). The chemokines, CXCL10, CCL2, and CCL5 are optimal productions of the type I IFN pathway, which is a part of the “type I IFN chemokine signature”, and mainly attract monocytes, natural killer cells, and activated lymphocytes (38). We found the NFκB dependent- chemokine *CXCL1, CXCL3, CXCL6, CXCL8*, and *CX3CL1* are early and strongly induced by the LPS in our bMEC model. Only at later times did LPS induce *CCL5*, but not *CCL2* and *CXCL10* (Figure 9A).

In brief, bMECs mainly play a role in secreting chemokines for recruiting the neutrophil to fight against pathogens and anti-apoptosis for protecting themselves from dying during mastitis; they were charged with weak anti-microbial and proinflammatory effects. The NF-κB pathway plays a key role in recruiting neutrophils, not the Type I IFN pathway for bovine mammary epithelial cells (bMECs).

## Discussion

Mastitis causes significant losses in the dairy industry and its susceptibility is a multifactorial complex phenotype, which is not the result of single genes acting in isolation but rather is attributable to perturbation at a network or systems level. Heritability for mastitis traits (clinical, subclinical, SCC) is relatively low (from 0.003 to 0.18); genetic selection for mastitis resistance is nevertheless has been demonstrated to reduce the incidence of mastitis, particularly when genetic markers of mastitis traits are used (39). The bovine mammary epithelium is the first line of defense after a bacterial infection. Revealing the functional mechanism of mammary epithelial cells during mastitis will help to find key mastitis of genetic marker molecules.

### The Molecules Regulated TLR Signaling in LPS-Induced bMECs

The expression of *TLR2* is differentially upregulated in *E. coli-*incubated pbMECs (8), but it is significantly downregulated either by qPCR or sequencing in LPS-induced bMECs (Figure 3), which may be due to the fact that crude LPS mainly triggers the TLR4 signaling while not the TLR2 signaling. Nevertheless, most of the genes showed a consistent expression trend between MAC-T cells induced by LPS and pbMECs induced by *E. coli*, such as *GRO1, CXCL3, CX3CL1, CXCL6, CXCL8, OAS1Z, NFIL3, TNFAIP3, IKBKE, NFKBIZ, CHI3L1, C3, MX1*, and *ISG15* (8).

The proinflammatory cytokines, TNFα and IL1β are key components of the innate immune response to infection. They accelerate the inflammatory response either by directly or indirectly regulating the functions of infiltrating neutrophils, monocytes, and the cytokines produced by these cells. Although the mRNA of *TNF*α and *IL1*β were significantly augmented upon stimulation with LPS/*E.coli*, and to some extent upon stimulation with SaS/ heat-inactivated *S. aureus* in primary bMECs (8–10). Gilbert et al. demonstrated that pbMECs are unable to secrete TNFα and IL1β at protein level by detecting the supernatants of bMEC incubated with LPS or with SaS (9). In this study, the expression level of *TNF*α and *IL1*β are relatively low but increase after LPS treatment (Figure 9A).

In addition, the secretion of IL-1β is not equivalent to expression at the RNA level, as secretion requires cleavage of pro-IL-1β by activated caspase-1, which results from inflammasome activation. Meanwhile, activation of purinergic P2X7 receptors (P2X7R) by extracellular ATP is a key physiological inducer of rapid IL-1β release. P2X7R antagonism prevents IL-1β release from salivary epithelial cells and reduces inflammation in a mouse model of autoimmune exocrinopathy (40). The P2X7R significantly decreased after LPS induction in bMECs (Figure 2E), Overall, TNFα and IL1β are likely to be mainly produced by the resident or recruited leucocytes rather than by MEC in mammary during mastitis.

The *CBLB* is a TLR4 antagonist, which can degrade phosphorylated MyD88 and TRIF, then inhibits TLR signaling in innate immune responses (41). Significantly decreased *CBLB* may promote the MyD88- and TRIF-dependent signaling in LPS-induced bMECs (Figures 2E, 9B).

The *bta-miR-138* and *bta-miR-138_R*+*1* were predicted to target on the 3′UTR of *IKBKE*, and the expression level of the two miRNAs was significantly downregulated. The level of *miR-138* was markedly reduced in the cartilage tissues of osteoarthritis and the overexpression of *miR-138* suppressed the protein levels of p65 (42), which is the key transcription factor of the NFκB pathway. Correspondingly, the expression level of the mRNA *p65* (*RELA*) was increased after LPS induction in bMECs (Figure 9A). Upregulation of *miR-143-3p* expression reduced TNFα, MyD88, p50, and alveolar epithelial cell apoptosis in *mycoplasma pneumonia* mice by inhibiting TLR4/MyD88/NF-κB axis (43). The *bta-miR-143_1ss22GT* is predicted to target *OAS1Z* (Figure 6A). decreased *bta-miR-143_1ss22GT* may promote TLR4/MyD88/NF-κB cascade and increase the expression of *TNF*α, and *OAS1Z* in LPS-induced bMECs (Figures 9A,B).

### The Chemokines and Related miRNAs of bMECs After LPS Induction

Mammary epithelial cells (MECs) challenged by a bacterial infection should have the capacity to attract polymorphonuclear leukocytes into mammary tissue and mount an innate immune response. After LPS-challenged, the early chemokine and cytokine production preceded the defense and stress responses in bMECs. bMECs can secrete the chemokines, such as *GRO1*(*CXCL1*), *CXCL3, CXCL6*, and *CXCL8*, which have strong chemoattractant activities that attract mainly neutrophils. All of the chemokines showed significant and rapid increases in MECs after LPS treatment (Figure 2E; Supplementary Figure 1). These results are generally consistent with the results in cow udders infected with *E. coli* (44) and *S. aureus* (18), as well as in the LPS-induced primary MECs (9). In particular, *CXCL6* had the highest expression level among chemokines in bMECs. The *CXCL6* usually significantly altered the expression level at bMECs after infection (6, 8, 45) but not at mammary tissues with mastitis (18, 46). The C-X3-C motif chemokine ligand 1 (CX3CL1) is a unique chemokine functioning both in a transmembrane and soluble form. The transmembrane form of CX3CL1 primarily serves as an adhesion molecule, but when cleaved to a soluble form, CX3CL1 predominantly functions as a chemotactic cytokine (47). After LPS induction, *CX3CL1* showed a significant increase at 4, 8, and 12 h (Figure 2E), and may serve a unique role during the inflammation of bMECs.

On the contrary, the miRNAs targeting these chemokines, such as *bta-miR-221-5p_R*+*4–CXCL3* axis, *hsa-miR-335-3p–CXCL3/CXCL8* axis, *bta-miR-145–CXCL8* axis, *bta-miR-199a-5p_R*+*1–CXCL8* axis, *bta-miR-199b_2ss10TC17TC–CXCL8* axis, *bta-miR-21_3p_L*+*1R-2–CXCL6* axis, *hsa-miR-194-3p–CXCL6* axis, etc., significantly decreased (Figure 6A). Downregulation of *miR-221-5p* significantly promoted the expression of TNFα, IL8, and IL1β during acute gouty arthritis, and *IL1*β was proved to be targeted by *miR-221-5p* (48). *MiR-335-5p* elevation inhibited the inflammation, as evidenced by decreased levels of TNFα, IL6, and IL8 (49). The LPS repressed the transcription of *miR-143/145* cluster and decreased the *miR-145* levels; attenuation of *miR-145* activity triggered inflammation and increased serum chemokines in C57BL/6J mice (50).

Both *miR-199a-3p* and *miR-199b* can control apoptosis and inflammation by targeting IKKβ to regulate the NF-κB pathway (51, 52). In this study, decreased *bta-miR-199a* and *bta-miR-199b* may relieve the inhibition effects of IKKβ to trigger NF-κB signaling, increasing the *IKK*β (*IKBKB*) with the LPS-induced time (Figure 9A). *STAT3* was a direct target of *miR-21-5p*, and *miR-21-5p* can mediate the IL-6/STAT3 pathway to regulate the levels of inflammation and apoptosis (53). In the present study, downregulated *bta-miR-21* and increased *STAT3* may be another reason for significantly upregulated IFN-inducible genes.

In short, the upregulation of chemokines and downregulation of miRNAs may promote the recruitment of neutrophils in the mammary tissue. Increased neutrophils can mount a rapid non-specific phagocytic response and respiratory burst activity that kills invading bacteria.

### The Phagocytosis in bMECs After LPS Induction

The neutrophil cytosolic factor 2 (*NCF2*) and tubulin alpha-1D chain (*TUBA1D*) are two molecules involved in phagocytosis which are significantly decreased after LPS induction (Figure 2E). The NCF2 is known to participate in ROS metabolism-regulating neutrophil respiratory burst (54). Tubulin is the major constituent of microtubules that facilitate the maturation of phagosomes and control the phagosome movement and shape; reduced *TUBA1D* is not conducive to stabilizing phagosome structures for playing phagocytosis. Epithelial cells engage in phagocytosis, which is an essential defense mechanism of innate immunity, but phagocytosis is not the salient property of bMECs after LPS stimulation.

Decreased *NCF2* may be controlled by significantly upregulated *cgr-miR-1260_R*+*2, bta-miR-2350_R*+*1, bta-miR-202, bta-miR-135a, tch-let-7a-5p*, respectively (Figure 6A). Among them, *miR-135a* overexpression inhibited p-p65 levels and restrains NF-κB activation in lung tissues by targeting TLR4 to alleviate inflammatory response (55). Increased bta-miR-135a may be one of the factors that balances the NF-κB pathway activity in LPS-induced bMECs. High *miR-202-5p* expression increased proliferation and prevented apoptosis and autophagy in oxygen-glucose deprivation/reoxygenation-treated N2a cells (56). Increased *bta-miR-202* may also prevent the apoptosis of LPS-induced bMECs. *Let-7a* inhibitor significantly reduced the expression level of TNFα, IL1β, IL6, and IL8 during LPS-induced cartilage inflammatory injury (57). Upregulated *bta-let-7a-5p* and *tch-let-7a-5p*, especially *bta-let-7a-5p*, which is the second top-expressed miRNA (Figure 4), may play essential roles in increased *TNF*α, *IL1*β, and *IL8* in LPS-induced bMECs.

### The Anti-inflammatory and Anti-apoptotic Activity of LPS-Induced bMECs

Endothelial protein C receptor (EPCR, encoded by *PROCR* gene) plays a key role in the protein C anticoagulant pathway, and the procoagulant clotting factor VIIa (FVIIa) can bind to EPCR and elicits anti-inflammation *via* suppression of TNFα- and LPS-induced expression of cellular adhesion molecules and IL6, while inhibition of EPCR can abolish the anti-inflammatory effect (58). Here, *PROCR* gene is decreased which may lead to the weakened anti-inflammatory effect of EPCR in LPS-induced bMECs (Figure 2E). Interleukin 11 (IL11) is a pleiotropic cytokine with antiapoptotic and anti-inflammatory; it can attenuate IL1-mediated catabolic and inflammatory processes (59). Significantly downregulated *IL11* (Figure 2E) may play a weak role in anti-apoptosis and anti-inflammatory effects in LPS-induced bMECs.

Sphingosine kinase 1 (SPHK1) catalyzes the formation of sphingosine-1-phosphate (S1P), which is essential for the production of the multifunctional NFκB-regulated cytokine IL6 (60). Significantly downregulated *SPHK1* may result in decreased expression of *IL6*, but the basal existing IL6 also can induce the second response genes of the Type I IFN pathway, such as *ISG15, MX1, OAS1Z, IRF7* (Figures 9A,B). The AXL is a cell surface receptor tyrosine kinase; activation of AXL attenuates neuroinflammation by inhibiting the TLR/TRAF/NF-κB pathway (61). In the present study, downregulated *AXL* may promote the NF-κB pathway in bovine MECs.

Neuronal apoptosis inhibitory protein (NAIP) is a member of both IAPs (inhibitory apoptosis proteins) and NLR families and has two different biological functions: pro-inflammatory and anti-apoptosis (62). The impaired regulation of apoptosis is considered to be a prominent event in the development and progression of tumor cells. As a result, significantly reduced *NAIP* will promote the death of bMECs and prevent them from being developed into tumor cells. The NOD2 is another member of NLR family and was proved to prevent inflammation (63). Significantly downregulated *NAIP* and upregulated *NOD2* were beneficial for the anti-inflammatory effects of bMECs after LPS treatment.

Oxidative stress molecule, *HSPA6* significantly and rapidly rises at 4 h, then has a sharp decrease at 8 h cells (Figure 2E), which demonstrated that *HSPA6* is an “early response” factor for LPS-induced bMECs. The *HSPA6* is also significantly upregulated in circulating leucocytes of clinical mastitis, but not in the subclinical mastitis (25). The key member *C3* of the canonical and alternative route of the complement system is significantly upregulated at 12 h cell (Supplementary Figure 1). In Gilbert et al.'s (9) research, *C3* was significantly downregulated in *S. aureus* culture supernatant (SaS)-induced 6 h bMECs, but not significantly differentially expressed in SaS-induced 3 h and LPS-induced 3 and 6 h bMECs. The *C3* seems to be a “late response gene” after infection.

Both CCL20 and β-defensins display antimicrobial activity and bind to the CCR6 receptor, which is also known as macrophage inflammatory protein-3α (MIP-3α). The bMECs can express CCR6, but it was rapidly and significantly downregulated after they were LPS-induced (Figure 2E). Notably, the low upregulation of CCL20 disagrees with its strong induction of bMECs/pbMECs response to LPS (9) or *E. coli* (5, 8), which could be a MAC-T cell bias. This result represents the weak protective mechanism against the invading bacteria of MAC-T cells.

Colony-stimulating factor 2 (*CSF2*) plays a critical role in the resolution of inflammatory responses as well as in the development of chronic inflammation (64) by promoting the differentiation and maturation of macrophages. The *CSF2* was significantly upregulated at the beginning of LPS induction (Figure 2E), which was also confirmed in Gilbert et al.'s (9) research at LPS but not in SaS-induced bMECs.

### NFKBIA Is the Hub Gene for Balancing the Inflammatory Response of bMECs After LPS Induction

The intensity and duration of NFκB and Type I IFN pathway activation have to be controlled to avoid excessive inflammatory tissue damage, and a range of regulatory factors are set in motion to restrict activation, such as increased *NFKBIA* (IκBα) after LPS challenge (Figure 2E). The IκBα acts as the most important and key suppressor of NFκB pathway; increased IκBα will suppress the activity of NFκB pathway during inflammation response. Meanwhile, LPS can induce the degradation of the negative regulator IκBα, allowing for the translocation of p65/p50 into the nucleus, which in turn triggers a negative feedback loop by promoting the resynthesis of IκBα through NF-κB (65). Unlike *NFKBIZ*, which is significantly increased at post-induction 12 h and is “the late response gene”, *NFKBIA* is “the early response gene” (Figure 2E). The expression of *NFKBIA* significantly increased in *staphylococci-* and in the mammary gland parenchyma of *E. coli*-infected cow (18, 66) or in the bovine mammary epithelial cells (9). But the expression level of *NFKBIA* did not significantly alter in blood monocyte-derived macrophages infected with *S. agalactiae* (67), blood leukocytes infected with *Staphylococcus aureus* (68) and *E. coli* (69), circulating leukocytes with clinical or subclinical mastitis in the UK (25), and the Chinese Holstein mammary fibroblasts infected with *S. aureus* (70). Meanwhile, the expression level of *CXCL8, CCL5, IL6, CXCL3, IL1A, NOD2, TNF, CCL2, IL17A, IL10*, and *CCL20* were significantly upregulated and showed a strong inflammatory response in these pathological tissues and cells, especially in monocyte-derived macrophages (67).

In conclusion, *NFKBIA* is the hub gene to regulate the expression of pro-inflammatory cytokines, chemokines, anti-apoptotic genes, anti-microbial genes, and inflammatory genes by controlling the NF-κB pathway to maintain a mild inflammatory response after infection in bovine mammary epithelial cells and mammary gland parenchyma (Figure 9B).

### The Top-Expressed Differentially Expressed miRNAs

Among the 29 top expressed miRNAs (Figure 4), *bta-miR-30a-5p* is differentially upregulated at 4 h and 12 h; *bta-miR-125b and bta-miR-100* are differentially downregulated at 4 and 8 h, respectively (Supplementary Tables 3, 4). Luoreng et al. (71) verified that *bta-miR-125b* targets and inhibits *NKIRAS2* gene expression, decreased *miR-125b* in LPS-induced MAC-T cells leading to *NKIRAS2* increase, then inhibits NF-κB activity, leading to a low expression of the inflammatory factors, IL6 and TNFα.

The TNFAIP3 protein (A20) has emerged as an important negative regulator of TLR and retinoic acid-inducible gene 1 (RIG-I) signaling, restricts and terminates the ubiquitination status of central components in NF-κB, IRF3, and apoptosis signaling cascades, resulting in the downregulation of interferon (IFN1/2) production during inflammatory response (72). This conclusion was in line with our results; the relatively low and decreased *IFN*β*1/2* (Figure 9A) may be regulated by significantly increased *TNFAIP3* in LPS-induced bMECs (Figure 2E), to restrict and terminate inflammatory responses.

The *MiR-30a* was related to the stress response of Holstein cattle (73) and proved to target the 3′UTR of *GRP78* to regulate the endoplasmic reticulum (ER) stress, then controlling the autophagy (74). Significantly upregulated *miR-30a* and downregulated *GRP78* axis may control the ER stress response in LPS-challenged bMECs (Figure 6A). Overexpression of *miR-100* inhibited matrix metalloproteinase-9 (*MMP9*) expression in H_2_O_2_-induced human umbilical vein endothelial cells, limited cell inflammation, oxidative stress, and cell apoptosis (75). However, significantly downregulated *miR-100* and upregulated *MMP9* (Figure 9A) will facilitate the activity of cell inflammation, oxidative stress, and cell apoptosis in LPS-induced bMECs.

### The Milk Synthesis Ability of bMECs After LPS Induction

The mammary gland is a specialized organ providing nutrition for mammalian offspring and commences during embryogenesis with the formation of an epithelial thickening known as placode (76). Mammary epithelial cells are the main constituent cells of epithelial, which is the secretory part of the mammary gland. Mastitis directly impacts the production performance of MECs. In the present study, the mammary development specification of bMECs may get changed after LPS treatment due to significantly downregulated *SOSTDC1, WNT7B, MSX1*, and *MSX2*, which are involved in the mammary gland bud morphogenesis and mammary gland epithelium development (Figures 8A–C), which will affect the normal development of mammary epithelial cells and affect their lactation function.

Furthermore, upregulated chitinase 3 like protein 1 (*CHI3L1*) should play a critical role in the cytokine signaling in LPS-induced bMECs; the function was proved during *E. coli*-triggered inflammation of the mammary gland (77). *Bta-miR-2425-5p* was predicted to target the 3′UTR of *CHI3L1* and significantly downregulated after LPS induction (Figure 6A). The *Bta-miR-2425-5p* can inhibit triglyceride synthesis by regulating *ADAMTS1*, which is involved in milk fat metabolism within the lactation system. The *bta-miR-2425-5p* is significantly downregulated in low-fat milk percentage bMECs (78). The expression level of *ADAMTS1* has also dropped in this study (data not shown). Altogether, these results reflect the undermined synthesis ability of milk fat in LPS-induced bMECs.

## Data Availability Statement

The datasets presented in this study can be found in online repositories. The names of the repository/repositories and accession number(s) can be found below: https://www.ncbi.nlm.nih.gov/, SRX6610779; https://www.ncbi.nlm.nih.gov/, SRX6610780; https://www.ncbi.nlm.nih.gov/, SRX6610781; https://www.ncbi.nlm.nih.gov/, SRX6610782; https://www.ncbi.nlm.nih.gov/, SRX6610783; https://www.ncbi.nlm.nih.gov/, SRX6610784; https://www.ncbi.nlm.nih.gov/, SRX6610785; https://www.ncbi.nlm.nih.gov/, SRX6610786.

## Ethics Statement

Ethical review and approval was not required for the animal study because this study didn't use any animals.

## Author Contributions

LC performed the experiments, analyzed the data, and prepared the draft of the manuscript. ZL and RT performed the experiments. XL contributed to the conception and design of the study. XL, HZ, and JW contributed to the manuscript revision. All authors read and approved the final manuscript.

## Funding

We would like to thank the 13115 Sci-Tech Innovation Program of Shaanxi Province (No. 2015KTCL02-11), the High-level Talents Start-up Project Fund of Ankang University (2020AYQDZR09), and the Shaanxi Provincial Department of Education Project (20JK0477) for funding this research.

## Conflict of Interest

The authors declare that the research was conducted in the absence of any commercial or financial relationships that could be construed as a potential conflict of interest.

## Publisher's Note

All claims expressed in this article are solely those of the authors and do not necessarily represent those of their affiliated organizations, or those of the publisher, the editors and the reviewers. Any product that may be evaluated in this article, or claim that may be made by its manufacturer, is not guaranteed or endorsed by the publisher.

## Supplementary Material

Supplementary Figure 1 The relative differential relationship of each gene between different timepoints, such as log_2_(12/0 h), log_2_(8/0 h), log_2_(4/0 h), log_2_(12/8 h), log_2_(12/4 h), log_2_(8/4 h),values. Dark green represents downregulation and red represents upregulation.

Supplementary Figure 2 The relative differential relationship of each miRNA between different timepoints, such as log_2_(12/0 h), log_2_(8/0 h), log_2_(4/0 h), log_2_(12/8 h), log_2_(12/4 h), and log_2_(8/4 h) values. Dark green represents downregulation and red represents upregulation.

Supplementary Table 1 The primers of mRNA and microRNA for QT-realtime PCR.

Supplementary Table 2 The differentially expressed genes (DEGs) in LPS-induced 4, 8, and 12 h bMECs. **(A)** DEGs in LPS-induced 4 h bMECs. **(B)** DEGs in LPS-induced 8 h bMECs. **(C)** DEGs in LPS-induced 12 h bMECs.

Supplementary Table 3 The expression level of miRNA in LPS-induced bMEC at 0, 4, 8, and 12 h.

Supplementary Table 4 The differentially expressed miRNAs (DEMs) in LPS-induced 4, 8, and 12 h bMECs. **(A)** DEMs in LPS-induced 4 h bMECs. **(B)** DEMs in LPS-induced 8 h bMECs. **(C)** DEMs in LPS-induced 12 h bMECs.
