# Supplementary figures and images for: Integrated Analysis of Transcriptome mRNA and miRNA Profiles Reveals Self-Protective Mechanism of Bovine MECs Induced by LPS
Source: Front Vet Sci. 2022 Jun 23;9:890043. doi: 10.3389/fvets.2022.890043 (PMC9260119; doi:10.3389/fvets.2022.890043)

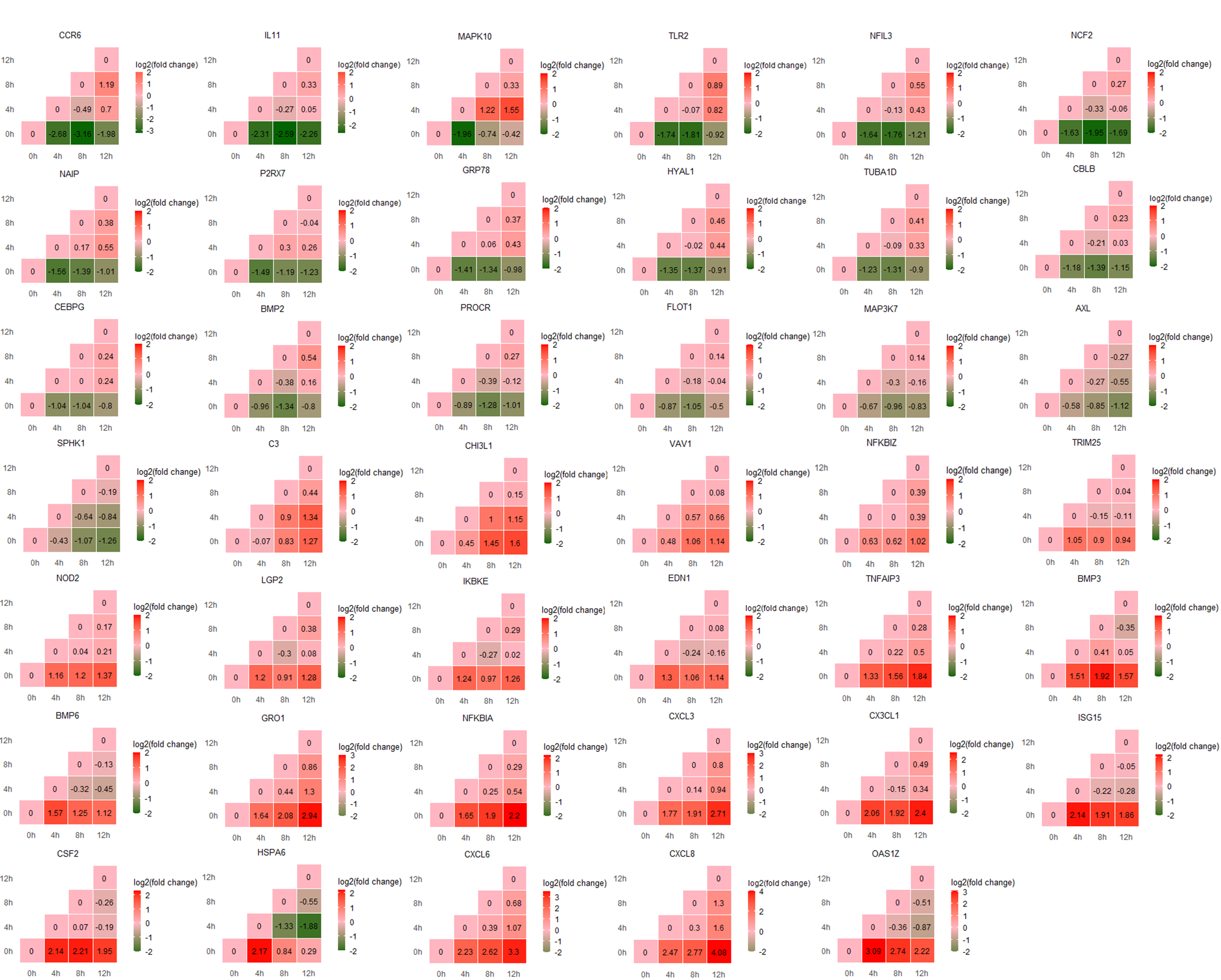

Supplement: Supplementary Figure 1 — The relative differential relationship of each gene between different timepoints, such as log2(12/0 h), log2(8/0 h), log2(4/0 h), log2(12/8 h), log2(12/4 h), log2(8/4 h),values. Dark green represents downregulation and red represents upregulation. [file Image_1.PNG]

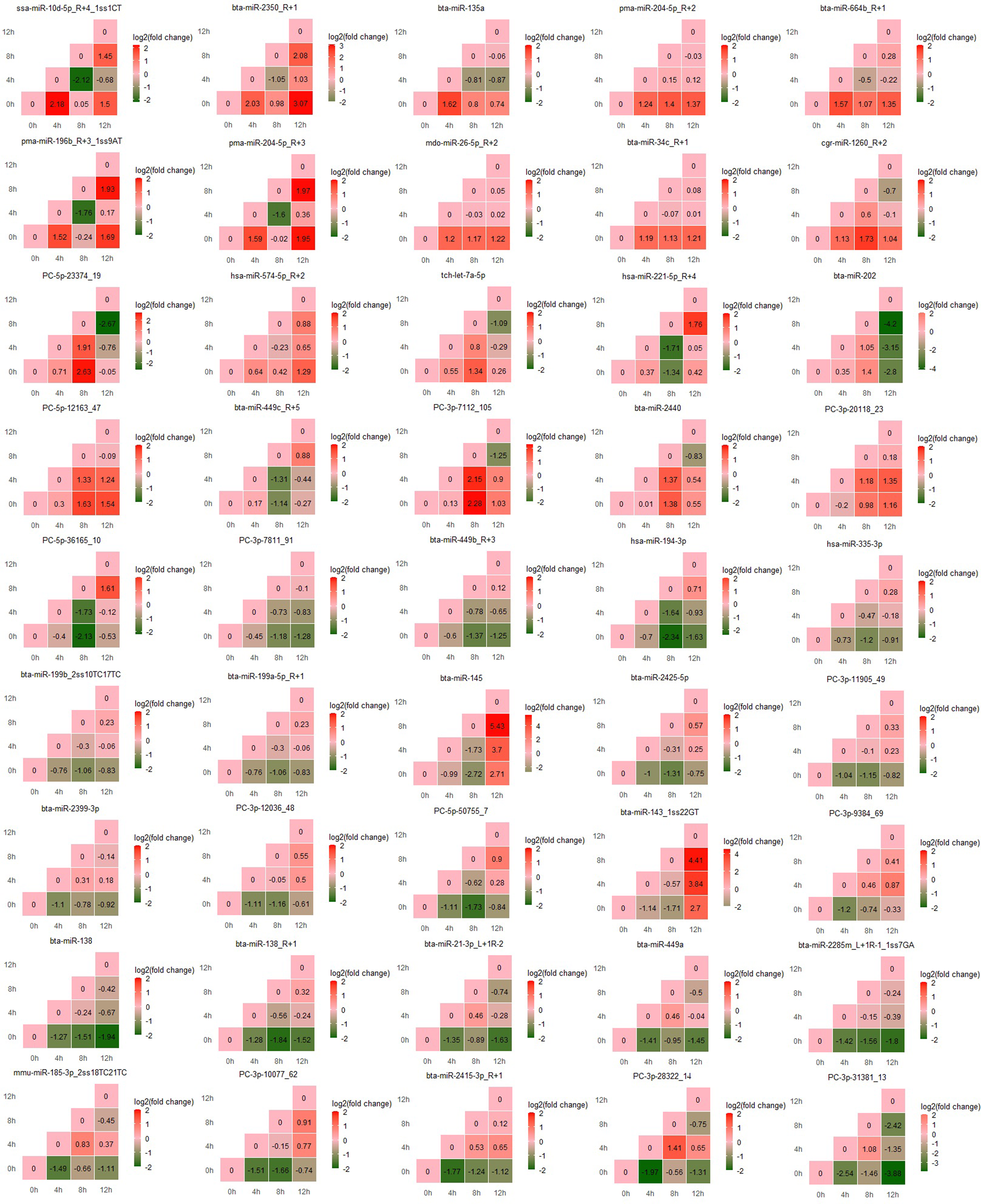

Supplement: Supplementary Figure 2 — The relative differential relationship of each miRNA between different timepoints, such as log2(12/0 h), log2(8/0 h), log2(4/0 h), log2(12/8 h), log2(12/4 h), and log2(8/4 h) values. Dark green represents downregulation and red represents upregulation. [file Image_2.PNG]
