# Supplementary material for: Integrated Analysis of Transcriptome mRNA and miRNA Profiles Reveals Self-Protective Mechanism of Bovine MECs Induced by LPS
Source: Front Vet Sci. 2022 Jun 23;9:890043. doi: 10.3389/fvets.2022.890043 (PMC9260119; doi:10.3389/fvets.2022.890043)
Supplement: Supplementary Table 1 — The primers of mRNA and microRNA for QT-realtime PCR. [file Table_1.DOC]

**Supplementary Table 1**: The primers of of mRNA and microRNA for QT-Realtime PCR

| symbol | Primer | Sequence (5’-3’) |
| --- | --- | --- |
| bta-miR-664b_R+1 | RT primer | GTCGTATCCAGTGCAGGGTCCGAGGTATTCGCACTGGATACGAC tgtaggct |
|  | F primer | CTGGAG TATTCATTTATCTCCC |
| bta-miR-21-3p_L+1R-2 | RT primer | GTCGTATCCAGTGCAGGGTCCGAGGTATTCGCACTGGATACGAC acagccca |
|  | F primer | CTGGAG CAACAGCAGTCGA |
| bta-miR-2313-3p_R+1 | RT primer | GTCGTATCCAGTGCAGGGTCCGAGGTATTCGCACTGGATACGAC aggcatgc |
|  | F primer | CTGGAG CCAGTTCCACGCT |
| bta-miR-2285j_L-1R+1 | RT primer | GTCGTATCCAGTGCAGGGTCCGAGGTATTCGCACTGGATACGAC caaaaagt |
|  | F primer | CTGGAG AAAACCAGAACGA |
| bta-miR-199a-5p_R+1 | RT primer | GTCGTATCCAGTGCAGGGTCCGAGGTATTCGCACTGGATACGAC gaacaggt |
|  | F primer | CTGGAG CCCAGTGTTCAGACT |
| bta-miR-339b_R+1 | RT primer | GTCGTATCCAGTGCAGGGTCCGAGGTATTCGCACTGGATACGAC tgagctcc |
|  | F primer | CTGGAG TCCCTGTCCTCCA |
| bta-miR-429 | RT primer | GTCGTATCCAGTGCAGGGTCCGAGGTATTCGCACTGGATACGAC acggcatt |
|  | F primer | CTGGAG TAATACTGTCTGGT |
| bta-miR-200b_R+2 | RT primer | GTCGTATCCAGTGCAGGGTCCGAGGTATTCGCACTGGATACGAC gtcatcat |
|  | F primer | CTGGAG TAATACTGCCTGGTA |
| bta-miR-135a | RT primer | GTCGTATCCAGTGCAGGGTCCGAGGTATTCGCACTGGATACGAC tcacatag |
|  | F primer | CTGGAG TATGGCTTTTTATTC |
| bta-miR-15a_R+1 | RT primer | GTCGTATCCAGTGCAGGGTCCGAGGTATTCGCACTGGATACGAC cacaaacc |
|  | F primer | CTGGAG TAGCAGCACATAAT |
| bta-miR-138_R+1 | RT primer | GTCGTATCCAGTGCAGGGTCCGAGGTATTCGCACTGGATACGAC acggcctg |
|  | F primer | CTGGAG AGCTGGTGTTGTGAAT |
| bta-miR-449a | RT primer | GTCGTATCCAGTGCAGGGTCCGAGGTATTCGCACTGGATACGAC accagcta |
|  | F primer | CTGGAG TGGCAGTGTATTGT |
| miRNA通用反向引物 | R Primer | GTGCAGGGTCCGAGGT |
| CTH | F primer | CTTCAGGCAGGTGGCA |
|  | R primer | CGCAGGCTTCAATGTC |
| IRF3 | F primer | CGTTTCCGCATCCCTT |
|  | R primer | GCTGTGGTCCTCCGCTA |
| KLF4 | F primer | CCACCTCGCCTTACAC |
|  | R primer | TTGTTGGGAACTTGACC |
| MAP3K7 | F primer | TCTGAAGGCAAGAGGAT |
|  | R primer | TTCTGACGCTAGGACTG |
| MX1 | F primer | AAATCGGTATCGTGGCA |
|  | R primer | CAGTTCGGTGGAGGTTG |
| TLR2 | F primer | TCCTGGCAAGTGGATTA |
|  | R primer | ACGGAAATGGGAGAAGT |
| TRIM25 | F primer | GGGACGAGTTTGAGTTTCT |
|  | R primer | TGCCCTGATAGATTTCCTTA |
| ISG15 | F primer | CAATGTGCCTGCTTTCC |
|  | R primer | GACCCTTGTCGTTCCTCA |
| TNFAIP3 | F primer | TGAACTTGTCCAGCACG |
|  | R primer | GAAGAATGGGCAGTTAGG |
| NOD1 | F primer | AACTCGCAGACGCTTATGT |
|  | R primer | TTTGGCTGTATCCGCTCA |
| IL18RAP | F primer | CGAGCTTCTGAATACCTG |
|  | R primer | TTCTCCTGGATGACCTGA |
| NFKBIA | F primer | AACCAGCCAGAAATCG |
|  | R primer | CCAGGTAGCCATGAATAG |
| β-actin | F primer | AACCGTGAGAAGATGACCCAGAT |
|  | R primer | CAGAGGCATACAGGGACAGCA |
