# Supplementary material for: Integrated Analysis of Transcriptome mRNA and miRNA Profiles Reveals Self-Protective Mechanism of Bovine MECs Induced by LPS
Source: Front Vet Sci. 2022 Jun 23;9:890043. doi: 10.3389/fvets.2022.890043 (PMC9260119; doi:10.3389/fvets.2022.890043)
Supplement: Supplementary Table 3 — The expression level of miRNA in LPS-induced bMEC at 0, 4, 8, and 12 h. [file Table_3.DOC]

**Supplementary Table 3: the expression level of miRNA in LPS induced bMEC at 0, 4, 8 and 12h.**

| ID | miR_name | miR_seq | Sequence in miRbase | bMEC_Lps_0h(norm) copies | % | bMEC_Lps_4h(norm) copies | % | bMEC_Lps_8h(norm) copies | % | bMEC_Lps_12h(norm) copies | % |
| --- | --- | --- | --- | --- | --- | --- | --- | --- | --- | --- | --- |
| 1 | bta-miR-21-5p_R-1 | TAGCTTATCAGACTGATGTTGAC | Diff | 2034983.67 | 0.31 | 2290327.60 | 0.31 | 1964206.56 | 0.27 | 2350517.19 | 0.31 |
| 2 | bta-let-7a-5p | TGAGGTAGTAGGTTGTATAGTT | Yes | 352430.46 | 0.05 | 498944.52 | 0.07 | 599062.80 | 0.08 | 504974.40 | 0.07 |
| 3 | bta-miR-27a-3p_R+1 | TTCACAGTGGCTAAGTTCCGC | Diff | 250900.20 | 0.04 | 192531.42 | 0.03 | 248314.73 | 0.03 | 175395.14 | 0.02 |
| 4 | bta-miR-27b | TTCACAGTGGCTAAGTTCTGC | Yes | 222455.25 | 0.03 | 257835.23 | 0.03 | 321565.11 | 0.04 | 302086.84 | 0.04 |
| 5 | bta-miR-26a | TTCAAGTAATCCAGGATAGGCT | Yes | 208398.82 | 0.03 | 284546.26 | 0.04 | 339080.80 | 0.05 | 321241.24 | 0.04 |
| 6 | bta-miR-10a_R-1 | TACCCTGTAGATCCGAATTTGT | Diff | 204796.48 | 0.03 | 237199.66 | 0.03 | 134137.26 | 0.02 | 245755.06 | 0.03 |
| 7 | bta-miR-24-3p_R+1 | TGGCTCAGTTCAGCAGGAACAGT | Diff | 155052.06 | 0.02 | 149870.64 | 0.02 | 166454.18 | 0.02 | 150631.46 | 0.02 |
| 8 | bta-let-7i | TGAGGTAGTAGTTTGTGCTGTT | Yes | 149527.63 | 0.02 | 130655.13 | 0.02 | 143256.58 | 0.02 | 135162.32 | 0.02 |
| 9 | bta-miR-29a_L-1 | TAGCACCATCTGAAATCGGTTA | Diff | 141252.33 | 0.02 | 127715.92 | 0.02 | 182438.70 | 0.03 | 125498.13 | 0.02 |
| 10 | bta-let-7f | TGAGGTAGTAGATTGTATAGTT | Yes | 138946.22 | 0.02 | 196218.28 | 0.03 | 227590.96 | 0.03 | 223967.16 | 0.03 |
| 11 | bta-let-7b | TGAGGTAGTAGGTTGTGTGGTT | Yes | 130623.68 | 0.02 | 167621.68 | 0.02 | 195835.74 | 0.03 | 159791.97 | 0.02 |
| 12 | bta-miR-10b_R-1 | TACCCTGTAGAACCGAATTTGT | Diff | 126687.48 | 0.02 | 220381.99 | 0.03 | 100387.19 | 0.01 | 213418.63 | 0.03 |
| 13 | bta-miR-30b-5p | TGTAAACATCCTACACTCAGCT | Yes | 125751.75 | 0.02 | 165298.82 | 0.02 | 195699.25 | 0.03 | 125234.73 | 0.02 |
| 14 | bta-miR-30a-5p | TGTAAACATCCTCGACTGGAAGCT | Yes | 109470.61 | 0.02 | 233202.07 | 0.03 | 199750.21 | 0.03 | 246781.85 | 0.03 |
| 15 | bta-miR-100 | AACCCGTAGATCCGAACTTGTG | Yes | 108306.82 | 0.02 | 60264.85 | 0.01 | 44407.60 | 0.01 | 54369.41 | 0.01 |
| 16 | bta-miR-16a | TAGCAGCACGTAAATATTGGTG | Yes | 95570.63 | 0.01 | 102314.16 | 0.01 | 96149.45 | 0.01 | 72920.81 | 0.01 |
| 17 | bta-miR-221 | AGCTACATTGTCTGCTGGGTTT | Yes | 95082.59 | 0.01 | 74676.97 | 0.01 | 75863.95 | 0.01 | 81833.12 | 0.01 |
| 18 | bta-miR-191_R-1 | CAACGGAATCCCAAAAGCAGCT | Diff | 86197.14 | 0.01 | 68308.10 | 0.01 | 52240.95 | 0.01 | 77055.66 | 0.01 |
| 19 | bta-miR-23a_R-1 | ATCACATTGCCAGGGATTTCC | Diff | 86131.65 | 0.01 | 101554.69 | 0.01 | 93248.86 | 0.01 | 83964.89 | 0.01 |
| 20 | bta-miR-99b | CACCCGTAGAACCGACCTTGCG | Yes | 85635.51 | 0.01 | 61022.42 | 0.01 | 49666.57 | 0.01 | 62847.27 | 0.01 |
| 21 | bta-miR-30d | TGTAAACATCCCCGACTGGAAGCT | Yes | 77101.66 | 0.01 | 138279.47 | 0.02 | 106684.53 | 0.01 | 143508.93 | 0.02 |
| 22 | bta-miR-125b | TCCCTGAGACCCTAACTTGTGA | Yes | 71404.10 | 0.01 | 32727.86 | 0.00 | 36678.08 | 0.01 | 39954.54 | 0.01 |
| 23 | bta-miR-92a | TATTGCACTTGTCCCGGCCTGT | Yes | 57381.08 | 0.01 | 60651.96 | 0.01 | 61222.88 | 0.01 | 51165.04 | 0.01 |
| 24 | bta-let-7g | TGAGGTAGTAGTTTGTACAGTT | Yes | 48304.92 | 0.01 | 63993.03 | 0.01 | 67833.94 | 0.01 | 64095.69 | 0.01 |
| 25 | bta-miR-222_R+2 | AGCTACATCTGGCTACTGGGTCT | Diff | 48220.27 | 0.01 | 33208.00 | 0.00 | 25800.64 | 0.00 | 36790.45 | 0.00 |
| 26 | bta-miR-26b | TTCAAGTAATTCAGGATAGGTT | Yes | 40587.25 | 0.01 | 48368.89 | 0.01 | 58982.54 | 0.01 | 46719.54 | 0.01 |
| 27 | bta-miR-23b-3p_R-2 | ATCACATTGCCAGGGATTACC | Diff | 36816.89 | 0.01 | 52235.82 | 0.01 | 59931.77 | 0.01 | 45492.04 | 0.01 |
| 28 | bta-miR-30f | TGTAAACACCCTACACTCTCAGCT | Yes | 36122.29 | 0.01 | 71123.64 | 0.01 | 50823.64 | 0.01 | 70986.13 | 0.01 |
| 29 | bta-miR-30e-5p | TGTAAACATCCTTGACTGGAAGCT | Yes | 36041.74 | 0.01 | 43884.94 | 0.01 | 38422.75 | 0.01 | 45645.02 | 0.01 |
| 30 | bta-miR-20a | TAAAGTGCTTATAGTGCAGGTAG | Yes | 27747.37 | 0.00 | 32839.98 | 0.00 | 27330.77 | 0.00 | 31598.78 | 0.00 |
| 31 | bta-miR-25 | CATTGCACTTGTCTCGGTCTGA | Yes | 27290.92 | 0.00 | 28480.60 | 0.00 | 29024.98 | 0.00 | 25660.50 | 0.00 |
| 32 | bta-miR-186_R+1 | CAAAGAATTCTCCTTTTGGGCTT | Diff | 26717.37 | 0.00 | 38061.33 | 0.01 | 23869.85 | 0.00 | 34335.88 | 0.00 |
| 33 | bta-miR-16b_R+1 | TAGCAGCACGTAAATATTGGCG | Diff | 26062.79 | 0.00 | 20309.67 | 0.00 | 22081.11 | 0.00 | 16436.26 | 0.00 |
| 34 | bta-miR-148b | TCAGTGCATCACAGAACTTTGT | Yes | 25857.55 | 0.00 | 23731.26 | 0.00 | 20395.22 | 0.00 | 21857.83 | 0.00 |
| 35 | bta-miR-151-5p | TCGAGGAGCTCACAGTCTAGT | Yes | 25689.94 | 0.00 | 24985.48 | 0.00 | 34417.18 | 0.00 | 32566.76 | 0.00 |
| 36 | eca-miR-151-5p | TCGAGGAGCTCACAGTCTAGT | Yes | 25689.94 | 0.00 | 24985.48 | 0.00 | 34417.18 | 0.00 | 32566.76 | 0.00 |
| 37 | bta-miR-9-5p_R+1 | TCTTTGGTTATCTAGCTGTATGA | Diff | 25389.70 | 0.00 | 23537.90 | 0.00 | 21482.58 | 0.00 | 22022.12 | 0.00 |
| 38 | bta-miR-31_R+2 | AGGCAAGATGCTGGCATAGCTGT | Diff | 21214.14 | 0.00 | 19081.85 | 0.00 | 31116.91 | 0.00 | 27998.74 | 0.00 |
| 39 | bta-miR-34a | TGGCAGTGTCTTAGCTGGTTGT | Yes | 20817.22 | 0.00 | 27123.06 | 0.00 | 27716.73 | 0.00 | 21441.71 | 0.00 |
| 40 | bta-miR-6119-5p | AGAGGTAAAAAATTGATTTGACT | Yes | 20639.60 | 0.00 | 28115.83 | 0.00 | 15284.66 | 0.00 | 29526.45 | 0.00 |
| 41 | bta-miR-21-3p_L+1R-2 | CAACAGCAGTCGATGGGCTGT | Diff | 19598.69 | 0.00 | 7682.13 | 0.00 | 10564.96 | 0.00 | 6335.86 | 0.00 |
| 42 | bta-miR-96 | TTTGGCACTAGCACATTTTTGCT | Yes | 19594.72 | 0.00 | 27205.12 | 0.00 | 14864.41 | 0.00 | 25730.82 | 0.00 |
| 43 | bta-miR-17-5p_R-1 | CAAAGTGCTTACAGTGCAGGTAG | Diff | 18702.66 | 0.00 | 21611.28 | 0.00 | 16787.85 | 0.00 | 16507.63 | 0.00 |
| 44 | bta-miR-106a_R+1_1ss1AC | CAAAGTGCTTACAGTGCAGGTAG | Diff | 18702.66 | 0.00 | 21611.28 | 0.00 | 16787.85 | 0.00 | 16507.63 | 0.00 |
| 45 | bta-miR-22-3p_R+1 | AAGCTGCCAGTTGAAGAACTGT | Diff | 18640.14 | 0.00 | 24599.72 | 0.00 | 26090.60 | 0.00 | 22141.01 | 0.00 |
| 46 | bta-miR-106b | TAAAGTGCTGACAGTGCAGAT | Yes | 18362.30 | 0.00 | 18290.97 | 0.00 | 24024.14 | 0.00 | 13061.65 | 0.00 |
| 47 | bta-miR-148a | TCAGTGCACTACAGAACTTTGT | Yes | 17937.11 | 0.00 | 12283.61 | 0.00 | 13570.36 | 0.00 | 13614.56 | 0.00 |
| 48 | bta-miR-193b_R-2 | AACTGGCCCACAAAGTCCCGCT | Diff | 17806.12 | 0.00 | 14425.12 | 0.00 | 13737.87 | 0.00 | 12340.62 | 0.00 |
| 49 | bta-miR-342_R-1 | TCTCACACAGAAATCGCACCCATC | Diff | 17746.09 | 0.00 | 22932.66 | 0.00 | 15565.80 | 0.00 | 22597.40 | 0.00 |
| 50 | bta-miR-15a_R+1 | TAGCAGCACATAATGGTTTGTG | Diff | 17222.16 | 0.00 | 20497.87 | 0.00 | 20508.36 | 0.00 | 15089.22 | 0.00 |
| 51 | xtr-miR-92b_R+1 | TATTGCACTCGTCCCGGCCTCC | Diff | 17125.91 | 0.00 | 18561.90 | 0.00 | 20309.01 | 0.00 | 19187.21 | 0.00 |
| 52 | bta-miR-339b_R+1 | TCCCTGTCCTCCAGGAGCTCA | Diff | 17101.10 | 0.00 | 17142.63 | 0.00 | 25701.21 | 0.00 | 16680.22 | 0.00 |
| 53 | bta-miR-365-3p | TAATGCCCCTAAAAATCCTTAT | Yes | 16685.34 | 0.00 | 13629.78 | 0.00 | 6148.44*2 | #VALUE! | 11369.02 | 0.00 |
| 54 | mdo-miR-26-5p_R+2 | TTCAAGTAATCCAGGATAGGCTT | Diff | 15420.16 | 0.00 | 35461.62 | 0.00 | 34714.00 | 0.00 | 35852.10 | 0.00 |
| 55 | bta-miR-15b | TAGCAGCACATCATGGTTTACA | Yes | 14960.74 | 0.00 | 10917.95 | 0.00 | 11675.33 | 0.00 | 12921.02 | 0.00 |
| 56 | bta-miR-125a_R+1 | TCCCTGAGACCCTTTAACCTGTGA | Diff | 13847.39 | 0.00 | 11606.06 | 0.00 | 10580.63 | 0.00 | 20121.10 | 0.00 |
| 57 | bta-miR-93_R+1 | CAAAGTGCTGTTCGTGCAGGTAG | Diff | 13762.05 | 0.00 | 18040.34 | 0.00 | 14155.18 | 0.00 | 15968.78 | 0.00 |
| 58 | bta-miR-378 | ACTGGACTTGGAGTCAGAAGGC | Yes | 13321.97 | 0.00 | 8671.65 | 0.00 | 7791.23 | 0.00 | 8697.11 | 0.00 |
| 59 | bta-miR-30c_R+1 | TGTAAACATCCTACACTCTCAGCT | Diff | 13261.94 | 0.00 | 23312.60 | 0.00 | 15484.17 | 0.00 | 20377.21 | 0.00 |
| 60 | bta-let-7d | AGAGGTAGTAGGTTGCATAGTT | Yes | 12538.06 | 0.00 | 14579.75 | 0.00 | 18226.96 | 0.00 | 18838.95 | 0.00 |
| 61 | bta-miR-181a_R-1 | AACATTCAACGCTGTCGGTGAGT | Diff | 11900.52 | 0.00 | 15187.16 | 0.00 | 11439.74 | 0.00 | 13373.58 | 0.00 |
| 62 | bta-miR-103 | AGCAGCATTGTACAGGGCTATGA | Yes | 11890.26 | 0.00 | 11423.94 | 0.00 | 13119.42 | 0.00 | 12498.28 | 0.00 |
| 63 | bta-miR-9-3p_R+5 | ATAAAGCTAGATAACCGAAAGT | Diff | 11411.32 | 0.00 | 8444.98 | 0.00 | 7952.38 | 0.00 | 6412.56 | 0.00 |
| 64 | bta-miR-29b | TAGCACCATTTGAAATCAGTGTT | Yes | 10613.26 | 0.00 | 13207.42 | 0.00 | 12779.78 | 0.00 | 7907.98 | 0.00 |
| 65 | hsa-miR-340-5p | TTATAAAGCAATGAGACTGATT | Yes | 10342.63 | 0.00 | 8451.48 | 0.00 | 10124.14 | 0.00 | 7639.85 | 0.00 |
| 66 | bta-miR-224_R-1 | CAAGTCACTAGTGGTTCCGTTT | Diff | 10279.12 | 0.00 | 22367.22 | 0.00 | 17815.94 | 0.00 | 25082.66 | 0.00 |
| 67 | bta-miR-130a_R+1 | CAGTGCAATGTTAAAAGGGCATT | Diff | 9169.74 | 0.00 | 10378.92 | 0.00 | 10046.75 | 0.00 | 6659.30 | 0.00 |
| 68 | bta-miR-450a | TTTTGCGATGTGTTCCTAATAT | Yes | 8813.50 | 0.00 | 7876.30 | 0.00 | 10042.84 | 0.00 | 7035.16 | 0.00 |
| 69 | bta-miR-101_L+1R-1 | GTACAGTACTGTGATAACTGA | Diff | 8565.48 | 0.00 | 11749.86 | 0.00 | 15794.04 | 0.00 | 9590.72 | 0.00 |
| 70 | bta-miR-423-3p_L-1 | AGCTCGGTCTGAGGCCCCTCAGT | Diff | 8123.87 | 0.00 | 10402.08 | 0.00 | 9268.95 | 0.00 | 9944.85 | 0.00 |
| 71 | bta-miR-3431 | CCTCAGTCAGCCTTGTGGATGT | Yes | 8008.76 | 0.00 | 14353.63 | 0.00 | 15461.97 | 0.00 | 15013.80 | 0.00 |
| 72 | bta-miR-182 | TTTGGCAATGGTAGAACTCACACT | Yes | 7832.13 | 0.00 | 16636.50 | 0.00 | 12379.17 | 0.00 | 17878.74 | 0.00 |
| 73 | hsa-miR-1307-5p | TCGACCGGACCTCGACCGGCT | Yes | 7784.50 | 0.00 | 6289.66 | 0.00 | 9196.46 | 0.00 | 4607.44 | 0.00 |
| 74 | bta-miR-183_R-1 | TATGGCACTGGTAGAATTCACT | Diff | 7663.45 | 0.00 | 8124.90 | 0.00 | 6204.77 | 0.00 | 8057.90 | 0.00 |
| 75 | bta-miR-98 | TGAGGTAGTAAGTTGTATTGTT | Yes | 7581.25 | 0.00 | 9108.38 | 0.00 | 11387.69 | 0.00 | 9387.46 | 0.00 |
| 76 | bta-miR-19b | TGTGCAAATCCATGCAAAACTGA | Yes | 7541.72 | 0.00 | 37402.74 | 0.00 | 4186.74 | 0.00 | 21995.26 | 0.00 |
| 77 | ola-miR-92a_R+3 | TATTGCACTTGTCCCGGCCTGTAT | Diff | 6714.32 | 0.00 | 8377.55 | 0.00 | 4547.29 | 0.00 | 8307.83 | 0.00 |
| 78 | hsa-miR-28-3p_1ss11TA | CACTAGATTGAGAGCTCCTGGA | Diff | 6660.74 | 0.00 | 6645.50 | 0.00 | 6430.57 | 0.00 | 7354.77 | 0.00 |
| 79 | bta-miR-424-5p_R+2 | CAGCAGCAATTCATGTTTTGAAT | Diff | 6468.73 | 0.00 | 8172.01 | 0.00 | 8265.84 | 0.00 | 7802.21 | 0.00 |
| 80 | hsa-miR-221-5p_R+4 | ACCTGGCATACAATGTAGATTTCTGT | Diff | 6115.48 | 0.00 | 7884.42 | 0.00 | 2411.77 | 0.00 | 8174.24 | 0.00 |
| 81 | bta-miR-484 | TCAGGCTCAGTCCCCTCCCGAT | Yes | 6104.56 | 0.00 | 4515.37 | 0.00 | 5473.99 | 0.00 | 4277.60 | 0.00 |
| 82 | bta-miR-196b_R-1 | TAGGTAGTTTCCTGTTGTTGGG | Diff | 5990.94 | 0.00 | 10102.84 | 0.00 | 5881.01 | 0.00 | 7064.56 | 0.00 |
| 83 | bta-miR-107_R+1_1ss22CG | AGCAGCATTGTACAGGGCTATGA | Diff | 5945.13 | 0.00 | 5711.91 | 0.00 | 6559.71 | 0.00 | 6249.14 | 0.00 |
| 84 | bta-miR-452_R-1 | TGTTTGCAGAGGAAACTGAGA | Diff | 5760.24 | 0.00 | 10740.04 | 0.00 | 9270.91 | 0.00 | 11329.38 | 0.00 |
| 85 | bta-miR-425-5p_L+1R+2 | AATGACACGATCACTCCCGTTGAGT | Diff | 5744.36 | 0.00 | 7055.77 | 0.00 | 5080.19 | 0.00 | 5338.69 | 0.00 |
| 86 | bta-miR-449a | TGGCAGTGTATTGTTAGCTGGT | Yes | 5546.89 | 0.00 | 2083.83 | 0.00 | 2874.14 | 0.00 | 2029.50 | 0.00 |
| 87 | bta-miR-140_L-1R+1 | ACCACAGGGTAGAACCACGGAC | Diff | 5430.80 | 0.00 | 3578.66 | 0.00 | 3592.18 | 0.00 | 3565.52 | 0.00 |
| 88 | bta-miR-152_R-1 | TCAGTGCATGACAGAACTTGG | Diff | 5366.30 | 0.00 | 4970.31 | 0.00 | 6772.94 | 0.00 | 5099.63 | 0.00 |
| 89 | chi-miR-1249 | ACGCCCTTCCCCCCCTTCTTCA | Yes | 5259.13 | 0.00 | 7784.50 | 0.00 | 8227.64 | 0.00 | 6996.81 | 0.00 |
| 90 | bta-miR-200b_R+2 | TAATACTGCCTGGTAATGATGAC | Diff | 5103.34 | 0.00 | 15216.41 | 0.00 | 14599.92 | 0.00 | 16536.40 | 0.00 |
| 91 | bta-miR-2284x_R+1 | TGAAAAGTTCGTTCGGGTTTTT | Diff | 4826.51 | 0.00 | 5474.76 | 0.00 | 6414.68 | 0.00 | 5304.51 | 0.00 |
| 92 | bta-miR-155_R+1 | TTAATGCTAATCGTGATAGGGGTT | Diff | 4812.60 | 0.00 | 7137.82 | 0.00 | 4978.31 | 0.00 | 7449.37 | 0.00 |
| 93 | bta-let-7e_R+1 | TGAGGTAGGAGGTTGTATAGTT | Diff | 4686.58 | 0.00 | 5748.47 | 0.00 | 6930.98 | 0.00 | 6022.69 | 0.00 |
| 94 | bta-miR-423-5p | TGAGGGGCAGAGAGCGAGACTTT | Yes | 4667.73 | 0.00 | 5696.61 | 0.00 | 5396.60 | 0.00 | 6794.82 | 0.00 |
| 95 | bta-miR-151-3p | CTAGACTGAAGCTCCTTGAGG | Yes | 4489.11 | 0.00 | 4386.19 | 0.00 | 5127.21 | 0.00 | 4971.79 | 0.00 |
| 96 | efu-miR-151_L-2R-2 | CTAGACTGAAGCTCCTTGAGG | Diff | 4489.11 | 0.00 | 4386.19 | 0.00 | 5127.21 | 0.00 | 4971.79 | 0.00 |
| 97 | hsa-miR-31-3p_R+1 | TGCTATGCCAACATATTGCCATC | Diff | 4474.23 | 0.00 | 3913.37 | 0.00 | 5347.62 | 0.00 | 3266.37 | 0.00 |
| 98 | bta-miR-193a-3p | AACTGGCCTACAAAGTCCCAGT | Yes | 4219.71 | 0.00 | 5640.55 | 0.00 | 5420.11 | 0.00 | 2305.00 | 0.00 |
| 99 | bta-miR-331-3p_R+1 | GCCCCTGGGCCTATCCTAGAAT | Diff | 4202.34 | 0.00 | 2677.70 | 0.00 | 4293.58 | 0.00 | 4168.94 | 0.00 |
| 100 | bta-miR-320a_R+1 | AAAAGCTGGGTTGAGAGGGCGAT | Diff | 4178.52 | 0.00 | 5664.12 | 0.00 | 4704.02 | 0.00 | 6233.58 | 0.00 |
| 101 | bta-miR-1468 | CTCCGTTTGCCTGTTTTGCTGA | Yes | 3987.02 | 0.00 | 5892.40 | 0.00 | 4766.72 | 0.00 | 6306.46 | 0.00 |
| 102 | bta-miR-128 | TCACAGTGAACCGGTCTCTTT | Yes | 3972.13 | 0.00 | 4042.54 | 0.00 | 5948.12 | 0.00 | 5121.36 | 0.00 |
| 103 | bta-miR-769 | TGAGACCTCCGGGTTCTGAGCT | Yes | 3972.13 | 0.00 | 2030.21 | 0.00 | 2366.71 | 0.00 | 2160.53 | 0.00 |
| 104 | pma-miR-23b_R+1_1ss23CT | ATCACATTGCCAGGGATTACCATT | Diff | 3972.13 | 0.00 | 9208.65 | 0.00 | 4882.31 | 0.00 | 5745.23 | 0.00 |
| 105 | bta-miR-28 | AAGGAGCTCACAGTCTATTGAG | Yes | 3947.08 | 0.00 | 4806.07 | 0.00 | 4900.93 | 0.00 | 4571.00 | 0.00 |
| 106 | mml-miR-28-5p | AAGGAGCTCACAGTCTATTGAG | Yes | 3947.08 | 0.00 | 4806.07 | 0.00 | 4900.93 | 0.00 | 4571.00 | 0.00 |
| 107 | bta-miR-7 | TGGAAGACTAGTGATTTTGTTGTT | Yes | 3931.44 | 0.00 | 4741.20 | 0.00 | 2852.58 | 0.00 | 4478.31 | 0.00 |
| 108 | bta-miR-32_R-2 | TATTGCACATGACTAAGTTGC | Diff | 3895.73 | 0.00 | 3881.69 | 0.00 | 4848.03 | 0.00 | 3181.99 | 0.00 |
| 109 | dre-miR-27b-3p_R+1 | TTCACAGTGGCTAAGTTCTGCAT | Diff | 3874.39 | 0.00 | 6216.01 | 0.00 | 6550.08 | 0.00 | 6283.02 | 0.00 |
| 110 | pma-miR-27b-3p_R+2 | TTCACAGTGGCTAAGTTCTGCAT | Diff | 3874.39 | 0.00 | 6216.01 | 0.00 | 6550.08 | 0.00 | 6283.02 | 0.00 |
| 111 | bta-miR-18a_R+1 | TAAGGTGCATCTAGTGCAGATAG | Diff | 3859.51 | 0.00 | 3172.45 | 0.00 | 3187.61 | 0.00 | 2822.12 | 0.00 |
| 112 | bta-miR-210_L-1 | CTGTGCGTGTGACAGCGGCTGA | Diff | 3786.57 | 0.00 | 4685.97 | 0.00 | 5065.50 | 0.00 | 3271.48 | 0.00 |
| 113 | bta-miR-6119-3p_L-1_1ss23AT | CAAATCATTTTTTACTCTCCAT | Diff | 3578.19 | 0.00 | 3326.81 | 0.00 | 3489.33 | 0.00 | 2354.85 | 0.00 |
| 114 | bta-miR-1307_R+1 | ACTCGGCGTGGCGTCGGTCGTGG | Diff | 3480.95 | 0.00 | 3716.77 | 0.00 | 3718.55 | 0.00 | 3105.29 | 0.00 |
| 115 | bta-miR-185 | TGGAGAGAAAGGCAGTTCCTGA | Yes | 3461.10 | 0.00 | 4439.00 | 0.00 | 3802.80 | 0.00 | 4717.38 | 0.00 |
| 116 | hsa-miR-30a-3p | CTTTCAGTCGGATGTTTGCAGC | Yes | 3430.84 | 0.00 | 7000.93 | 0.00 | 5912.36 | 0.00 | 5999.00 | 0.00 |
| 117 | bta-miR-192_R-2 | CTGACCTATGAATTGACAGCC | Diff | 3424.88 | 0.00 | 3096.49 | 0.00 | 4021.25 | 0.00 | 4622.78 | 0.00 |
| 118 | dre-miR-24_R+2 | TGGCTCAGTTCAGCAGGAACAGTT | Diff | 3351.95 | 0.00 | 4939.44 | 0.00 | 3913.00 | 0.00 | 4469.37 | 0.00 |
| 119 | bta-miR-149-5p_R-3 | TCTGGCTCCGTGTCTTCACT | Diff | 3350.96 | 0.00 | 1970.90 | 0.00 | 2109.07 | 0.00 | 1825.59 | 0.00 |
| 120 | bta-miR-2285f | AAAACCTGAATGAACTTTTTGG | Yes | 3310.56 | 0.00 | 2409.15 | 0.00 | 2219.28 | 0.00 | 2672.37 | 0.00 |
| 121 | hsa-miR-15b-3p_R-1 | CGAATCATTATTTGCTGCTCT | Diff | 3249.75 | 0.00 | 2573.71 | 0.00 | 3303.20 | 0.00 | 2651.45 | 0.00 |
| 122 | bta-miR-339b | TCCCTGTCCTCCAGGAGCTC | Yes | 3248.75 | 0.00 | 2970.98 | 0.00 | 5546.97 | 0.00 | 3231.21 | 0.00 |
| 123 | bta-miR-200a | TAACACTGTCTGGTAACGATGTT | Yes | 3246.77 | 0.00 | 9345.54 | 0.00 | 10928.39 | 0.00 | 6934.80 | 0.00 |
| 124 | hsa-let-7b-3p_1ss22CT | CTATACAACCTACTGCCTTCCT | Diff | 3191.70 | 0.00 | 2865.36 | 0.00 | 2965.73 | 0.00 | 3375.04 | 0.00 |
| 125 | hsa-miR-106b-3p_L+2R-2 | TACCGCACTGTGGGTACTTGCT | Diff | 2853.82 | 0.00 | 3042.47 | 0.00 | 3581.41 | 0.00 | 3529.73 | 0.00 |
| 126 | gga-miR-30b-5p_R+1 | TGTAAACATCCTACACTCAGCTT | Diff | 2790.32 | 0.00 | 5499.19 | 0.00 | 5722.81 | 0.00 | 4009.13 | 0.00 |
| 127 | hsa-miR-542-3p | TGTGACAGATTGATAACTGAAA | Yes | 2717.88 | 0.00 | 3100.96 | 0.00 | 4580.60 | 0.00 | 2838.10 | 0.00 |
| 128 | bta-miR-504_R+1 | AGACCCTGGTCTGCACTCTGTCT | Diff | 2684.14 | 0.00 | 3207.39 | 0.00 | 3741.08 | 0.00 | 3873.62 | 0.00 |
| 129 | bta-miR-450b_R+1 | TTTTGCAATATGTTCCTGAATAT | Diff | 2679.68 | 0.00 | 3827.26 | 0.00 | 2177.65 | 0.00 | 3605.15 | 0.00 |
| 130 | ssc-miR-450b-5p_R+1 | TTTTGCAATATGTTCCTGAATAT | Diff | 2679.68 | 0.00 | 3827.26 | 0.00 | 2177.65 | 0.00 | 3605.15 | 0.00 |
| 131 | bta-miR-2285k_R+1_1 | AAAACCGGAATGAACTTTTTGG | Diff | 2607.18 | 0.00 | 1846.08 | 0.00 | 1273.26 | 0.00 | 1756.89 | 0.00 |
| 132 | bta-miR-1271 | CTTGGCACCTAGTAAGTACTCA | Yes | 2552.66 | 0.00 | 2452.66 | 0.00 | 2211.93 | 0.00 | 2118.35 | 0.00 |
| 133 | chi-miR-1271-5p_R+2 | CTTGGCACCTAGTAAGTACTCA | Diff | 2552.66 | 0.00 | 2452.66 | 0.00 | 2211.93 | 0.00 | 2118.35 | 0.00 |
| 134 | mmu-miR-452-3p_1ss20GA | TCAGTCTCATCTGCAAAGAAGT | Diff | 2481.71 | 0.00 | 3457.61 | 0.00 | 4052.59 | 0.00 | 3224.18 | 0.00 |
| 135 | mml-let-7i-3p_L+1 | CTGCGCAAGCTACTGCCTTGCT | Diff | 2323.94 | 0.00 | 1693.87 | 0.00 | 1818.13 | 0.00 | 992.06 | 0.00 |
| 136 | bta-miR-27a-5p | AGGGCTTAGCTGCTTGTGAGCA | Yes | 2269.36 | 0.00 | 1216.99 | 0.00 | 1141.23 | 0.00 | 1337.23 | 0.00 |
| 137 | hsa-miR-24-2-5p_L+1R+1 | GTGCCTACTGAGCTGAAACACAGT | Diff | 2194.45 | 0.00 | 1665.84 | 0.00 | 1690.79 | 0.00 | 2947.40 | 0.00 |
| 138 | mmu-miR-1983 | CTCACCTGGAGCATGTTTTCT | Yes | 2100.68 | 0.00 | 1969.28 | 0.00 | 1611.44 | 0.00 | 1816.64 | 0.00 |
| 139 | bta-miR-29d-3p_1ss19AG | TAGCACCATTTGAAATCGGTTA | Diff | 2085.82 | 0.00 | 3525.50 | 0.00 | 5075.39 | 0.00 | 2360.22 | 0.00 |
| 140 | bta-miR-29c | TAGCACCATTTGAAATCGGTTA | Yes | 2085.82 | 0.00 | 3525.50 | 0.00 | 5075.39 | 0.00 | 2360.22 | 0.00 |
| 141 | hsa-miR-374a-3p_1ss9AG | CTTATCAGGTTGTATTGTAATT | Diff | 2010.87 | 0.00 | 1564.29 | 0.00 | 1464.50 | 0.00 | 1702.22 | 0.00 |
| 142 | bta-miR-181b | AACATTCATTGCTGTCGGTGGGTT | Yes | 1981.26 | 0.00 | 2057.02 | 0.00 | 1510.22 | 0.00 | 2107.70 | 0.00 |
| 143 | bta-miR-660 | TACCCATTGCATATCGGAGCTG | Yes | 1945.88 | 0.00 | 1908.35 | 0.00 | 2280.50 | 0.00 | 1967.49 | 0.00 |
| 144 | bta-miR-196a | TAGGTAGTTTCATGTTGTTGGG | Yes | 1859.54 | 0.00 | 2309.94 | 0.00 | 1855.36 | 0.00 | 2059.54 | 0.00 |
| 145 | bta-miR-2478_L-2 | ATCCCACTTCTGACACCA | Diff | 1738.49 | 0.00 | 2312.11 | 0.00 | 3663.69 | 0.00 | 3215.23 | 0.00 |
| 146 | bta-miR-2285k_R+1_2 | AAAACCGGAATGAACTTTTTGT | Diff | 1738.12 | 0.00 | 1230.72 | 0.00 | 848.40 | 0.00 | 1171.26 | 0.00 |
| 147 | ggo-miR-361 | TTATCAGAATCTCCAGGGGTAC | Yes | 1734.02 | 0.00 | 2104.54 | 0.00 | 2053.73 | 0.00 | 1819.83 | 0.00 |
| 148 | hsa-miR-361-5p | TTATCAGAATCTCCAGGGGTAC | Yes | 1734.02 | 0.00 | 2104.54 | 0.00 | 2053.73 | 0.00 | 1819.83 | 0.00 |
| 149 | bta-miR-874_R+1 | CTGCCCTGGCCCGAGGGACCGAC | Diff | 1690.86 | 0.00 | 1060.19 | 0.00 | 914.94 | 0.00 | 1001.01 | 0.00 |
| 150 | bta-miR-532 | CATGCCTTGAGTGTAGGACCGT | Yes | 1677.96 | 0.00 | 1385.16 | 0.00 | 1388.09 | 0.00 | 1255.41 | 0.00 |
| 151 | bta-miR-33a_R-1 | GTGCATTGTAGTTGCATTGC | Diff | 1641.25 | 0.00 | 1378.66 | 0.00 | 2854.55 | 0.00 | 1334.67 | 0.00 |
| 152 | bta-miR-6120-3p | TATGTTGGACAACGTGGATAGC | Yes | 1605.52 | 0.00 | 1558.20 | 0.00 | 1368.50 | 0.00 | 1527.72 | 0.00 |
| 153 | bta-miR-194 | TGTAACAGCAACTCCATGTGGA | Yes | 1568.84 | 0.00 | 2056.20 | 0.00 | 2182.54 | 0.00 | 1670.90 | 0.00 |
| 154 | bta-miR-374a_R+1 | TTATAATACAACCTGATAAGTGT | Diff | 1541.02 | 0.00 | 1356.32 | 0.00 | 1069.72 | 0.00 | 1601.86 | 0.00 |
| 155 | mml-miR-374a-5p_R+1 | TTATAATACAACCTGATAAGTGT | Diff | 1541.02 | 0.00 | 1356.32 | 0.00 | 1069.72 | 0.00 | 1601.86 | 0.00 |
| 156 | bta-miR-505_L-1R+2 | GTCAACACTTGCTGGTTTCCTCT | Diff | 1497.36 | 0.00 | 1303.10 | 0.00 | 1449.80 | 0.00 | 1668.34 | 0.00 |
| 157 | bta-miR-6529a | GAGAGATCAGAGGCGCAGAGT | Yes | 1446.76 | 0.00 | 1039.07 | 0.00 | 1627.11 | 0.00 | 1287.37 | 0.00 |
| 158 | bta-miR-17-3p_L-1R+4 | CTGCAGTGAAGGCACTTGTAGCT | Diff | 1410.04 | 0.00 | 1252.73 | 0.00 | 1275.44 | 0.00 | 792.62 | 0.00 |
| 159 | bta-miR-744 | TGCGGGGCTAGGGCTAACAGCA | Yes | 1407.07 | 0.00 | 1339.66 | 0.00 | 1994.46 | 0.00 | 1498.31 | 0.00 |
| 160 | bta-miR-190a_R+1 | TGATATGTTTGATATATTAGGTT | Diff | 1394.17 | 0.00 | 1498.08 | 0.00 | 1197.07 | 0.00 | 1017.62 | 0.00 |
| 161 | bta-miR-1271_R-2 | CTTGGCACCTAGTAAGTACT | Diff | 1334.63 | 0.00 | 861.97 | 0.00 | 994.29 | 0.00 | 911.52 | 0.00 |
| 162 | bta-miR-95_R-1 | TTCAACGGGTATTTATTGAGC | Diff | 1320.74 | 0.00 | 3157.83 | 0.00 | 3178.79 | 0.00 | 2839.38 | 0.00 |
| 163 | bta-miR-193a-5p | TGGGTCTTTGCGGGCGAGATGA | Yes | 1280.05 | 0.00 | 1447.71 | 0.00 | 1691.76 | 0.00 | 1839.65 | 0.00 |
| 164 | bta-miR-331-5p_R+4 | TCTAGGTATGGTCCCAGGGATC | Diff | 1230.44 | 0.00 | 1203.58 | 0.00 | 1209.31 | 0.00 | 1139.29 | 0.00 |
| 165 | bta-miR-19a_2ss11TC23AT | TGTGCAAATCCATGCAAAACTGT | Diff | 1206.79 | 0.00 | 4556.93 | 0.00 | 572.25 | 0.00 | 3082.28 | 0.00 |
| 166 | bta-miR-24 | GTGCCTACTGAGCTGATATCAGT | Yes | 1204.14 | 0.00 | 967.17 | 0.00 | 1293.07 | 0.00 | 1484.89 | 0.00 |
| 167 | hsa-miR-615-3p_R-1 | TCCGAGCCTGGGTCTCCCTCT | Diff | 1195.71 | 0.00 | 576.00 | 0.00 | 973.72 | 0.00 | 819.47 | 0.00 |
| 168 | bta-miR-130b | CAGTGCAATGATGAAAGGGCAT | Yes | 1146.09 | 0.00 | 999.67 | 0.00 | 1109.88 | 0.00 | 738.93 | 0.00 |
| 169 | hsa-miR-500a-3p_R-1 | ATGCACCTGGGCAAGGATTCT | Diff | 1121.62 | 0.00 | 1061.82 | 0.00 | 886.37 | 0.00 | 966.27 | 0.00 |
| 170 | bta-miR-486_R+1 | TCCTGTACTGAGCTGCCCCGAGT | Diff | 1108.39 | 0.00 | 2074.89 | 0.00 | 1712.34 | 0.00 | 2087.66 | 0.00 |
| 171 | hsa-miR-30e-3p | CTTTCAGTCGGATGTTTACAGC | Yes | 1093.01 | 0.00 | 1493.21 | 0.00 | 1179.93 | 0.00 | 1140.99 | 0.00 |
| 172 | bta-miR-197 | TTCACCACCTTCTCCACCCAGC | Yes | 1087.55 | 0.00 | 879.03 | 0.00 | 1218.62 | 0.00 | 960.10 | 0.00 |
| 173 | hsa-miR-27b-5p_R+1 | AGAGCTTAGCTGATTGGTGAACA | Diff | 1083.58 | 0.00 | 1117.06 | 0.00 | 933.56 | 0.00 | 1266.92 | 0.00 |
| 174 | hsa-miR-16-2-3p_L+1R-1_1ss11CT | ACCAATATTATTGTGCTGCTTT | Diff | 1065.72 | 0.00 | 881.46 | 0.00 | 811.11 | 0.00 | 608.53 | 0.00 |
| 175 | aca-miR-191-5p_R+3_2 | CAACGGAATCCCAAAAGCAGCTGTTT | Diff | 1035.95 | 0.00 | 2118.76 | 0.00 | 216.49 | 0.00 | 1691.35 | 0.00 |
| 176 | aca-miR-191-5p_R+3_1 | CAACGGAATCCCAAAAGCAGCTGTAT | Diff | 1035.95 | 0.00 | 2118.76 | 0.00 | 216.49 | 0.00 | 1691.35 | 0.00 |
| 177 | hsa-miR-16-1-3p_L+1_1ss23AT | TCCAGTATTAACTGTGCTGCTGT | Diff | 1031.98 | 0.00 | 1103.25 | 0.00 | 924.74 | 0.00 | 641.77 | 0.00 |
| 178 | bta-miR-195 | TAGCAGCACAGAAATATTGGCA | Yes | 1025.36 | 0.00 | 1063.71 | 0.00 | 1011.92 | 0.00 | 913.65 | 0.00 |
| 179 | hsa-let-7d-3p | CTATACGACCTGCTGCCTTTCT | Yes | 992.29 | 0.00 | 697.05 | 0.00 | 1112.82 | 0.00 | 1664.51 | 0.00 |
| 180 | mdo-let-7f-2-3p_1ss8GA | CTATACAATCTACTGTCTTTCC | Diff | 988.32 | 0.00 | 1196.41 | 0.00 | 987.92 | 0.00 | 1044.90 | 0.00 |
| 181 | bta-let-7a-3p_R+1_1 | CTATACAATCTACTGTCTTTCC | Diff | 988.32 | 0.00 | 1196.41 | 0.00 | 987.92 | 0.00 | 1044.90 | 0.00 |
| 182 | bta-let-7a-3p_R+1_2 | CTATACAATCTACTGTCTTTCT | Diff | 988.32 | 0.00 | 1196.41 | 0.00 | 987.92 | 0.00 | 1044.90 | 0.00 |
| 183 | bta-miR-455-3p | GCAGTCCATGGGCATATACACT | Yes | 970.46 | 0.00 | 910.71 | 0.00 | 973.72 | 0.00 | 820.75 | 0.00 |
| 184 | bta-miR-503-5p_R+3 | TAGCAGCGGGAACAGTACTGCAG | Diff | 963.51 | 0.00 | 1824.67 | 0.00 | 1486.05 | 0.00 | 1089.22 | 0.00 |
| 185 | bta-miR-22-5p_R-1 | AGTTCTTCAGTGGCAAGCTTT | Diff | 944.66 | 0.00 | 1210.49 | 0.00 | 1431.19 | 0.00 | 972.88 | 0.00 |
| 186 | hsa-miR-181a-3p | ACCATCGACCGTTGATTGTACC | Yes | 939.20 | 0.00 | 648.30 | 0.00 | 780.74 | 0.00 | 752.35 | 0.00 |
| 187 | PC-3p-1367_1185 | AAAAAACCGAGTGGACTTTTTGT | New | 923.82 | 0.00 | 636.12 | 0.00 | 480.00 | 0.00 | 653.27 | 0.00 |
| 188 | hsa-miR-10a-3p_R-1 | CAAATTCGTATCTAGGGGAAT | Diff | 913.90 | 0.00 | 914.77 | 0.00 | 986.45 | 0.00 | 855.26 | 0.00 |
| 189 | hsa-miR-339-5p_R-1_1ss22CT | TCCCTGTCCTCCAGGAGCTCAT | Diff | 896.04 | 0.00 | 880.65 | 0.00 | 1550.70 | 0.00 | 763.22 | 0.00 |
| 190 | hhi-miR-26_R+3 | TTCAAGTAATCCAGGATAGGCTTA | Diff | 876.19 | 0.00 | 3788.26 | 0.00 | 1466.46 | 0.00 | 2610.54 | 0.00 |
| 191 | hsa-miR-30d-3p | CTTTCAGTCAGATGTTTGCTGC | Yes | 842.45 | 0.00 | 645.46 | 0.00 | 738.62 | 0.00 | 819.47 | 0.00 |
| 192 | mmu-miR-29a-5p_R+1 | ACTGATTTCTTTTGGTGTTCAGA | Diff | 829.55 | 0.00 | 1440.40 | 0.00 | 965.88 | 0.00 | 837.37 | 0.00 |
| 193 | hsa-miR-140-5p | CAGTGGTTTTACCCTATGGTAG | Yes | 785.89 | 0.00 | 1012.26 | 0.00 | 600.49 | 0.00 | 828.42 | 0.00 |
| 194 | bta-miR-2285g_R+1 | AAACCTGAACAAGCTTTTTGGC | Diff | 775.74 | 0.00 | 528.15 | 0.00 | 437.01 | 0.00 | 658.17 | 0.00 |
| 195 | bta-miR-301a_R-2 | CAGTGCAATAGTATTGTCAAAGC | Diff | 768.03 | 0.00 | 766.91 | 0.00 | 721.96 | 0.00 | 675.01 | 0.00 |
| 196 | bta-miR-1839_R-2 | AAGGTAGATAGAACAGGTCTTG | Diff | 749.18 | 0.00 | 1807.61 | 0.00 | 1897.48 | 0.00 | 1998.17 | 0.00 |
| 197 | bta-miR-340_R-1 | TCCGTCTCAGTTACTTTATAGC | Diff | 748.19 | 0.00 | 701.92 | 0.00 | 761.15 | 0.00 | 1066.20 | 0.00 |
| 198 | hsa-miR-224-3p_L-1_1ss9GA | AAATGGTACCCTAGTGACTACA | Diff | 737.27 | 0.00 | 741.73 | 0.00 | 909.07 | 0.00 | 700.58 | 0.00 |
| 199 | oha-miR-27a-3p_R+1_1ss22CA | TTCACAGTGGCTAAGTTCCGCAT | Diff | 730.32 | 0.00 | 1286.72 | 0.00 | 885.56 | 0.00 | 1001.86 | 0.00 |
| 200 | bta-miR-2419-5p | ATCGCATCAACACTCGTCTGTT | Yes | 720.40 | 0.00 | 546.75 | 0.00 | 726.86 | 0.00 | 522.87 | 0.00 |
| 201 | hsa-miR-148b-5p_L+1R+1 | GAAGTTCTGTTATACACTCAGGCT | Diff | 714.45 | 0.00 | 740.10 | 0.00 | 656.33 | 0.00 | 880.83 | 0.00 |
| 202 | ggo-miR-574 | CACGCTCATGCACACACCCACA | Yes | 708.99 | 0.00 | 682.15 | 0.00 | 836.90 | 0.00 | 627.28 | 0.00 |
| 203 | hsa-miR-574-3p | CACGCTCATGCACACACCCACA | Yes | 708.99 | 0.00 | 682.15 | 0.00 | 836.90 | 0.00 | 627.28 | 0.00 |
| 204 | hsa-miR-33a-3p_R-1 | CAATGTTTCCACAGTGCATCA | Diff | 683.69 | 0.00 | 852.22 | 0.00 | 1016.82 | 0.00 | 605.97 | 0.00 |
| 205 | bta-miR-2284aa_R+1 | AAAAAAGTTTGTTTGGGTTTTT | Diff | 679.95 | 0.00 | 638.95 | 0.00 | 745.55 | 0.00 | 475.55 | 0.00 |
| 206 | bta-miR-455-5p_R+1 | TATGTGCCTTTGGACTACATCG | Diff | 676.74 | 0.00 | 467.13 | 0.00 | 614.21 | 0.00 | 455.12 | 0.00 |
| 207 | hsa-miR-125b-1-3p | ACGGGTTAGGCTCTTGGGAGCT | Yes | 675.75 | 0.00 | 304.65 | 0.00 | 301.72 | 0.00 | 396.31 | 0.00 |
| 208 | ssc-miR-7857-3p_R+1 | ATTGTTCTCCAACCTGGCTCTTT | Diff | 673.76 | 0.00 | 489.88 | 0.00 | 429.06 | 0.00 | 476.85 | 0.00 |
| 209 | hsa-miR-34a-3p | CAATCAGCAAGTATACTGCCCT | Yes | 622.17 | 0.00 | 496.38 | 0.00 | 633.80 | 0.00 | 406.54 | 0.00 |
| 210 | bta-miR-2318 | GTGTATGATGAATTATCTGACC | Yes | 619.19 | 0.00 | 576.81 | 0.00 | 527.02 | 0.00 | 622.59 | 0.00 |
| 211 | bta-miR-362-5p | AATCCTTGGAACCTAGGTGTGAGT | Yes | 616.21 | 0.00 | 895.27 | 0.00 | 402.61 | 0.00 | 761.94 | 0.00 |
| 212 | mmu-let-7f-1-3p_1ss22CT | CTATACAATCTATTGCCTTCCT | Diff | 607.28 | 0.00 | 621.90 | 0.00 | 522.13 | 0.00 | 562.51 | 0.00 |
| 213 | bta-miR-2285e | AAACCTGAACGAACTTTTTGGC | Yes | 596.70 | 0.00 | 673.90 | 0.00 | 510.70 | 0.00 | 580.40 | 0.00 |
| 214 | hsa-miR-222-5p_L+2R-1 | GGCTCAGTAGCCAGTGTAGATCC | Diff | 595.37 | 0.00 | 385.89 | 0.00 | 461.39 | 0.00 | 420.60 | 0.00 |
| 215 | bta-miR-2483-5p | CGTCAACCATCCAGCTGTTTGA | Yes | 582.47 | 0.00 | 519.13 | 0.00 | 519.19 | 0.00 | 396.31 | 0.00 |
| 216 | bta-mir-2284z-2-p3 | AAACCCAGATGAACTTTTTGGC | New | 557.17 | 0.00 | 361.55 | 0.00 | 393.73 | 0.00 | 446.81 | 0.00 |
| 217 | bta-miR-454_R+2 | TAGTGCAATATTGCTTATAGGGTTT | Diff | 551.71 | 0.00 | 784.79 | 0.00 | 328.17 | 0.00 | 618.76 | 0.00 |
| 218 | hsa-miR-33b-3p_R-1 | CAGTGCCTCGGCAGTGCAGCC | Diff | 533.85 | 0.00 | 144.61 | 0.00 | 176.33 | 0.00 | 175.14 | 0.00 |
| 219 | hsa-miR-10b-3p | ACAGATTCGATTCTAGGGGAAT | Yes | 525.91 | 0.00 | 710.86 | 0.00 | 694.53 | 0.00 | 611.09 | 0.00 |
| 220 | bta-miR-29d-5p_R+2 | TGACCGATTTCTCCTGGTGTTCT | Diff | 517.98 | 0.00 | 901.78 | 0.00 | 669.06 | 0.00 | 727.42 | 0.00 |
| 221 | mmu-let-7j_1ss8TG | TGAGGTAGTAGTTTGTGCTGTTAT | Diff | 516.98 | 0.00 | 1193.43 | 0.00 | 561.31 | 0.00 | 1210.67 | 0.00 |
| 222 | PC-3p-1139_1526 | AAACTCAAACAAACTTTTTGGT | New | 515.99 | 0.00 | 351.37 | 0.00 | 360.00 | 0.00 | 356.04 | 0.00 |
| 223 | bta-miR-449b_R+3 | AGGCAGTGTATTGTTAGCTGGCTGC | Diff | 515.00 | 0.00 | 340.40 | 0.00 | 198.86 | 0.00 | 216.69 | 0.00 |
| 224 | bta-miR-2284t-3p | AAACTCGAATGAATGTTTTGGC | Yes | 486.22 | 0.00 | 369.65 | 0.00 | 357.55 | 0.00 | 401.42 | 0.00 |
| 225 | hsa-miR-130b-5p_R+1 | ACTCTTTCCCTGTTGCACTACT | Diff | 473.32 | 0.00 | 536.19 | 0.00 | 594.62 | 0.00 | 608.53 | 0.00 |
| 226 | bta-miR-2336 | CTAACCGTAACTTTGAAGTGCT | Yes | 468.36 | 0.00 | 352.59 | 0.00 | 397.72 | 0.00 | 405.26 | 0.00 |
| 227 | bta-mir-3431-p3 | ATCTAGAGGACTGACTGAAATT | New | 467.37 | 0.00 | 789.66 | 0.00 | 755.27 | 0.00 | 843.76 | 0.00 |
| 228 | bta-miR-2285n_L-1R+1_1ss21GT | AAAACCCGAATGAACTTTTTGG | Diff | 464.94 | 0.00 | 335.88 | 0.00 | 338.16 | 0.00 | 389.10 | 0.00 |
| 229 | bta-miR-497_R-1 | CAGCAGCACACTGTGGTTTGT | Diff | 440.58 | 0.00 | 514.25 | 0.00 | 451.59 | 0.00 | 313.21 | 0.00 |
| 230 | hsa-miR-361-3p | TCCCCCAGGTGTGATTCTGATTT | Yes | 430.66 | 0.00 | 661.30 | 0.00 | 307.57*2 | #VALUE! | 852.00 | 0.00 |
| 231 | bta-miR-7859 | AAAAACTGGCAGCTTCATGTAA | Yes | 421.72 | 0.00 | 484.20 | 0.00 | 578.94 | 0.00 | 543.33 | 0.00 |
| 232 | bta-miR-20b_R+3_1ss10CT | CAAAGTGCTTACAGTGCAGGTAGTT | Diff | 420.74 | 0.00 | 859.44 | 0.00 | 200.82 | 0.00 | 691.62 | 0.00 |
| 233 | hsa-miR-30c-2-3p | CTGGGAGAAGGCTGTTTACTCT | Yes | 411.80 | 0.00 | 627.99 | 0.00 | 586.78 | 0.00 | 705.69 | 0.00 |
| 234 | dre-miR-148_R+3_1ss10TC | TCAGTGCATCACAGAACTTTGTTTT | Diff | 406.84 | 0.00 | 528.88 | 0.00 | 311.02 | 0.00 | 673.73 | 0.00 |
| 235 | bta-miR-1306 | CCACCTCCCCTGCAAACGTCC | Yes | 402.87 | 0.00 | 355.02 | 0.00 | 467.27 | 0.00 | 419.32 | 0.00 |
| 236 | hsa-miR-7-1-3p | CAACAAATCACAGTCTGCCATA | Yes | 397.91 | 0.00 | 353.40 | 0.00 | 351.68 | 0.00 | 308.10 | 0.00 |
| 237 | bta-miR-425-3p_L+1R-1 | CATCGGGAATGTCGTGTCCGCC | Diff | 396.92 | 0.00 | 439.51 | 0.00 | 491.76 | 0.00 | 323.44 | 0.00 |
| 238 | bta-miR-2344 | GCACGATGATGGCGGATCTGAGTT | Yes | 396.92 | 0.00 | 283.53 | 0.00 | 229.23 | 0.00 | 334.95 | 0.00 |
| 239 | bta-miR-23b-5p | GGGTTCCTGGCATGCTGATTT | Yes | 394.93 | 0.00 | 374.52 | 0.00 | 544.66 | 0.00 | 623.87 | 0.00 |
| 240 | bta-miR-429 | TAATACTGTCTGGTAATGCCGT | Yes | 390.96 | 0.00 | 899.34 | 0.00 | 1113.80 | 0.00 | 714.64 | 0.00 |
| 241 | mmu-miR-26a-2-3p_1ss4GA | CCTATTCTTGATTACTTGTTTC | Diff | 385.01 | 0.00 | 311.15 | 0.00 | 313.47 | 0.00 | 342.62 | 0.00 |
| 242 | bta-miR-135a | TATGGCTTTTTATTCCTATGTGA | Yes | 381.04 | 0.00 | 1167.44 | 0.00 | 665.14 | 0.00 | 637.94 | 0.00 |
| 243 | bta-miR-324 | CGCATCCCCTAGGGCATTGGTGT | Yes | 372.11 | 0.00 | 385.08 | 0.00 | 462.37 | 0.00 | 359.24 | 0.00 |
| 244 | cgr-miR-615-3p | TCCGAGCCTGGGTCTCCCTCTT | Yes | 368.14 | 0.00 | 272.96 | 0.00 | 320.32 | 0.00 | 301.70 | 0.00 |
| 245 | bta-miR-4286_R+1 | ACCCCACTCCTGGTACCA | Diff | 365.16 | 0.00 | 560.56 | 0.00 | 771.40 | 0.00 | 551.00 | 0.00 |
| 246 | bta-miR-338_R-2 | TCCAGCATCAGTGATTTTGTT | Diff | 351.27 | 0.00 | 298.97 | 0.00 | 441.80 | 0.00 | 281.25 | 0.00 |
| 247 | PC-3p-1996_691 | AAAACTCGAGCAAACTTTTTGG | New | 335.39 | 0.00 | 224.22 | 0.00 | 199.35 | 0.00 | 312.57 | 0.00 |
| 248 | ssa-miR-10d-5p_L-1R+3 | ACCCTGTAGAACCGAATTTGTGTT | Diff | 333.41 | 0.00 | 1091.06 | 0.00 | 272.33 | 0.00 | 779.84 | 0.00 |
| 249 | bta-miR-2285ab_L+1R-2 | AAAAACCTGAATGAACTTCTT | Diff | 327.79 | 0.00 | 190.10 | 0.00 | 216.49 | 0.00 | 172.59 | 0.00 |
| 250 | PC-3p-4634_200 | AAAACCTGAATGACCCTTTTGG | New | 321.01 | 0.00 | 128.77 | 0.00 | 154.78 | 0.00 | 145.74 | 0.00 |
| 251 | bta-miR-2285q | AAGGACCTGAATGAACTTTCTGG | Yes | 320.84 | 0.00 | 262.57 | 0.00 | 195.92 | 0.00 | 293.44 | 0.00 |
| 252 | bta-miR-424-3p_R+1 | CAAAACGTGAGGCGCTGCTATA | Diff | 316.54 | 0.00 | 481.76 | 0.00 | 561.31 | 0.00 | 396.31 | 0.00 |
| 253 | mmu-miR-15a-3p_2ss10CT22AT | CAGGCCATATTGTGCTGCCTCT | Diff | 315.55 | 0.00 | 207.98 | 0.00 | 186.12 | 0.00 | 125.29 | 0.00 |
| 254 | bta-miR-545-3p_L-1R+2 | TCAACAAACATTTATTGTGTGCC | Diff | 315.54 | 0.00 | 173.86 | 0.00 | 244.90 | 0.00 | 187.92 | 0.00 |
| 255 | ggo-miR-652_R+1 | AATGGCGCCACTAGGGTTGTGT | Diff | 312.07 | 0.00 | 305.06 | 0.00 | 359.51 | 0.00 | 292.76 | 0.00 |
| 256 | cgr-miR-652-3p_R+1 | AATGGCGCCACTAGGGTTGTGT | Diff | 312.07 | 0.00 | 305.06 | 0.00 | 359.51 | 0.00 | 292.76 | 0.00 |
| 257 | bta-miR-184 | TGGACGGAGAACTGATAAGGGT | Yes | 311.58 | 0.00 | 239.66 | 0.00 | 240.98 | 0.00 | 219.89 | 0.00 |
| 258 | hsa-miR-196b-3p_R+1 | TCGACAGCACGACACTGCCTTCA | Diff | 310.59 | 0.00 | 333.90 | 0.00 | 388.90 | 0.00 | 168.75 | 0.00 |
| 259 | bta-miR-2285b | AAAATCTGAGTGAACTTTTTGG | Yes | 310.46 | 0.00 | 211.80 | 0.00 | 173.68 | 0.00 | 239.84 | 0.00 |
| 260 | bta-miR-2387_R+1 | TGGAAGGCCTGGCTTTGCAGCGT | Diff | 308.60 | 0.00 | 260.78 | 0.00 | 206.70 | 0.00 | 171.31 | 0.00 |
| 261 | xtr-miR-221_R+2 | AGCTACATTGTCTGCTGGGTTTCTT | Diff | 302.65 | 0.00 | 558.94 | 0.00 | 195.92 | 0.00 | 686.51 | 0.00 |
| 262 | PC-5p-2325_556 | ATAATGCACATGTATGAACATA | New | 298.68 | 0.00 | 136.48 | 0.00 | 144.00 | 0.00 | 175.14 | 0.00 |
| 263 | bta-miR-500_R+2 | TAATCCTTGCTACCTGGGTGAGAGT | Diff | 292.73 | 0.00 | 358.27 | 0.00 | 168.49 | 0.00 | 177.70 | 0.00 |
| 264 | hsa-miR-338-5p_R-1 | AACAATATCCTGGTGCTGAGT | Diff | 272.88 | 0.00 | 236.41 | 0.00 | 258.61 | 0.00 | 193.04 | 0.00 |
| 265 | bta-miR-2285j_L-1R+1 | AAAACCAGAACGAACTTTTTGT | Diff | 272.32 | 0.00 | 186.48 | 0.00 | 172.92 | 0.00 | 195.96 | 0.00 |
| 266 | hsa-miR-148a-5p | AAAGTTCTGAGACACTCCGACT | Yes | 265.93 | 0.00 | 251.03 | 0.00 | 295.84 | 0.00 | 333.67 | 0.00 |
| 267 | hsa-miR-191-3p_L-1_1ss19CT | CTGCGCTTGGATTTCGTTCCC | Diff | 265.93 | 0.00 | 274.59 | 0.00 | 378.12 | 0.00 | 336.23 | 0.00 |
| 268 | bta-miR-1388-5p_R+1 | AGGACTGTCCAACCTGAGAATG | Diff | 262.96 | 0.00 | 234.79 | 0.00 | 223.35 | 0.00 | 195.60 | 0.00 |
| 269 | bta-miR-362-3p_R+1 | AACACACCTATTCAAGGATTCA | Diff | 255.02 | 0.00 | 290.03 | 0.00 | 300.74 | 0.00 | 158.52 | 0.00 |
| 270 | mmu-miR-190a-3p_1ss13GA | ACTATATATCAAACATATTCCT | Diff | 247.08 | 0.00 | 289.22 | 0.00 | 216.49 | 0.00 | 145.74 | 0.00 |
| 271 | bta-mir-2284f-p3_1ss22CT | AAACCCGAACAAACTTTTTGGT | New | 247.06 | 0.00 | 133.39 | 0.00 | 163.44 | 0.00 | 137.25 | 0.00 |
| 272 | bta-miR-2284aa_L-1R+2_1ss10TC | AAAAAGTTCGTTTGGGTTTTCT | Diff | 245.08 | 0.00 | 187.76 | 0.00 | 223.34 | 0.00 | 189.52 | 0.00 |
| 273 | bta-miR-542-5p_R+1 | TCGGGGATCATCATGTCACGAGA | Diff | 243.11 | 0.00 | 328.21 | 0.00 | 394.78 | 0.00 | 239.07 | 0.00 |
| 274 | bta-miR-30b-3p_R+1 | CTGGGAGGTGGATGTTTACTTC | Diff | 239.14 | 0.00 | 240.47 | 0.00 | 216.49 | 0.00 | 264.63 | 0.00 |
| 275 | hsa-miR-125a-3p | ACAGGTGAGGTTCTTGGGAGCC | Yes | 231.20 | 0.00 | 177.11 | 0.00 | 151.84 | 0.00 | 126.56 | 0.00 |
| 276 | bta-miR-3432a_R+1 | TGCGGGATCTTTAGTTGTGGTGT | Diff | 229.22 | 0.00 | 251.03 | 0.00 | 228.25 | 0.00 | 170.03 | 0.00 |
| 277 | chi-miR-345-5p_R+1 | GCTGACTCCTAGTCCAGTGCTT | Diff | 224.26 | 0.00 | 191.72 | 0.00 | 246.86 | 0.00 | 132.96 | 0.00 |
| 278 | gga-miR-3535_L+1_1ss25AG | TGGATATGATGACTGATTATCTGAGA | Diff | 219.30 | 0.00 | 759.60 | 0.00 | 287.02 | 0.00 | 576.57 | 0.00 |
| 279 | bta-miR-491_R+1 | AGTGGGGAACCCTTCCATGAGGA | Diff | 209.37 | 0.00 | 271.34 | 0.00 | 217.47 | 0.00 | 153.41 | 0.00 |
| 280 | ssc-miR-339-3p_1ss1AC | CGCTCCTCGAGGCCAGAGCCC | Diff | 206.40 | 0.00 | 144.61 | 0.00 | 170.45 | 0.00 | 129.12 | 0.00 |
| 281 | bta-miR-2320-5p | TGGCACAGGGTCCAGCTGTCGGC | Yes | 206.40 | 0.00 | 45.49 | 0.00 | 77.39 | 0.00 | 109.94 | 0.00 |
| 282 | bta-miR-1343-3p_R+3 | CTCCTGGGGCCCGCACTCTCGCT | Diff | 205.40 | 0.00 | 214.48 | 0.00 | 228.25 | 0.00 | 343.90 | 0.00 |
| 283 | hsa-miR-98-3p_1ss22CT | CTATACAACTTACTACTTTCCT | Diff | 201.43 | 0.00 | 289.22 | 0.00 | 265.47 | 0.00 | 176.42 | 0.00 |
| 284 | hsa-miR-1306-3p_R+4 | ACGTTGGCTCTGGTGGTGATGT | Diff | 196.47 | 0.00 | 192.54 | 0.00 | 223.35 | 0.00 | 213.50 | 0.00 |
| 285 | bta-let-7c_R+2_1ss17AG | TGAGGTAGTAGGTTGTGTGGTTTA | Diff | 196.23 | 0.00 | 368.11 | 0.00 | 368.36 | 0.00 | 435.69 | 0.00 |
| 286 | cin-miR-92c-3p_1ss18TC | TATTGCACTCGTCCCGGCCTAT | Diff | 194.98 | 0.00 | 133.23 | 0.00 | 161.63 | 0.00 | 138.07 | 0.00 |
| 287 | ssc-miR-339_R+1_1ss21AT | TCCCTGTCCTCCAGGAGCTCTT | Diff | 192.83 | 0.00 | 246.97 | 0.00 | 295.51 | 0.00 | 220.95 | 0.00 |
| 288 | bta-mir-2285ac-p5_1ss12TG | GAAAGTTTGTTGGGGTTTTT | New | 192.50 | 0.00 | 224.34 | 0.00 | 235.73 | 0.00 | 214.96 | 0.00 |
| 289 | bta-miR-1246_L+1R+4 | AAATGGATTTTTGGAGCAGGGAGT | Diff | 191.51 | 0.00 | 95.86 | 0.00 | 189.06 | 0.00 | 222.45 | 0.00 |
| 290 | PC-5p-5211_167 | TGGACAGGCCAAGCCGCTGTGC | New | 189.53 | 0.00 | 134.86 | 0.00 | 137.14 | 0.00 | 108.67 | 0.00 |
| 291 | bta-mir-2285ab-p5 | GAAAGTTTGTTCTGGTCTTTCT | New | 188.53 | 0.00 | 137.30 | 0.00 | 133.23 | 0.00 | 90.77 | 0.00 |
| 292 | bta-miR-2284d_L-1R+1_1ss10GA | AAAAGTTCATTAGGGTTTTTCT | Diff | 187.52 | 0.00 | 174.10 | 0.00 | 182.65 | 0.00 | 137.54 | 0.00 |
| 293 | bta-mir-2285b-2-p5_1ss12TA | AAAAGTTCATTAGGGTTTTTCT | New | 187.52 | 0.00 | 174.10 | 0.00 | 182.65 | 0.00 | 137.54 | 0.00 |
| 294 | bta-mir-2285k-3-p5_1ss12TA | AAAAGTTCATTAGGGTTTTTCT | New | 187.52 | 0.00 | 174.10 | 0.00 | 182.65 | 0.00 | 137.54 | 0.00 |
| 295 | bta-miR-188 | CATCCCTTGCATGGTGGAGGGT | Yes | 186.55 | 0.00 | 185.23 | 0.00 | 208.65 | 0.00 | 132.96 | 0.00 |
| 296 | bta-miR-2299-5p | ACTGGATTCATTTCTCTGAAA | Yes | 185.56 | 0.00 | 140.55 | 0.00 | 97.96 | 0.00 | 98.44 | 0.00 |
| 297 | bta-miR-1296_R-2 | TTAGGGCCCTGGCTCCATCT | Diff | 184.57 | 0.00 | 148.67 | 0.00 | 214.53 | 0.00 | 154.69 | 0.00 |
| 298 | bta-mir-2285e-2-p5_1ss21TC | AAAAAGTTCGTTTGGGTTTTC | New | 182.39 | 0.00 | 149.08 | 0.00 | 189.11 | 0.00 | 150.92 | 0.00 |
| 299 | bta-miR-451_R-1 | AAACCGTTACCATTACTGAGTT | Diff | 181.59 | 0.00 | 160.86 | 0.00 | 184.16 | 0.00 | 23.01 | 0.00 |
| 300 | mmu-miR-20a-3p_L-1R+1_1ss10CT | CTGCATTATGAGCACTTAAAGT | Diff | 179.60 | 0.00 | 270.53 | 0.00 | 174.37 | 0.00 | 181.54 | 0.00 |
| 301 | bta-miR-2285h_R+1 | GAAAACCGAAACGAACTTTATGT | Diff | 178.04 | 0.00 | 112.46 | 0.00 | 104.26 | 0.00 | 119.08 | 0.00 |
| 302 | bta-mir-93-p3_1ss22CT | CTACTGCTGAGCCAGCACTTCT | New | 177.62 | 0.00 | 134.86 | 0.00 | 192.98 | 0.00 | 134.23 | 0.00 |
| 303 | hsa-miR-26b-3p | CCTGTTCTCCATTACTTGGCTC | Yes | 176.63 | 0.00 | 173.04 | 0.00 | 178.29 | 0.00 | 190.49 | 0.00 |
| 304 | mml-miR-6529-3p_R-1 | CCTGTGCCTTTTACCTCTTTA | Diff | 175.64 | 0.00 | 201.48 | 0.00 | 192.00 | 0.00 | 170.03 | 0.00 |
| 305 | ssa-miR-21a-5p_R-1 | TAGCTTATCAGACTGGTGTTGAC | Diff | 174.64 | 0.00 | 166.54 | 0.00 | 206.70 | 0.00 | 346.45 | 0.00 |
| 306 | bta-miR-146a_R-2 | TGAGAACTGAATTCCATAGGTT | Diff | 173.65 | 0.00 | 140.14 | 0.00 | 244.90 | 0.00 | 480.69 | 0.00 |
| 307 | ggo-miR-421_R+1 | ATCAACAGACATTAATTGGGCGT | Diff | 173.65 | 0.00 | 216.10 | 0.00 | 192.98 | 0.00 | 221.17 | 0.00 |
| 308 | PC-3p-3939_256 | AAACTTGAATGAACCTTTTGGC | New | 173.15 | 0.00 | 94.65 | 0.00 | 80.82 | 0.00 | 120.17 | 0.00 |
| 309 | oha-miR-30b-5p_R+3 | TGTAAACATCCTACACTCAGCTTTT | Diff | 170.67 | 0.00 | 727.92 | 0.00 | 240.00 | 0.00 | 342.62 | 0.00 |
| 310 | bta-miR-2284y | AAAAGTTCGTTCGGGTTTTTC | Yes | 167.79 | 0.00 | 196.00 | 0.00 | 211.61 | 0.00 | 195.16 | 0.00 |
| 311 | bta-miR-33b_R-1 | GTGCATTGCTGTTGCATTG | Diff | 164.72 | 0.00 | 160.86 | 0.00 | 251.76 | 0.00 | 117.61 | 0.00 |
| 312 | hsa-miR-32-3p_R-1_1ss10GA | CAATTTAGTATGTGTGATATT | Diff | 162.74 | 0.00 | 158.42 | 0.00 | 185.14 | 0.00 | 149.58 | 0.00 |
| 313 | bta-miR-2483-3p | AAACATCTGGTTGGTTGAGAGA | Yes | 161.74 | 0.00 | 183.60 | 0.00 | 258.61 | 0.00 | 249.29 | 0.00 |
| 314 | hsa-miR-26a-1-3p_1ss9TC | CCTATTCTCGGTTACTTGCACG | Diff | 159.76 | 0.00 | 143.80 | 0.00 | 172.41 | 0.00 | 163.64 | 0.00 |
| 315 | hsa-miR-532-3p | CCTCCCACACCCAAGGCTTGCA | Yes | 158.77 | 0.00 | 140.55 | 0.00 | 178.29 | 0.00 | 118.89 | 0.00 |
| 316 | bta-miR-2285aa | AAAACTGGAACGAACTTTTGGGC | Yes | 155.29 | 0.00 | 114.96 | 0.00 | 90.61 | 0.00 | 114.42 | 0.00 |
| 317 | bta-miR-2285ad_1ss8AG | AAAACCCGAATGAACTTTTTGG | Diff | 154.98 | 0.00 | 111.96 | 0.00 | 112.72 | 0.00 | 129.70 | 0.00 |
| 318 | bta-mir-2285u-p3_1ss11CT | AAAACCCGAATGAACTTTTTGG | New | 154.98 | 0.00 | 111.96 | 0.00 | 112.72 | 0.00 | 129.70 | 0.00 |
| 319 | bta-miR-2285l_R-1_1ss9CA | AAAACCCGAATGAACTTTTTGG | Diff | 154.98 | 0.00 | 111.96 | 0.00 | 112.72 | 0.00 | 129.70 | 0.00 |
| 320 | bta-miR-328_R+1 | CTGGCCCTCTCTGCCCTTCCGTT | Diff | 154.80 | 0.00 | 126.74 | 0.00 | 204.74 | 0.00 | 196.88 | 0.00 |
| 321 | bta-miR-671_R+1 | AGGAAGCCCTGGAGGGGCTGGAGG | Diff | 154.80 | 0.00 | 214.48 | 0.00 | 128.33 | 0.00 | 233.95 | 0.00 |
| 322 | bta-miR-6524_R+1 | TTACTCTGAGTAACCTAACTGT | Diff | 151.82 | 0.00 | 87.74 | 0.00 | 95.02 | 0.00 | 95.88 | 0.00 |
| 323 | bta-miR-2443 | TGAGGGCAGGACCGTATGAGGTGT | Yes | 149.84 | 0.00 | 163.29 | 0.00 | 170.45 | 0.00 | 207.10 | 0.00 |
| 324 | hsa-miR-324-3p_R+1 | ACTGCCCCAGGTGCTGCTGGT | Diff | 148.84 | 0.00 | 137.30 | 0.00 | 151.84 | 0.00 | 131.68 | 0.00 |
| 325 | hsa-miR-25-5p_R+2 | AGGCGGAGACTTGGGCAATTGCT | Diff | 147.85 | 0.00 | 176.29 | 0.00 | 229.23 | 0.00 | 269.75 | 0.00 |
| 326 | bta-mir-99b-p3 | CAAGCTCGTGTCTGTGGGTCCGT | New | 144.87 | 0.00 | 126.74 | 0.00 | 116.57 | 0.00 | 134.23 | 0.00 |
| 327 | hsa-let-7g-3p_R+1 | CTGTACAGGCCACTGCCTTGCT | Diff | 143.88 | 0.00 | 150.30 | 0.00 | 153.80 | 0.00 | 131.68 | 0.00 |
| 328 | bta-miR-1388-3p | ATCTCAGGTTTGTCAGCCCGCA | Yes | 141.90 | 0.00 | 120.24 | 0.00 | 150.86 | 0.00 | 102.27 | 0.00 |
| 329 | hsa-miR-491-3p | CTTATGCAAGATTCCCTTCTAC | Yes | 139.91 | 0.00 | 105.61 | 0.00 | 138.12 | 0.00 | 104.83 | 0.00 |
| 330 | bta-mir-2284z-6-p3_1ss8AG | GAAATCCGAACGAACTTTTTGG | New | 139.42 | 0.00 | 79.21 | 0.00 | 87.32 | 0.00 | 90.98 | 0.00 |
| 331 | bta-mir-2284aa-3-p3_1ss1AG | GAAATCCGAACGAACTTTTTGG | New | 139.42 | 0.00 | 79.21 | 0.00 | 87.32 | 0.00 | 90.98 | 0.00 |
| 332 | mml-miR-449a-3p_L+1_1ss21GA | TCAGCTAACATGCAACTGCTATC | Diff | 138.92 | 0.00 | 80.43 | 0.00 | 66.61 | 0.00 | 61.36 | 0.00 |
| 333 | PC-3p-6808_112 | AAAGCCCGAATGAACTTTTTAG | New | 138.92 | 0.00 | 115.36 | 0.00 | 83.26 | 0.00 | 103.56 | 0.00 |
| 334 | bta-miR-143_1ss22GT | TGAGATGAAGCACTGTAGCTCT | Diff | 137.93 | 0.00 | 62.56 | 0.00 | 42.12 | 0.00 | 894.90 | 0.00 |
| 335 | bta-miR-2284ab | TAAAAGTTTGGTTGGGTTTTT | Yes | 137.93 | 0.00 | 117.87 | 0.00 | 128.41 | 0.00 | 141.48 | 0.00 |
| 336 | ola-miR-222_R+6 | AGCTACATCTGGCTACTGGGTCTCC | Diff | 136.94 | 0.00 | 239.66 | 0.00 | 130.29 | 0.00 | 208.38 | 0.00 |
| 337 | bta-miR-7857_L-1 | TAGCCAGTTGGGGAAGAATGC | Diff | 135.94 | 0.00 | 121.86 | 0.00 | 116.57 | 0.00 | 166.19 | 0.00 |
| 338 | PC-3p-2321_558 | AAAGCCCAAATGAACTTTTTGT | New | 135.94 | 0.00 | 162.48 | 0.00 | 96.98 | 0.00 | 155.97 | 0.00 |
| 339 | mmu-miR-18a-3p_R-1 | ACTGCCCTAAGTGCTCCTTCT | Diff | 133.46 | 0.00 | 102.36 | 0.00 | 97.96 | 0.00 | 95.88 | 0.00 |
| 340 | efu-miR-92c_1ss12AG | AGGTTGGGATCGGTTGCAATGCT | Diff | 132.97 | 0.00 | 215.29 | 0.00 | 152.82 | 0.00 | 200.71 | 0.00 |
| 341 | bta-miR-2431-3p | CACCCCCACTTGCATGACCCTGA | Yes | 132.97 | 0.00 | 128.36 | 0.00 | 149.88 | 0.00 | 233.95 | 0.00 |
| 342 | mmu-miR-200b-5p | CATCTTACTGGGCAGCATTGGA | Yes | 131.97 | 0.00 | 301.40 | 0.00 | 327.19 | 0.00 | 311.94 | 0.00 |
| 343 | bta-miR-664b_R+1 | TATTCATTTATCTCCCAGCCTACA | Diff | 131.97 | 0.00 | 391.58 | 0.00 | 277.23 | 0.00 | 336.23 | 0.00 |
| 344 | chi-miR-3432-3p | CAGCAACTAAAGATCCCTCAGG | Yes | 131.97 | 0.00 | 95.86 | 0.00 | 93.06 | 0.00 | 98.44 | 0.00 |
| 345 | bta-miR-1260b | ATCCCACCACTGCCACCA | Yes | 130.98 | 0.00 | 98.30 | 0.00 | 141.06 | 0.00 | 121.45 | 0.00 |
| 346 | hsa-miR-671-3p | TCCGGTTCTCAGGGCTCCACC | Yes | 129.99 | 0.00 | 90.18 | 0.00 | 139.10 | 0.00 | 144.46 | 0.00 |
| 347 | bta-miR-199a-5p_R+1 | CCCAGTGTTCAGACTACCTGTTC | Diff | 129.98 | 0.00 | 76.64 | 0.00 | 62.36 | 0.00 | 73.30 | 0.00 |
| 348 | bta-miR-877_R+3 | GTAGAGGAGATGGCGCAGGGGAC | Diff | 129.00 | 0.00 | 147.05 | 0.00 | 195.92 | 0.00 | 246.74 | 0.00 |
| 349 | dre-miR-30e-5p_R+2_1ss13TC | TGTAAACATCCTCGACTGGAAGCC | Diff | 123.54 | 0.00 | 264.03 | 0.00 | 173.88 | 0.00 | 272.30 | 0.00 |
| 350 | ccr-miR-17-5p_R+2 | CAAAGTGCTTACAGTGCAGGTAGAA | Diff | 123.04 | 0.00 | 470.38 | 0.00 | 76.41 | 0.00 | 352.84 | 0.00 |
| 351 | bta-miR-2284v_R+2_1ss17GT | AAAAAGTTCGTTTGGGTTTTCT | Diff | 122.54 | 0.00 | 93.88 | 0.00 | 111.67 | 0.00 | 94.76 | 0.00 |
| 352 | bta-miR-2313-3p_R+1 | CCAGTTCCACGCTGCATGCCT | Diff | 121.06 | 0.00 | 69.05 | 0.00 | 89.14 | 0.00 | 80.54 | 0.00 |
| 353 | hsa-miR-574-5p_R+2 | TGAGTGTGTGTGTGTGAGTGTGTGT | Diff | 120.40 | 0.00 | 187.94 | 0.00 | 160.66 | 0.00 | 294.88 | 0.00 |
| 354 | bta-mir-2284d-p3_1ss16CT | AAAACTCGAACAAACTTTTTGT | New | 120.07 | 0.00 | 60.93 | 0.00 | 81.31 | 0.00 | 100.36 | 0.00 |
| 355 | bta-miR-6122-3p | ACTATATGATGTAAACTGAAC | Yes | 112.13 | 0.00 | 112.11 | 0.00 | 110.69 | 0.00 | 67.76 | 0.00 |
| 356 | mmu-miR-30c-5p_R+1 | TGTAAACATCCTACACTCTCAGCG | Diff | 112.13 | 0.00 | 97.49 | 0.00 | 96.98 | 0.00 | 230.12 | 0.00 |
| 357 | bta-miR-2285ac | AAAACCTGAAGAGACTTTTTGG | Yes | 110.64 | 0.00 | 64.99 | 0.00 | 61.71 | 0.00 | 74.79 | 0.00 |
| 358 | hsa-miR-193b-5p | CGGGGTTTTGAGGGCGAGATGA | Yes | 110.14 | 0.00 | 112.11 | 0.00 | 72.49 | 0.00 | 111.22 | 0.00 |
| 359 | ssc-miR-9851-3p | TGGCACCAGCACTGGCGGTGGC | Yes | 110.14 | 0.00 | 104.80 | 0.00 | 96.00 | 0.00 | 81.82 | 0.00 |
| 360 | bta-miR-2425-5p | CCAGGGCACGGATCCATGAACT | Yes | 107.17 | 0.00 | 53.62 | 0.00 | 43.10 | 0.00 | 63.92 | 0.00 |
| 361 | mmu-let-7e-3p | CTATACGGCCTCCTAGCTTTCC | Yes | 106.17 | 0.00 | 85.30 | 0.00 | 91.10 | 0.00 | 79.26 | 0.00 |
| 362 | hsa-miR-181a-2-3p_1ss6TC | ACCACCGACCGTTGACTGTACC | Diff | 106.17 | 0.00 | 74.74 | 0.00 | 88.16 | 0.00 | 69.67 | 0.00 |
| 363 | hsa-miR-188-3p | CTCCCACATGCAGGGTTTGCA | Yes | 105.18 | 0.00 | 51.18 | 0.00 | 68.57 | 0.00 | 53.69 | 0.00 |
| 364 | bta-miR-204 | TTCCCTTTGTCATCCTATGCCT | Yes | 104.19 | 0.00 | 192.54 | 0.00 | 164.08 | 0.00 | 152.13 | 0.00 |
| 365 | bta-miR-2355-5p_L+2R-2 | TTCCCAGATACAATGGACAATAT | Diff | 104.19 | 0.00 | 55.24 | 0.00 | 51.92 | 0.00 | 44.74 | 0.00 |
| 366 | bta-mir-2285l-p5 | CATAGTTCATTCGAGTTTTTC | New | 103.20 | 0.00 | 78.80 | 0.00 | 81.31 | 0.00 | 80.54 | 0.00 |
| 367 | ssa-miR-10d-5p_R+4_1ss1CT | TACCCTGTAGAACCGAATTTGTGTTT | Diff | 100.22 | 0.00 | 452.92 | 0.00 | 103.84 | 0.00 | 283.17 | 0.00 |
| 368 | mmu-miR-1839-3p | AGACCTACTTATCTACCAACAGC | Yes | 98.24 | 0.00 | 220.16 | 0.00 | 224.33 | 0.00 | 227.56 | 0.00 |
| 369 | bta-mir-7857-p3 | TTTGTTCTCCAACCTGGCTCTTT | New | 97.24 | 0.00 | 52.81 | 0.00 | 46.04 | 0.00 | 63.92 | 0.00 |
| 370 | bta-miR-449c_R+5 | AGGCAGTGCATCTCTAGCTGGCTGTT | Diff | 95.26 | 0.00 | 107.24 | 0.00 | 43.10 | 0.00 | 79.26 | 0.00 |
| 371 | hsa-miR-802_L+1R-2 | TCAGTAACAAAGATTCATCCTT | Diff | 95.26 | 0.00 | 26.81 | 0.00 | 28.41 | 0.00 | 21.73 | 0.00 |
| 372 | bta-miR-199a-3p_R-1 | ACAGTAGTCTGCACATTGGTT | Diff | 93.78 | 0.00 | 119.01 | 0.00 | 128.58 | 0.00 | 108.03 | 0.00 |
| 373 | bta-miR-129-5p_R-1 | CTTTTTGCGGTCTGGGCTTGC | Diff | 92.32 | 0.00 | 75.56 | 0.00 | 94.04 | 0.00 | 66.48 | 0.00 |
| 374 | rno-miR-191b_R+2_1ss15TC | GAACGAAATCCAAGCGCAGCTGT | Diff | 91.29 | 0.00 | 68.24 | 0.00 | 52.90 | 0.00 | 70.31 | 0.00 |
| 375 | PC-5p-2352_548 | GAAAGTTTGTTGGGGTTTTTCT | New | 91.29 | 0.00 | 216.10 | 0.00 | 101.88 | 0.00 | 186.01 | 0.00 |
| 376 | PC-3p-14474_37 | TATGAAGTCCTTAAGGGGAGGG | New | 90.30 | 0.00 | 54.44 | 0.00 | 65.64 | 0.00 | 48.58 | 0.00 |
| 377 | hsa-miR-100-3p_R-1_1ss10AG | CAAGCTTGTGTCTATAGGTAT | Diff | 90.30 | 0.00 | 50.37 | 0.00 | 64.65 | 0.00 | 61.36 | 0.00 |
| 378 | bta-miR-2285i_L-1R+1 | AAACCGGAACGAACTTTTTGGT | Diff | 87.54 | 0.00 | 128.06 | 0.00 | 100.85 | 0.00 | 132.85 | 0.00 |
| 379 | PC-3p-7967_88 | CCCAAGTGAACTTGTTGGCC | New | 87.32 | 0.00 | 32.90 | 0.00 | 43.59 | 0.00 | 41.55 | 0.00 |
| 380 | aca-miR-128-3p_R+2 | TCACAGTGAACCGGTCTCTTTTT | Diff | 86.33 | 0.00 | 185.23 | 0.00 | 106.78 | 0.00 | 209.66 | 0.00 |
| 381 | bta-miR-2285o_R+2 | AAACCCGAACGAACTTTTGGGC | Diff | 84.56 | 0.00 | 63.08 | 0.00 | 69.00 | 0.00 | 41.00 | 0.00 |
| 382 | PC-3p-4602_203 | TCGAACAAACTTTTTGGCCAAC | New | 82.86 | 0.00 | 44.28 | 0.00 | 64.16 | 0.00 | 77.98 | 0.00 |
| 383 | hsa-miR-365b-5p_R+1 | AGGGACTTTCAGGGGCAGCTGTG | Diff | 82.36 | 0.00 | 120.24 | 0.00 | 95.02 | 0.00 | 129.12 | 0.00 |
| 384 | PC-5p-7634_94 | CAAAAGCTCATTCAGGTTTTT | New | 82.36 | 0.00 | 82.46 | 0.00 | 103.35 | 0.00 | 62.00 | 0.00 |
| 385 | bta-miR-582_L-1 | TACAGTTGTTCAACCAGTTACT | Diff | 81.37 | 0.00 | 44.68 | 0.00 | 45.06 | 0.00 | 45.38 | 0.00 |
| 386 | hsa-miR-582-5p_L-1 | TACAGTTGTTCAACCAGTTACT | Diff | 81.37 | 0.00 | 44.68 | 0.00 | 45.06 | 0.00 | 45.38 | 0.00 |
| 387 | bta-miR-499 | TTAAGACTTGCAGTGATGTTT | Yes | 79.38 | 0.00 | 140.55 | 0.00 | 157.72 | 0.00 | 112.50 | 0.00 |
| 388 | ssa-miR-10a-5p | TACCCTGTAGATCCGGATTTGT | Yes | 79.38 | 0.00 | 47.12 | 0.00 | 63.67 | 0.00 | 51.14 | 0.00 |
| 389 | mml-miR-130a-5p | GCTCTTTTCACATTGTGCTACT | Yes | 78.39 | 0.00 | 55.24 | 0.00 | 60.74 | 0.00 | 39.63 | 0.00 |
| 390 | hsa-miR-744-3p_R-1 | CTGTTGCCACTAACCTCAACC | Diff | 78.39 | 0.00 | 110.49 | 0.00 | 121.47 | 0.00 | 101.00 | 0.00 |
| 391 | PC-3p-6776_112 | TGCTATCTCTATAAGTATCAGA | New | 78.39 | 0.00 | 26.81 | 0.00 | 37.22 | 0.00 | 24.29 | 0.00 |
| 392 | bta-miR-138_R+1 | AGCTGGTGTTGTGAATCAGGCCGT | Diff | 76.90 | 0.00 | 31.68 | 0.00 | 21.55 | 0.00 | 26.85 | 0.00 |
| 393 | mmu-miR-19b-1-5p | AGTTTTGCAGGTTTGCATCCAGC | Yes | 75.41 | 0.00 | 127.55 | 0.00 | 73.47 | 0.00 | 61.36 | 0.00 |
| 394 | mmu-miR-128-1-5p_R+2 | CGGGGCCGTAGCACTGTCTGAGA | Diff | 74.42 | 0.00 | 98.30 | 0.00 | 74.45 | 0.00 | 101.00 | 0.00 |
| 395 | pma-miR-30a-5p_R+2 | TGTAAACATCCTCGACTGGAAGCCT | Diff | 74.42 | 0.00 | 182.39 | 0.00 | 85.22 | 0.00 | 153.41 | 0.00 |
| 396 | PC-5p-6623_116 | ATGCCTTCCCCAGCCTCCGAGC | New | 74.42 | 0.00 | 43.06 | 0.00 | 55.84 | 0.00 | 33.24 | 0.00 |
| 397 | hsa-miR-1277-5p_L-3R+2 | TATATATATATATGTACGTATGA | Diff | 74.42 | 0.00 | 152.74 | 0.00 | 96.98 | 0.00 | 76.70 | 0.00 |
| 398 | PC-3p-4980_178 | CCCCCACTTGCATGACCCTGAGT | New | 73.43 | 0.00 | 95.05 | 0.00 | 64.65 | 0.00 | 94.60 | 0.00 |
| 399 | bta-miR-129-3p | AAGCCCTTACCCCAAAAAGCAT | Yes | 72.93 | 0.00 | 73.12 | 0.00 | 53.88 | 0.00 | 65.20 | 0.00 |
| 400 | mmu-miR-29b-1-5p_R+2 | GCTGGTTTCATATGGTGGTTTAGA | Diff | 72.44 | 0.00 | 125.11 | 0.00 | 53.88 | 0.00 | 145.74 | 0.00 |
| 401 | hsa-miR-301a-5p | GCTCTGACTTTATTGCACTACT | Yes | 72.44 | 0.00 | 76.37 | 0.00 | 100.90 | 0.00 | 132.96 | 0.00 |
| 402 | sha-miR-125a_R+2_2 | TCCCTGAGACCCTAACTTGTGAGT | Diff | 71.94 | 0.00 | 75.15 | 0.00 | 47.02 | 0.00 | 115.70 | 0.00 |
| 403 | sha-miR-125a_R+2_1 | TCCCTGAGACCCTAACTTGTGAAA | Diff | 71.94 | 0.00 | 75.15 | 0.00 | 47.02 | 0.00 | 115.70 | 0.00 |
| 404 | bta-miR-2285m_L+1R-1_1ss7GA | AAAAACACAAATGAACTTTTTG | Diff | 71.24 | 0.00 | 26.70 | 0.00 | 24.14 | 0.00 | 20.40 | 0.00 |
| 405 | bta-miR-2284aa | AAAAAAGTTTGTTTGGGTTTT | Yes | 71.10 | 0.00 | 59.94 | 0.00 | 84.88 | 0.00 | 63.38 | 0.00 |
| 406 | bta-miR-2285v_R+1 | AGAACCGGAACGAACTTTTTGG | Diff | 70.75 | 0.00 | 51.83 | 0.00 | 43.30 | 0.00 | 68.40 | 0.00 |
| 407 | hsa-miR-30c-1-3p | CTGGGAGAGGGTTGTTTACTCC | Yes | 70.45 | 0.00 | 64.99 | 0.00 | 57.80 | 0.00 | 63.92 | 0.00 |
| 408 | bta-miR-147_R-1 | GTGTGCGGAAATGCTTCTGCT | Diff | 70.45 | 0.00 | 57.68 | 0.00 | 96.98 | 0.00 | 74.15 | 0.00 |
| 409 | bta-miR-2431-5p | CAGGTCATATAAGTGTGGAGTT | Yes | 69.46 | 0.00 | 138.92 | 0.00 | 98.94 | 0.00 | 108.67 | 0.00 |
| 410 | PC-5p-4828_187 | TAAAAAGTTCGTTTGGGTTTTC | New | 67.48 | 0.00 | 40.62 | 0.00 | 52.90 | 0.00 | 43.46 | 0.00 |
| 411 | hsa-miR-183-3p_L-1R+1 | TGAATTACCGAAGGGCCATAAT | Diff | 67.48 | 0.00 | 69.05 | 0.00 | 100.90 | 0.00 | 111.22 | 0.00 |
| 412 | PC-3p-7986_88 | AAAACTTGAATGAACCTTTTGG | New | 66.48 | 0.00 | 42.65 | 0.00 | 35.76 | 0.00 | 48.58 | 0.00 |
| 413 | hsa-miR-155-3p_1ss10AG | CTCCTACATGTTAGCATTAACA | Diff | 65.49 | 0.00 | 66.62 | 0.00 | 57.80 | 0.00 | 48.58 | 0.00 |
| 414 | cin-miR-184_R+1_1ss22CT | TGGACGGAGAACTGATAAGGGTT | Diff | 65.49 | 0.00 | 52.81 | 0.00 | 55.84 | 0.00 | 81.82 | 0.00 |
| 415 | bta-miR-199b_2ss10TC17TC | CCCAGTGTTCAGACTACCTGTTC | Diff | 64.99 | 0.00 | 38.32 | 0.00 | 31.18 | 0.00 | 36.65 | 0.00 |
| 416 | bta-miR-133a_L-1R+1 | TTGGTCCCCTTCAACCAGCTGT | Diff | 64.50 | 0.00 | 7.32 | 0.00 | 3.92 | 0.00 | 0 | 0.00 |
| 417 | hsa-miR-1249-5p_1ss21AC | AGGAGGGAGGAGATGGGCCACGTT | Diff | 63.51 | 0.00 | 43.06 | 0.00 | 48.98 | 0.00 | 72.87 | 0.00 |
| 418 | bta-mir-2284e-p3_1ss16AT | AAAACCTGAACAAACTTTTTGG | New | 63.19 | 0.00 | 39.97 | 0.00 | 40.20 | 0.00 | 68.53 | 0.00 |
| 419 | bta-miR-2285c_L+1R-1 | AAAACCTGAACAAACTTTTTGG | Diff | 63.19 | 0.00 | 39.97 | 0.00 | 40.20 | 0.00 | 68.53 | 0.00 |
| 420 | PC-3p-4848_186 | AAACTCAAATGAACTTTTTGGT | New | 62.51 | 0.00 | 51.18 | 0.00 | 27.43 | 0.00 | 42.19 | 0.00 |
| 421 | hsa-miR-210-5p_R+1_1ss5CA | AGCCACTGCCCACCGCACACTGC | Diff | 61.52 | 0.00 | 67.43 | 0.00 | 92.08 | 0.00 | 95.88 | 0.00 |
| 422 | mmu-miR-6240_L-1R-6 | CAAAGCATCGCGAAGGCCC | Diff | 61.52 | 0.00 | 82.05 | 0.00 | 103.84 | 0.00 | 124.01 | 0.00 |
| 423 | PC-3p-4053_245 | AAAACTCGAGCAAACTTTTTGGT | New | 61.03 | 0.00 | 61.34 | 0.00 | 47.51 | 0.00 | 57.53 | 0.00 |
| 424 | bta-miR-2299-3p_L+1 | GTCCGGGGAATGGATCCAGCGT | Diff | 60.53 | 0.00 | 63.37 | 0.00 | 73.47 | 0.00 | 62.64 | 0.00 |
| 425 | hsa-miR-454-5p_1ss9AG | ACCCTATCGATATTGTCTCTGC | Diff | 59.54 | 0.00 | 46.31 | 0.00 | 43.10 | 0.00 | 40.91 | 0.00 |
| 426 | bta-miR-1842_L+1 | TTGGCTCTGTGAGGTCGGCTCA | Diff | 59.54 | 0.00 | 30.06 | 0.00 | 60.74 | 0.00 | 47.30 | 0.00 |
| 427 | aca-miR-21-5p_L+2R+1 | AATAGCTTATCAGACTGATGTTGAC | Diff | 58.55 | 0.00 | 78.80 | 0.00 | 67.59 | 0.00 | 178.98 | 0.00 |
| 428 | PC-3p-7173_104 | AAAACCTGAATGAACCTTTTGT | New | 58.55 | 0.00 | 43.06 | 0.00 | 45.06 | 0.00 | 39.63 | 0.00 |
| 429 | bta-miR-146b | TGAGAACTGAATTCCATAGGCTGT | Yes | 55.57 | 0.00 | 35.34 | 0.00 | 33.31 | 0.00 | 79.26 | 0.00 |
| 430 | bta-miR-2399-3p | TTCTAACAACTACAGAAAGTGT | Yes | 55.57 | 0.00 | 26.00 | 0.00 | 32.33 | 0.00 | 29.40 | 0.00 |
| 431 | efu-miR-16_R+1_1ss21CT | TAGCAGCACGTAAATATTGGTGTA | Diff | 55.57 | 0.00 | 75.55 | 0.00 | 73.47 | 0.00 | 85.65 | 0.00 |
| 432 | oan-miR-16b-5p_R+2_2 | TAGCAGCACGTAAATATTGGTGTA | Diff | 55.57 | 0.00 | 75.55 | 0.00 | 73.47 | 0.00 | 85.65 | 0.00 |
| 433 | aca-miR-16a-5p_R+4 | TAGCAGCACGTAAATATTGGTGAA | Diff | 55.57 | 0.00 | 75.55 | 0.00 | 73.47 | 0.00 | 85.65 | 0.00 |
| 434 | oan-miR-16b-5p_R+2_1 | TAGCAGCACGTAAATATTGGTGAA | Diff | 55.57 | 0.00 | 75.55 | 0.00 | 73.47 | 0.00 | 85.65 | 0.00 |
| 435 | aca-miR-99b-5p_R-1 | AACCCGTAGATCCGAACTTGCG | Diff | 54.58 | 0.00 | 17.87 | 0.00 | 28.41 | 0.00 | 16.62 | 0.00 |
| 436 | PC-3p-7041_107 | ACACGCGTCCTTGGATCCTGACT | New | 53.58 | 0.00 | 47.12 | 0.00 | 47.02 | 0.00 | 46.02 | 0.00 |
| 437 | mmu-miR-182-3p_L+1 | GGTGGTTCTAGACTTGCCAACT | Diff | 52.59 | 0.00 | 69.87 | 0.00 | 87.18 | 0.00 | 53.69 | 0.00 |
| 438 | hsa-miR-200a-5p | CATCTTACCGGACAGTGCTGGA | Yes | 52.59 | 0.00 | 77.18 | 0.00 | 92.08 | 0.00 | 99.72 | 0.00 |
| 439 | PC-3p-12036_48 | TAAAACCCGAGCTCCTTGTTAG | New | 52.59 | 0.00 | 24.37 | 0.00 | 23.51 | 0.00 | 34.52 | 0.00 |
| 440 | hsa-miR-219a-5p_R+2 | TGATTGTCCAAACGCAATTCTCG | Diff | 52.10 | 0.00 | 36.15 | 0.00 | 41.14 | 0.00 | 19.82 | 0.00 |
| 441 | hsa-miR-652-5p_L+1R-2 | ACAACCCTAGGAGAGGGTGCCATT | Diff | 52.10 | 0.00 | 74.74 | 0.00 | 42.61 | 0.00 | 43.47 | 0.00 |
| 442 | cgr-miR-652-5p_L+1R-2 | ACAACCCTAGGAGAGGGTGCCATT | Diff | 52.10 | 0.00 | 74.74 | 0.00 | 42.61 | 0.00 | 43.47 | 0.00 |
| 443 | fru-miR-29a_R+2 | TAGCACCATTTGAAATCGGTTACT | Diff | 51.60 | 0.00 | 101.55 | 0.00 | 101.88 | 0.00 | 79.26 | 0.00 |
| 444 | ggo-let-7a_R+1_1ss16TG | TGAGGTAGTAGGTTGGATAGTT | Diff | 50.11 | 0.00 | 61.74 | 0.00 | 78.86 | 0.00 | 88.21 | 0.00 |
| 445 | ssa-let-7j-5p | TGAGGTAGTAGGTTGGATAGTT | Yes | 50.11 | 0.00 | 61.74 | 0.00 | 78.86 | 0.00 | 88.21 | 0.00 |
| 446 | bta-miR-545-5p_R+1 | TCAGTAAATGTTTATTGGATGA | Diff | 49.61 | 0.00 | 31.68 | 0.00 | 36.25 | 0.00 | 33.24 | 0.00 |
| 447 | mdo-miR-24-3p_L+3R+4 | ATCTGGCTCAGTTCAGCAGGAAC | Diff | 49.61 | 0.00 | 45.49 | 0.00 | 41.63 | 0.00 | 46.02 | 0.00 |
| 448 | ola-miR-222_R+7 | AGCTACATCTGGCTACTGGGTCTCCT | Diff | 49.61 | 0.00 | 80.43 | 0.00 | 15.67 | 0.00 | 72.87 | 0.00 |
| 449 | ssa-miR-30d-2-3p_2ss1TC23TA | CTTTCAGTCGGATGTTTGCAGCA | Diff | 48.62 | 0.00 | 108.86 | 0.00 | 93.06 | 0.00 | 104.83 | 0.00 |
| 450 | hsa-miR-101-5p_L+1R-1 | TCAGTTATCACAGTGCTGATGC | Diff | 47.63 | 0.00 | 74.74 | 0.00 | 73.47 | 0.00 | 38.35 | 0.00 |
| 451 | hsa-miR-135a-3p_L+1R-1 | ATATAGGGATTGGAGCCGTGGC | Diff | 47.63 | 0.00 | 56.87 | 0.00 | 83.27 | 0.00 | 101.00 | 0.00 |
| 452 | hsa-miR-505-5p_R+2 | GGGAGCCAGGAAGTATTGATGTTT | Diff | 47.63 | 0.00 | 41.43 | 0.00 | 38.20 | 0.00 | 76.71 | 0.00 |
| 453 | PC-3p-15288_34 | AAAACCCGAAGGAACTTTTTTGG | New | 46.64 | 0.00 | 35.74 | 0.00 | 17.64 | 0.00 | 29.40 | 0.00 |
| 454 | hsa-miR-23a-5p | GGGGTTCCTGGGGATGGGATTT | Yes | 46.64 | 0.00 | 30.87 | 0.00 | 35.27 | 0.00 | 58.81 | 0.00 |
| 455 | bta-miR-760-3p_R+2 | CGGCTCTGGGTCTGTGGGGAGT | Diff | 46.64 | 0.00 | 46.31 | 0.00 | 53.88 | 0.00 | 60.09 | 0.00 |
| 456 | cgr-miR-486-5p_R+2 | TCCTGTACTGAGCTGCCCCGAGTT | Diff | 46.64 | 0.00 | 153.55 | 0.00 | 81.31 | 0.00 | 147.02 | 0.00 |
| 457 | PC-3p-10719_57 | AGGAACCCAGATGAACTTTCT | New | 46.64 | 0.00 | 18.69 | 0.00 | 19.59 | 0.00 | 20.45 | 0.00 |
| 458 | PC-3p-7811_91 | AAAAACTAGAATGAACTTTTTGT | New | 46.64 | 0.00 | 34.12 | 0.00 | 20.57 | 0.00 | 19.18 | 0.00 |
| 459 | bta-mir-2285t-p5 | AAAAGTTCGTTCAGGTTTTTCT | New | 44.77 | 0.00 | 76.43 | 0.00 | 100.41 | 0.00 | 87.68 | 0.00 |
| 460 | hsa-miR-450a-2-3p_L+1R-1_1ss8GA | TATTGGGAACATTTTGCATTCA | Diff | 43.66 | 0.00 | 43.87 | 0.00 | 54.86 | 0.00 | 26.85 | 0.00 |
| 461 | bta-miR-1814c_R-2 | GTTTTGTTTGGGTTTGTT | Diff | 43.66 | 0.00 | 37.78 | 0.00 | 68.08 | 0.00 | 60.30 | 0.00 |
| 462 | bta-miR-2284t-5p_L+1 | CGAAACATTCACTCGGGTTTTT | Diff | 43.66 | 0.00 | 42.25 | 0.00 | 36.25 | 0.00 | 31.96 | 0.00 |
| 463 | PC-3p-12416_46 | AAAACCCGACCGAACTTTTCTGGT | New | 43.66 | 0.00 | 49.15 | 0.00 | 40.16 | 0.00 | 57.53 | 0.00 |
| 464 | PC-3p-12034_48 | CAAACCCAAGAGAACTTTTTAGT | New | 43.16 | 0.00 | 23.56 | 0.00 | 16.16 | 0.00 | 26.21 | 0.00 |
| 465 | hsa-miR-96-3p_L+1R-1 | CAATCATGTGCAGTGCCAATAT | Diff | 42.67 | 0.00 | 26.81 | 0.00 | 30.37 | 0.00 | 23.01 | 0.00 |
| 466 | PC-3p-7623_94 | AAAACCCTGATGAACTTTTTGA | New | 41.68 | 0.00 | 21.12 | 0.00 | 19.60 | 0.00 | 29.40 | 0.00 |
| 467 | PC-3p-10629_57 | AAACCCAGAGTAAACTTTTTAG | New | 40.68 | 0.00 | 17.06 | 0.00 | 9.80 | 0.00 | 7.67 | 0.00 |
| 468 | PC-3p-9384_69 | AAAACTAGAATGAACTTTTTGG | New | 40.19 | 0.00 | 17.47 | 0.00 | 24.00 | 0.00 | 31.96 | 0.00 |
| 469 | bta-miR-503-3p | GGAGTATTGTTTCTGCTGCCCGG | Yes | 39.69 | 0.00 | 51.99 | 0.00 | 59.76 | 0.00 | 48.58 | 0.00 |
| 470 | ptr-miR-628_R+3 | TCTAGTAAGAGTGGCAGTCGAAGT | Diff | 39.69 | 0.00 | 21.12 | 0.00 | 26.45 | 0.00 | 31.96 | 0.00 |
| 471 | hsa-miR-6516-3p | ATCATGTATGATACTGCAAACA | Yes | 39.69 | 0.00 | 81.24 | 0.00 | 100.90 | 0.00 | 67.76 | 0.00 |
| 472 | mdo-miR-22-3p | AAGCTGCCAGTTGAAGAACTGC | Yes | 39.69 | 0.00 | 59.31 | 0.00 | 62.69 | 0.00 | 53.69 | 0.00 |
| 473 | oha-let-7c-5p_R+2_1ss17AG | TGAGGTAGTAGGTTGTGTGGTTGA | Diff | 39.69 | 0.00 | 31.28 | 0.00 | 33.31 | 0.00 | 49.86 | 0.00 |
| 474 | PC-3p-7097_106 | AAAACCGGAATGAACTTTTTTGG | New | 39.69 | 0.00 | 26.00 | 0.00 | 19.59 | 0.00 | 37.07 | 0.00 |
| 475 | bta-miR-2285t | AGAATCTGGATGAACTTTTTGG | Yes | 39.61 | 0.00 | 62.76 | 0.00 | 35.46 | 0.00 | 65.88 | 0.00 |
| 476 | bta-mir-2285k-5-p5 | AGAAAGTTCATTCAGGTTTTT | New | 39.34 | 0.00 | 42.58 | 0.00 | 54.07 | 0.00 | 32.28 | 0.00 |
| 477 | bta-miR-138 | AGCTGGTGTTGTGAATCAGGCCG | Yes | 39.20 | 0.00 | 16.25 | 0.00 | 13.71 | 0.00 | 10.23 | 0.00 |
| 478 | bta-mir-2285q-p5 | AAAAAGTTCATTCAGGTTTTCT | New | 38.78 | 0.00 | 46.17 | 0.00 | 34.86 | 0.00 | 15.87 | 0.00 |
| 479 | mmu-miR-185-3p_2ss18TC21TC | AGGGGCTGGCTTTCCTCCGGC | Diff | 38.70 | 0.00 | 13.81 | 0.00 | 24.49 | 0.00 | 17.90 | 0.00 |
| 480 | bta-miR-301b_R-2 | CAGTGCAATGATATTGTCAAAGC | Diff | 38.70 | 0.00 | 22.75 | 0.00 | 34.29 | 0.00 | 23.01 | 0.00 |
| 481 | hsa-miR-449b-3p_L-1_1ss12AG | AGCCACAACTGCCCTGCCACT | Diff | 38.70 | 0.00 | 21.12 | 0.00 | 16.65 | 0.00 | 19.18 | 0.00 |
| 482 | PC-3p-6258_127 | AAACTCGAACGAACTTTTTAGC | New | 38.20 | 0.00 | 22.75 | 0.00 | 22.53 | 0.00 | 32.60 | 0.00 |
| 483 | bta-mir-7865-p3_1ss21GT | CTGACTGCCGCCCCCCGCAGT | New | 37.71 | 0.00 | 44.68 | 0.00 | 62.69 | 0.00 | 42.19 | 0.00 |
| 484 | eca-miR-885-3p | AGGCAGCGGGGTGTAGTGGATA | Yes | 37.21 | 0.00 | 47.93 | 0.00 | 69.55 | 0.00 | 72.87 | 0.00 |
| 485 | hsa-miR-885-3p | AGGCAGCGGGGTGTAGTGGATA | Yes | 37.21 | 0.00 | 47.93 | 0.00 | 69.55 | 0.00 | 72.87 | 0.00 |
| 486 | bta-miR-365-5p | AGGGACTTTTGGGGGCAGATGTG | Yes | 36.71 | 0.00 | 61.74 | 0.00 | 19.59 | 0.00 | 37.07 | 0.00 |
| 487 | bta-mir-1468-p3 | TGTTCATTTGACTTATTCTC | New | 36.71 | 0.00 | 21.12 | 0.00 | 39.18 | 0.00 | 25.57 | 0.00 |
| 488 | bta-mir-450a-1-p3 | ATTGGGAGCATTTTGCATGCAT | New | 35.72 | 0.00 | 38.18 | 0.00 | 30.37 | 0.00 | 21.73 | 0.00 |
| 489 | bta-miR-502b_R+1 | AATCCACCTGGGCAAGGATTCT | Diff | 35.72 | 0.00 | 43.87 | 0.00 | 19.59 | 0.00 | 21.09 | 0.00 |
| 490 | bta-miR-2367-3p_R+1 | TTGAAAGGCACTTACAGGAAGC | Diff | 35.72 | 0.00 | 26.00 | 0.00 | 30.37 | 0.00 | 25.57 | 0.00 |
| 491 | mmu-miR-1949_L-1_1ss14TC | TATACCAGGATGCCAGCATAGTT | Diff | 35.72 | 0.00 | 43.87 | 0.00 | 36.25 | 0.00 | 38.35 | 0.00 |
| 492 | pol-let-7a-5p_R+3_1ss17AG | TGAGGTAGTAGGTTGTGTGGTTTGT | Diff | 35.72 | 0.00 | 120.30 | 0.00 | 45.06 | 0.00 | 101.00 | 0.00 |
| 493 | bta-mir-2285h-p5_1ss22CT | AAAAGTTCATTCAGGTTTTTCT | New | 35.06 | 0.00 | 31.48 | 0.00 | 34.20 | 0.00 | 21.95 | 0.00 |
| 494 | bta-mir-2285g-3-p5_1ss22AT | AAAAGTTCATTCAGGTTTTTCT | New | 35.06 | 0.00 | 31.48 | 0.00 | 34.20 | 0.00 | 21.95 | 0.00 |
| 495 | hsa-miR-203a-3p_L-1R+1 | TGAAATGTTTAGGACCACTAGT | Diff | 34.74 | 0.00 | 35.74 | 0.00 | 32.32 | 0.00 | 61.36 | 0.00 |
| 496 | bta-miR-139 | TCTACAGTGCACGTGTCTCCAGT | Yes | 34.73 | 0.00 | 44.68 | 0.00 | 64.65 | 0.00 | 46.02 | 0.00 |
| 497 | mml-miR-7180-3p | TGGCCTCTGGGTGTGTACCCT | Yes | 34.73 | 0.00 | 34.12 | 0.00 | 39.18 | 0.00 | 39.63 | 0.00 |
| 498 | dre-miR-107b_R-1 | AGCAGCATTGTACAGGGCTT | Diff | 34.73 | 0.00 | 37.37 | 0.00 | 64.65 | 0.00 | 48.58 | 0.00 |
| 499 | PC-5p-16045_32 | AGTTGAGGCTCCGAGCTTCGGT | New | 34.73 | 0.00 | 17.87 | 0.00 | 33.31 | 0.00 | 17.90 | 0.00 |
| 500 | PC-5p-6684_115 | CCAAAGTATGTTCCAGCT | New | 34.73 | 0.00 | 19.50 | 0.00 | 41.14 | 0.00 | 24.29 | 0.00 |
| 501 | hsa-miR-103a-2-5p_R+1 | AGCTTCTTTACAGTGCTGCCTTGT | Diff | 34.23 | 0.00 | 28.03 | 0.00 | 33.31 | 0.00 | 28.76 | 0.00 |
| 502 | mmu-miR-107-5p_R+1_1ss16TC | AGCTTCTTTACAGTGCTGCCTTGT | Diff | 34.23 | 0.00 | 28.03 | 0.00 | 33.31 | 0.00 | 28.76 | 0.00 |
| 503 | bta-miR-190b_R+1 | TGATATGTTTGATATTGGGTTG | Diff | 33.74 | 0.00 | 29.25 | 0.00 | 25.47 | 0.00 | 28.13 | 0.00 |
| 504 | hsa-miR-335-3p | TTTTTCATTATTGCTCCTGACC | Yes | 33.74 | 0.00 | 20.31 | 0.00 | 14.69 | 0.00 | 17.90 | 0.00 |
| 505 | PC-3p-9203_71 | CTCCCTCGGAACCCGGCTGGGACT | New | 33.74 | 0.00 | 25.18 | 0.00 | 13.71 | 0.00 | 49.86 | 0.00 |
| 506 | bta-miR-2285n_L-1_1ss21GT | AAAACCCGAATGAACTTTTTG | Diff | 33.60 | 0.00 | 18.32 | 0.00 | 22.16 | 0.00 | 28.00 | 0.00 |
| 507 | bta-miR-2285aa_1ss23CT | AAAACTGGAACGAACTTTTGGGT | Diff | 32.75 | 0.00 | 26.81 | 0.00 | 17.63 | 0.00 | 21.73 | 0.00 |
| 508 | bta-miR-2320-3p | TCGATGATGGTCCCTGTGTTTT | Yes | 32.75 | 0.00 | 32.50 | 0.00 | 27.43 | 0.00 | 34.52 | 0.00 |
| 509 | bta-miR-7862 | TGGTGCTCCCTGGAGCTGAGC | Yes | 32.75 | 0.00 | 21.94 | 0.00 | 28.41 | 0.00 | 24.29 | 0.00 |
| 510 | hsa-miR-342-5p_R+5_1ss17AG | AGGGGTGCTATCTGTGGTTGAGGACA | Diff | 31.75 | 0.00 | 109.68 | 0.00 | 32.33 | 0.00 | 94.60 | 0.00 |
| 511 | PC-5p-22686_20 | CCAAGACCATACATTAGGTGTA | New | 31.75 | 0.00 | 12.19 | 0.00 | 13.71 | 0.00 | 10.23 | 0.00 |
| 512 | PC-5p-9764_65 | AAAGGTTCGTTCGGGTTTTCCT | New | 31.26 | 0.00 | 31.28 | 0.00 | 27.43 | 0.00 | 30.04 | 0.00 |
| 513 | hsa-miR-152-5p | AGGTTCTGTGATACACTCCGACT | Yes | 30.76 | 0.00 | 54.43 | 0.00 | 57.80 | 0.00 | 42.19 | 0.00 |
| 514 | bta-miR-2468 | ATAGGAACATGGAAGATTGTCA | Yes | 30.76 | 0.00 | 21.12 | 0.00 | 25.47 | 0.00 | 17.90 | 0.00 |
| 515 | bta-miR-181c_R-1 | AACATTCAACCTGTCGGTGAGTT | Diff | 29.77 | 0.00 | 24.37 | 0.00 | 39.18 | 0.00 | 30.68 | 0.00 |
| 516 | bta-mir-2285n-4-p5_1ss10AT | AAAAAGTTCTTTTGGTTTTTTC | New | 29.77 | 0.00 | 50.37 | 0.00 | 48.49 | 0.00 | 37.71 | 0.00 |
| 517 | bta-miR-2285a_L-1R+1 | AAAACTGAATGAAATTCTTGGT | Diff | 29.77 | 0.00 | 34.93 | 0.00 | 17.63 | 0.00 | 35.80 | 0.00 |
| 518 | bta-miR-2346 | ACTGATGTGAAGGTGGTTTGGC | Yes | 29.77 | 0.00 | 41.43 | 0.00 | 44.08 | 0.00 | 34.52 | 0.00 |
| 519 | PC-3p-24213_18 | AGATGCACTGAATCTATTTGAG | New | 29.77 | 0.00 | 5.69 | 0.00 | 11.76 | 0.00 | 8.95 | 0.00 |
| 520 | pma-miR-29a-3p_L+1R-1 | CTAGCACCATTTGAAATCAGTT | Diff | 29.27 | 0.00 | 23.56 | 0.00 | 25.96 | 0.00 | 18.54 | 0.00 |
| 521 | PC-3p-11905_49 | AAAAACTAGAATGAACTTTTTG | New | 29.27 | 0.00 | 14.22 | 0.00 | 13.22 | 0.00 | 16.62 | 0.00 |
| 522 | bta-miR-142-5p_R+1 | CATAAAGTAGAAAGCACTACT | Diff | 28.78 | 0.00 | 16.25 | 0.00 | 15.67 | 0.00 | 6.39 | 0.00 |
| 523 | bta-miR-7858 | ACGCAATTCTTCAAAATCTTAGC | Yes | 28.78 | 0.00 | 47.12 | 0.00 | 20.57 | 0.00 | 25.57 | 0.00 |
| 524 | eca-mir-1543-p5 | TTTGCACCTCTGAGAGTGGAGT | New | 28.78 | 0.00 | 22.75 | 0.00 | 24.49 | 0.00 | 30.68 | 0.00 |
| 525 | bbe-miR-9-5p_R+2 | TCTTTGGTTATCTAGCTGTATGAAA | Diff | 28.78 | 0.00 | 39.00 | 0.00 | 14.69 | 0.00 | 43.47 | 0.00 |
| 526 | dre-miR-30e-3p_R+1_1ss22CT | CTTTCAGTCGGATGTTTGCAGTA | Diff | 28.78 | 0.00 | 72.30 | 0.00 | 39.18 | 0.00 | 49.86 | 0.00 |
| 527 | ssa-miR-125a-5p_R+1 | TCCCTGAGACCCTAACTTGTGAC | Diff | 28.28 | 0.00 | 19.09 | 0.00 | 14.69 | 0.00 | 36.44 | 0.00 |
| 528 | bta-miR-628 | ATGCTGACATATTTACTAGAGG | Yes | 27.78 | 0.00 | 34.12 | 0.00 | 36.25 | 0.00 | 31.96 | 0.00 |
| 529 | chi-mir-2284b-p3_1ss19AT | AAAATCTGAACAAACTTTTTGG | New | 27.78 | 0.00 | 26.81 | 0.00 | 11.76 | 0.00 | 26.85 | 0.00 |
| 530 | PC-3p-13131_43 | AAGCTTTAATTTATTAATCTGAT | New | 27.78 | 0.00 | 28.03 | 0.00 | 32.82 | 0.00 | 20.45 | 0.00 |
| 531 | PC-5p-7733_92 | TCAGCTGATCTTGGGTCT | New | 27.78 | 0.00 | 16.25 | 0.00 | 39.18 | 0.00 | 16.62 | 0.00 |
| 532 | PC-5p-34531_11 | TTGGCCAAAATGTTCGTTCAGA | New | 27.78 | 0.00 | 19.50 | 0.00 | 15.68 | 0.00 | 31.96 | 0.00 |
| 533 | PC-3p-9708_66 | AAAAACCCAAACGAACTTTTTGT | New | 27.29 | 0.00 | 16.25 | 0.00 | 13.71 | 0.00 | 14.06 | 0.00 |
| 534 | bta-miR-122 | TGGAGTGTGACAATGGTGTTTG | Yes | 26.79 | 0.00 | 62.56 | 0.00 | 69.55 | 0.00 | 71.59 | 0.00 |
| 535 | bta-miR-326_R+1 | CCTCTGGGCCCTTCCTCCAGT | Diff | 26.79 | 0.00 | 73.12 | 0.00 | 93.06 | 0.00 | 61.36 | 0.00 |
| 536 | aca-miR-200b-3p_R+2 | TAATACTGCCTGGTAATGATGATT | Diff | 26.79 | 0.00 | 98.30 | 0.00 | 57.80 | 0.00 | 89.49 | 0.00 |
| 537 | PC-3p-8617_78 | AAAACCTGAATGAACTTCTTGGT | New | 26.79 | 0.00 | 19.50 | 0.00 | 14.69 | 0.00 | 19.18 | 0.00 |
| 538 | bta-miR-99a-5p | AACCCGTAGATCCGATCTTGT | Yes | 26.30 | 0.00 | 44.28 | 0.00 | 16.16 | 0.00 | 54.33 | 0.00 |
| 539 | mmu-miR-330-5p_R+1 | TCTCTGGGCCTGTGTCTTAGGCT | Diff | 26.30 | 0.00 | 7.72 | 0.00 | 8.82 | 0.00 | 12.78 | 0.00 |
| 540 | eca-miR-330_R+1 | TCTCTGGGCCTGTGTCTTAGGCT | Diff | 26.30 | 0.00 | 7.72 | 0.00 | 8.82 | 0.00 | 12.78 | 0.00 |
| 541 | PC-5p-19755_24 | CAAGTTTGTTCAAGTTTTTCT | New | 25.80 | 0.00 | 33.30 | 0.00 | 6.86 | 0.00 | 6.40 | 0.00 |
| 542 | bta-mir-7-1-p3 | CAACAAGTCCCAGTCTGCCGCA | New | 25.80 | 0.00 | 26.81 | 0.00 | 21.55 | 0.00 | 17.90 | 0.00 |
| 543 | bta-miR-132_R+1 | TAACAGTCTACAGCCATGGTCGT | Diff | 25.80 | 0.00 | 30.87 | 0.00 | 42.12 | 0.00 | 29.40 | 0.00 |
| 544 | ssc-miR-769-3p_R-1_1ss19TC | CTGGGATCTCTGGGGTCTCGGT | Diff | 25.80 | 0.00 | 19.50 | 0.00 | 15.67 | 0.00 | 10.23 | 0.00 |
| 545 | bta-mir-2285o-1-p5_1ss2AC | ACAGAGTTCATTTGGGTTTTT | New | 25.80 | 0.00 | 16.25 | 0.00 | 10.12 | 0.00 | 10.55 | 0.00 |
| 546 | bta-miR-6517_R+2 | TCAGGGTCCGTGAGCTCCTCGGCGT | Diff | 25.80 | 0.00 | 38.18 | 0.00 | 18.61 | 0.00 | 29.40 | 0.00 |
| 547 | PC-3p-22103_20 | CACCTAGTGCATGGTCTTGGGC | New | 25.80 | 0.00 | 20.31 | 0.00 | 14.69 | 0.00 | 19.18 | 0.00 |
| 548 | PC-3p-6882_110 | AAAGCCCGAATGAACTTTTTAGT | New | 25.80 | 0.00 | 20.72 | 0.00 | 11.76 | 0.00 | 18.54 | 0.00 |
| 549 | bta-mir-29e-p5 | CTGGTTTCACATGGTGGCTTAGA | New | 25.30 | 0.00 | 51.18 | 0.00 | 38.20 | 0.00 | 45.38 | 0.00 |
| 550 | mdo-miR-29b-2-5p_L-1 | CTGGTTTCACATGGTGGCTTAGA | Diff | 25.30 | 0.00 | 51.18 | 0.00 | 38.20 | 0.00 | 45.38 | 0.00 |
| 551 | bta-miR-335 | TCAAGAGCAATAACGAAAAATGT | Yes | 24.81 | 0.00 | 28.43 | 0.00 | 18.61 | 0.00 | 26.85 | 0.00 |
| 552 | ssc-miR-664-5p_1ss14GA | CAGGCTAGGAGAAATGATTGGAT | Diff | 24.81 | 0.00 | 34.93 | 0.00 | 31.35 | 0.00 | 44.74 | 0.00 |
| 553 | PC-5p-19648_24 | TCCTAGGGGATTGACATAGTCT | New | 24.81 | 0.00 | 17.87 | 0.00 | 15.67 | 0.00 | 21.73 | 0.00 |
| 554 | PC-5p-16793_30 | CGATACTTATAGAGATAGATAGT | New | 24.81 | 0.00 | 14.62 | 0.00 | 12.73 | 0.00 | 20.45 | 0.00 |
| 555 | PC-3p-15964_32 | TAGTTTTATGTGTAAGCTCGTT | New | 24.81 | 0.00 | 17.06 | 0.00 | 12.73 | 0.00 | 25.57 | 0.00 |
| 556 | PC-5p-7121_105 | AAAAAGTTAGTTTAGGTTTTTC | New | 24.31 | 0.00 | 53.21 | 0.00 | 46.04 | 0.00 | 21.73 | 0.00 |
| 557 | PC-3p-5931_139 | AAACTCGAGCAAACTTTTTGGC | New | 24.31 | 0.00 | 15.84 | 0.00 | 23.51 | 0.00 | 26.21 | 0.00 |
| 558 | hsa-miR-195-3p_R+1 | CCAATATTGGCTGTGCTGCTCCA | Diff | 23.81 | 0.00 | 17.06 | 0.00 | 20.57 | 0.00 | 14.06 | 0.00 |
| 559 | bta-miR-2399-5p_R-1 | CTTTCTGTAGCTGTTGGAACT | Diff | 23.81 | 0.00 | 30.06 | 0.00 | 24.49 | 0.00 | 17.90 | 0.00 |
| 560 | xtr-miR-93b_L+2R+2 | CAAAGTGCTGTTCGTGCAGGTAGAT | Diff | 23.81 | 0.00 | 66.62 | 0.00 | 28.41 | 0.00 | 79.26 | 0.00 |
| 561 | ola-miR-103_R+4 | AGCAGCATTGTACAGGGCTATCATT | Diff | 23.81 | 0.00 | 43.87 | 0.00 | 32.33 | 0.00 | 37.07 | 0.00 |
| 562 | hsa-miR-4286_R+3 | ACCCCACTCCTGGTACCATT | Diff | 23.81 | 0.00 | 80.43 | 0.00 | 133.23 | 0.00 | 79.26 | 0.00 |
| 563 | PC-3p-7104_105 | AAAACTCAAACAAACTTTTTGGT | New | 23.81 | 0.00 | 34.93 | 0.00 | 18.12 | 0.00 | 17.26 | 0.00 |
| 564 | PC-3p-9872_64 | AAACTCAAACAAACTTTTTGGTT | New | 23.81 | 0.00 | 13.81 | 0.00 | 16.65 | 0.00 | 17.90 | 0.00 |
| 565 | PC-3p-16519_31 | GAAAACCCGAAGGAACTTTTTGT | New | 23.32 | 0.00 | 19.50 | 0.00 | 14.20 | 0.00 | 8.31 | 0.00 |
| 566 | bta-miR-126-5p | CATTATTACTTTTGGTACGCG | Yes | 22.82 | 0.00 | 9.75 | 0.00 | 9.80 | 0.00 | 10.23 | 0.00 |
| 567 | bta-miR-2284p | TGAAAGTTTGTTCGGGATTTT | Yes | 22.82 | 0.00 | 13.81 | 0.00 | 16.16 | 0.00 | 5.11 | 0.00 |
| 568 | bta-miR-2285p | AAAAACTTGAGTGAACTTTTGG | Yes | 22.82 | 0.00 | 31.68 | 0.00 | 20.57 | 0.00 | 37.07 | 0.00 |
| 569 | bta-mir-2285b-1-p5_1ss1GA | AAAAAGTTCATTCCAGTTTTTCT | New | 22.82 | 0.00 | 14.62 | 0.00 | 17.63 | 0.00 | 11.51 | 0.00 |
| 570 | bta-miR-2447 | TCTGGGAACCGGTTTGGCTGCT | Yes | 22.82 | 0.00 | 29.25 | 0.00 | 22.53 | 0.00 | 14.06 | 0.00 |
| 571 | PC-5p-13237_42 | TGAGTCTCATCTGAATCAGA | New | 22.82 | 0.00 | 13.81 | 0.00 | 15.67 | 0.00 | 8.95 | 0.00 |
| 572 | PC-5p-3461_312 | TCTTTGTAGTATCCTGAGT | New | 22.82 | 0.00 | 61.74 | 0.00 | 147.92 | 0.00 | 80.54 | 0.00 |
| 573 | bta-miR-218_R-1 | TTGTGCTTGATCTAACCATGT | Diff | 22.82 | 0.00 | 45.50 | 0.00 | 74.44 | 0.00 | 44.74 | 0.00 |
| 574 | PC-3p-14392_37 | AAATCCCGAACAAACTTTTTGT | New | 21.84 | 0.00 | 8.94 | 0.00 | 3.92 | 0.00 | 7.68 | 0.00 |
| 575 | PC-5p-15324_34 | TATTATTCCATACTTAAGGCT | New | 21.84 | 0.00 | 17.88 | 0.00 | 18.62 | 0.00 | 15.34 | 0.00 |
| 576 | bta-miR-144_R-3 | TACAGTATAGATGATGTAC | Diff | 21.83 | 0.00 | 24.37 | 0.00 | 13.71 | 0.00 | 3.84 | 0.00 |
| 577 | bta-miR-2335_L-1 | GATAATGATGACTAACTGAAT | Diff | 21.83 | 0.00 | 14.62 | 0.00 | 15.67 | 0.00 | 14.06 | 0.00 |
| 578 | bta-miR-2440 | TGCAGTGATGAGACCCTGGA | Yes | 21.83 | 0.00 | 21.94 | 0.00 | 56.82 | 0.00 | 31.96 | 0.00 |
| 579 | PC-3p-18955_25 | AGCCGGCTCTTGGGCTGTCCGCC | New | 21.83 | 0.00 | 13.81 | 0.00 | 18.61 | 0.00 | 16.62 | 0.00 |
| 580 | PC-3p-16948_30 | AAAACCGGGATGAATTTTTTGT | New | 21.83 | 0.00 | 21.12 | 0.00 | 9.80 | 0.00 | 26.85 | 0.00 |
| 581 | PC-3p-6434_121 | AAAAACCTGAATGACCCTTTT | New | 21.33 | 0.00 | 13.40 | 0.00 | 13.22 | 0.00 | 15.34 | 0.00 |
| 582 | PC-5p-20051_23 | AAAAGTTCGTTCGATTTTTCCT | New | 20.84 | 0.00 | 13.00 | 0.00 | 7.84 | 0.00 | 8.31 | 0.00 |
| 583 | PC-3p-32471_12 | AACTGTTAGGAGGCTTGGCTGCT | New | 20.84 | 0.00 | 7.31 | 0.00 | 15.67 | 0.00 | 15.34 | 0.00 |
| 584 | bta-miR-215 | ATGACCTATGAATTGACAGACA | Yes | 20.34 | 0.00 | 11.78 | 0.00 | 14.69 | 0.00 | 19.18 | 0.00 |
| 585 | PC-3p-15032_35 | AAAACACTCGAACAAACATTTT | New | 20.34 | 0.00 | 10.97 | 0.00 | 10.29 | 0.00 | 6.39 | 0.00 |
| 586 | PC-3p-25261_17 | AAAACCCGAATGTACCTTTTGA | New | 20.34 | 0.00 | 15.84 | 0.00 | 9.31 | 0.00 | 18.54 | 0.00 |
| 587 | hsa-miR-194-3p | CCAGTGGGGCTGCTGTTATCTG | Yes | 19.85 | 0.00 | 12.19 | 0.00 | 3.92 | 0.00 | 6.39 | 0.00 |
| 588 | bta-miR-2397-3p | CAGAGCAGTGGCAAGAACGCAT | Yes | 19.85 | 0.00 | 17.87 | 0.00 | 15.67 | 0.00 | 10.23 | 0.00 |
| 589 | ola-miR-100_R+6 | AACCCGTAGATCCGAACTTGTGTAA | Diff | 19.85 | 0.00 | 23.97 | 0.00 | 8.82 | 0.00 | 13.42 | 0.00 |
| 590 | PC-3p-13886_39 | ACCCAAAGGAACATTTTGGCT | New | 19.85 | 0.00 | 22.34 | 0.00 | 21.06 | 0.00 | 5.11 | 0.00 |
| 591 | PC-3p-12043_48 | ACCGGGCGGAAACACCAA | New | 19.85 | 0.00 | 30.87 | 0.00 | 20.57 | 0.00 | 19.18 | 0.00 |
| 592 | PC-5p-22035_21 | TTCCGGGCCCCTTGAATCTCGA | New | 19.85 | 0.00 | 11.37 | 0.00 | 10.78 | 0.00 | 8.95 | 0.00 |
| 593 | bta-miR-2285aa_R-1 | AAAACTGGAACGAACTTTTGGG | Diff | 19.35 | 0.00 | 17.47 | 0.00 | 21.06 | 0.00 | 10.87 | 0.00 |
| 594 | bta-miR-34c_R+1 | AGGCAGTGTAGTTAGCTGATTGC | Diff | 18.85 | 0.00 | 43.06 | 0.00 | 41.14 | 0.00 | 43.47 | 0.00 |
| 595 | bta-miR-2314 | TGCCCATGATGACTGCTGACC | Yes | 18.85 | 0.00 | 21.12 | 0.00 | 22.53 | 0.00 | 35.80 | 0.00 |
| 596 | ssc-mir-7857-p5_1ss12TG | CATAGCCAGTTGGGGAAGAATGC | New | 18.85 | 0.00 | 10.56 | 0.00 | 6.86 | 0.00 | 24.29 | 0.00 |
| 597 | PC-3p-31381_13 | AAAAACCCGAATGCACTTCTTG | New | 18.85 | 0.00 | 3.25 | 0.00 | 6.86 | 0.00 | 1.28 | 0.00 |
| 598 | PC-3p-14933_36 | CACATGGAGTTGCTGTTACAAT | New | 18.85 | 0.00 | 21.94 | 0.00 | 19.59 | 0.00 | 24.29 | 0.00 |
| 599 | bta-miR-502a_L-1R-2 | ATGCACCTGGGCAAGGATT | Diff | 18.52 | 0.00 | 22.74 | 0.00 | 33.96 | 0.00 | 23.86 | 0.00 |
| 600 | bta-miR-2411-5p | TGGAGTGACTGTCAGATGCAGCCA | Yes | 17.86 | 0.00 | 30.87 | 0.00 | 13.71 | 0.00 | 30.68 | 0.00 |
| 601 | bta-miR-4523 | GACCGAGAGGGCCTCGGCTGT | Yes | 17.86 | 0.00 | 10.56 | 0.00 | 10.78 | 0.00 | 10.23 | 0.00 |
| 602 | pma-let-7a_2ss12GA19AG | TGAGGTAGTAGATTGTATGGTT | Diff | 17.86 | 0.00 | 21.53 | 0.00 | 49.96 | 0.00 | 48.58 | 0.00 |
| 603 | PC-3p-21617_21 | TCCATCCCCTCTTGCCCACCAGT | New | 17.86 | 0.00 | 8.94 | 0.00 | 17.63 | 0.00 | 8.95 | 0.00 |
| 604 | bta-miR-6536_R+1 | TTTAAGTATACGATGAACTGCA | Diff | 17.86 | 0.00 | 6.50 | 0.00 | 17.64 | 0.00 | 8.94 | 0.00 |
| 605 | ssa-miR-9b-3p_R+1_2ss11GT19TA | TAAAGCTAGATAACCGAAAGTAC | Diff | 17.86 | 0.00 | 17.06 | 0.00 | 8.82 | 0.00 | 14.06 | 0.00 |
| 606 | PC-3p-14194_38 | CTAACCTGGATGAACTTTTTGG | New | 17.86 | 0.00 | 13.82 | 0.00 | 16.66 | 0.00 | 25.56 | 0.00 |
| 607 | bta-miR-2284n_1ss13CT | AAAAAGTTTATTTGGGTTTTT | Diff | 17.68 | 0.00 | 16.91 | 0.00 | 15.31 | 0.00 | 21.24 | 0.00 |
| 608 | bta-miR-2285m_R-1_1ss6GA | AAAACACAAATGAACTTTTTG | Diff | 17.06 | 0.00 | 9.58 | 0.00 | 6.62 | 0.00 | 3.46 | 0.00 |
| 609 | mmu-miR-101b-5p_L-1 | CGGTTATCATGGTACCGATGCT | Diff | 16.87 | 0.00 | 24.37 | 0.00 | 18.61 | 0.00 | 19.18 | 0.00 |
| 610 | mml-miR-145-3p_R-1 | GGATTCCTGGAAATACTGTTCT | Diff | 16.87 | 0.00 | 6.50 | 0.00 | 1.96 | 0.00 | 12.78 | 0.00 |
| 611 | hsa-miR-192-3p_R+1 | CTGCCAATTCCATAGGTCACAGT | Diff | 16.87 | 0.00 | 14.62 | 0.00 | 11.76 | 0.00 | 14.06 | 0.00 |
| 612 | bta-miR-211_R+1 | TTCCCTTTGTCATCCTTTGCCC | Diff | 16.87 | 0.00 | 39.81 | 0.00 | 51.43 | 0.00 | 33.24 | 0.00 |
| 613 | hsa-miR-877-3p_R+1 | TCCTCTTCTCCCTCCTCCCAGT | Diff | 16.87 | 0.00 | 21.12 | 0.00 | 17.63 | 0.00 | 7.67 | 0.00 |
| 614 | bta-miR-2331-3p_R+2 | ACCCTGCAGCCAAAGAAGCTACT | Diff | 16.87 | 0.00 | 21.12 | 0.00 | 17.63 | 0.00 | 12.78 | 0.00 |
| 615 | hsa-miR-3065-5p_R+2 | TCAACAAAATCACTGATGCTGGAGT | Diff | 16.87 | 0.00 | 28.43 | 0.00 | 21.55 | 0.00 | 25.57 | 0.00 |
| 616 | ssa-miR-155-5p_R+3 | TTAATGCTAATCGTGATAGGGGTTTA | Diff | 16.87 | 0.00 | 42.25 | 0.00 | 10.78 | 0.00 | 40.91 | 0.00 |
| 617 | cin-miR-7-5p_R+4 | TGGAAGACTAGTGATTTTGTTGTTAA | Diff | 16.87 | 0.00 | 30.87 | 0.00 | 7.84 | 0.00 | 21.73 | 0.00 |
| 618 | oan-miR-96-5p_R+2 | TTTGGCACTAGCACATTTTTGCTTT | Diff | 16.87 | 0.00 | 42.25 | 0.00 | 19.59 | 0.00 | 43.47 | 0.00 |
| 619 | PC-3p-18373_27 | TTAAAGTTGAAAAGTTAAGCAGT | New | 16.87 | 0.00 | 12.59 | 0.00 | 8.82 | 0.00 | 8.95 | 0.00 |
| 620 | PC-3p-14072_39 | TCGAACAAACTTTTTGGCCAACT | New | 16.37 | 0.00 | 13.81 | 0.00 | 14.20 | 0.00 | 15.34 | 0.00 |
| 621 | aca-miR-184-3p_R+2 | TGGACGGAGAACTGATAAGGGTTT | Diff | 15.88 | 0.00 | 34.94 | 0.00 | 9.80 | 0.00 | 23.02 | 0.00 |
| 622 | PC-5p-41258_9 | TAGGCCCTCAAGAGTTTAAC | New | 15.88 | 0.00 | 4.06 | 0.00 | 3.92 | 0.00 | 2.56 | 0.00 |
| 623 | bta-mir-186-p3 | TTTCTGATTTTATTTTAAGC | New | 15.88 | 0.00 | 8.94 | 0.00 | 11.76 | 0.00 | 7.67 | 0.00 |
| 624 | hsa-miR-196a-3p_L+1R+1 | TCGGCAACAAGAAACTGCCTGAGT | Diff | 15.88 | 0.00 | 24.37 | 0.00 | 26.45 | 0.00 | 16.62 | 0.00 |
| 625 | bta-mir-2419-p3_1ss15AG | AACAGGCGGGTGCTGATACGAT | New | 15.88 | 0.00 | 32.50 | 0.00 | 17.63 | 0.00 | 17.90 | 0.00 |
| 626 | bta-miR-2448-3p | GTGGTTGATTGGATCCGTGGGT | Yes | 15.88 | 0.00 | 17.06 | 0.00 | 22.53 | 0.00 | 12.78 | 0.00 |
| 627 | bta-miR-7863 | ATGGACTGTCACCTGAGGAGC | Yes | 15.88 | 0.00 | 6.50 | 0.00 | 7.84 | 0.00 | 2.56 | 0.00 |
| 628 | PC-5p-14462_37 | GATTCCCGGCCAATGCACCA | New | 15.88 | 0.00 | 12.19 | 0.00 | 11.76 | 0.00 | 14.06 | 0.00 |
| 629 | PC-3p-28322_14 | TAGTTCACCTATGTGTCTGTCC | New | 15.88 | 0.00 | 4.06 | 0.00 | 10.78 | 0.00 | 6.39 | 0.00 |
| 630 | bta-miR-346 | TGTCTGCCCGCATGCCTGCCTCT | Yes | 14.88 | 0.00 | 15.44 | 0.00 | 14.69 | 0.00 | 17.90 | 0.00 |
| 631 | bta-miR-592_L-1 | TTGTGTCAATATGCGATGATGT | Diff | 14.88 | 0.00 | 15.44 | 0.00 | 21.55 | 0.00 | 12.78 | 0.00 |
| 632 | bta-miR-2284ac | AAATGTTCGCTTGGCTTTTTCC | Yes | 14.88 | 0.00 | 19.50 | 0.00 | 13.71 | 0.00 | 14.06 | 0.00 |
| 633 | PC-3p-11943_49 | AAAGCCCAAATGAACTTTTTGTT | New | 14.88 | 0.00 | 22.75 | 0.00 | 7.84 | 0.00 | 10.23 | 0.00 |
| 634 | PC-3p-25832_16 | AGACGGTTTAATTTTTCAT | New | 14.88 | 0.00 | 5.69 | 0.00 | 6.86 | 0.00 | 11.51 | 0.00 |
| 635 | PC-3p-7720_92 | AAAACCCGAATGAACTTTTTGT | New | 14.88 | 0.00 | 11.78 | 0.00 | 8.33 | 0.00 | 14.06 | 0.00 |
| 636 | PC-3p-20118_23 | AGAAAGCCGGCGGCTGCAGCT | New | 14.88 | 0.00 | 13.00 | 0.00 | 29.39 | 0.00 | 33.24 | 0.00 |
| 637 | PC-3p-10186_61 | AAAAGCCCAAATGAACTTTTTGT | New | 14.88 | 0.00 | 22.75 | 0.00 | 9.80 | 0.00 | 12.15 | 0.00 |
| 638 | bta-miR-545-5p_L+2R-1 | CCTCAGTAAATGTTTATTGGAT | Diff | 14.88 | 0.00 | 17.88 | 0.00 | 10.78 | 0.00 | 14.06 | 0.00 |
| 639 | bta-mir-2285aa-p5 | CAAAAAGTTCGTCCAGATTTTTC | New | 14.88 | 0.00 | 10.56 | 0.00 | 7.84 | 0.00 | 6.40 | 0.00 |
| 640 | pma-miR-9a-5p_L-2R+3 | TTTGGTTATCTAGCTGTATGAGA | Diff | 14.88 | 0.00 | 19.50 | 0.00 | 14.70 | 0.00 | 19.18 | 0.00 |
| 641 | PC-3p-16961_30 | AAAATCCGAGTGAACTTTTTGGT | New | 14.88 | 0.00 | 7.32 | 0.00 | 1.96 | 0.00 | 8.94 | 0.00 |
| 642 | PC-3p-25971_16 | AAAACCTGAATGAGCTTTTCGGT | New | 14.88 | 0.00 | 8.94 | 0.00 | 5.88 | 0.00 | 14.06 | 0.00 |
| 643 | ssa-miR-30d-5p_2ss12TC19AG | TGTAAACATCCCTGACTGGAAGCT | Diff | 14.39 | 0.00 | 19.90 | 0.00 | 15.18 | 0.00 | 29.40 | 0.00 |
| 644 | PC-5p-13946_39 | AAAAGGATGAAGAGTTAAAGT | New | 13.90 | 0.00 | 8.12 | 0.00 | 21.56 | 0.00 | 19.18 | 0.00 |
| 645 | PC-3p-18806_26 | CTTTAACTCTTCATCCTTTTGG | New | 13.90 | 0.00 | 13.82 | 0.00 | 8.82 | 0.00 | 7.68 | 0.00 |
| 646 | bta-mir-2285p-p5 | AATTGTTCATTTGGGTTTTTCC | New | 13.89 | 0.00 | 7.31 | 0.00 | 17.63 | 0.00 | 14.06 | 0.00 |
| 647 | bta-miR-2355-3p | ATTGTCCTTGCTGTTTGGAGAT | Yes | 13.89 | 0.00 | 9.75 | 0.00 | 11.76 | 0.00 | 11.51 | 0.00 |
| 648 | bta-miR-2415-3p_R+1 | CCAGGCCTGCTGGACCGACGCC | Diff | 13.89 | 0.00 | 4.06 | 0.00 | 5.88 | 0.00 | 6.39 | 0.00 |
| 649 | bta-mir-2468-p3 | CCAATTTTCCATGTTCCTGTGC | New | 13.89 | 0.00 | 17.06 | 0.00 | 10.78 | 0.00 | 24.29 | 0.00 |
| 650 | bta-miR-2473_R+1 | AAATTGGCAGAGCTTCCTCTTT | Diff | 13.89 | 0.00 | 15.44 | 0.00 | 10.78 | 0.00 | 14.06 | 0.00 |
| 651 | bta-mir-2475-p5 | TCTTCAGGTTTGCCACTTCACA | New | 13.89 | 0.00 | 16.25 | 0.00 | 22.53 | 0.00 | 10.23 | 0.00 |
| 652 | bta-miR-2484_R-4 | GAGCTATGATGACTTTGATT | Diff | 13.89 | 0.00 | 24.37 | 0.00 | 35.27 | 0.00 | 37.07 | 0.00 |
| 653 | tch-let-7a-5p | TAAGGTAGTAGATTGTATAGTT | Yes | 13.89 | 0.00 | 20.31 | 0.00 | 35.27 | 0.00 | 16.62 | 0.00 |
| 654 | pma-miR-196b_R+3_1ss9AT | TAGGTAGTTTCATGTTGTTGGGTAT | Diff | 13.89 | 0.00 | 39.81 | 0.00 | 11.76 | 0.00 | 44.74 | 0.00 |
| 655 | cfa-miR-8903 | TCTTGGGCCCCACCCCCGGAGACT | Yes | 13.89 | 0.00 | 10.56 | 0.00 | 5.88 | 0.00 | 8.95 | 0.00 |
| 656 | PC-3p-19019_25 | AAAACCTGAATGAACCTTTTGGT | New | 13.89 | 0.00 | 6.50 | 0.00 | 2.94 | 0.00 | 2.56 | 0.00 |
| 657 | PC-3p-10077_62 | AAAAACCTGAATGACCCTTTTGG | New | 13.89 | 0.00 | 4.87 | 0.00 | 4.41 | 0.00 | 8.31 | 0.00 |
| 658 | PC-5p-15842_33 | TGACTAGGGACTGGCCCCGGGG | New | 13.40 | 0.00 | 20.31 | 0.00 | 19.59 | 0.00 | 28.76 | 0.00 |
| 659 | PC-3p-28155_15 | AAAACCCGAGTGAACTTTTTGG | New | 13.40 | 0.00 | 4.06 | 0.00 | 1.96 | 0.00 | 1.28 | 0.00 |
| 660 | PC-5p-19947_24 | AGAAAGTTTGTTCTGGTCTTT | New | 12.90 | 0.00 | 7.32 | 0.00 | 12.74 | 0.00 | 5.12 | 0.00 |
| 661 | PC-5p-23393_19 | AAAAAGTTCGTTCAAGTTTTCC | New | 12.90 | 0.00 | 4.88 | 0.00 | 5.88 | 0.00 | 3.84 | 0.00 |
| 662 | mmu-miR-19a-5p_L-1R-1 | AGTTTTGCATAGTTGCACTA | Diff | 12.90 | 0.00 | 14.62 | 0.00 | 17.63 | 0.00 | 8.95 | 0.00 |
| 663 | bta-miR-126-3p_L+1 | TCGTACCGTGAGTAATAATGCG | Diff | 12.90 | 0.00 | 21.94 | 0.00 | 17.63 | 0.00 | 8.95 | 0.00 |
| 664 | bta-miR-141 | TAACACTGTCTGGTAAAGATGG | Yes | 12.90 | 0.00 | 11.78 | 0.00 | 17.63 | 0.00 | 14.70 | 0.00 |
| 665 | bta-miR-145 | GTCCAGTTTTCCCAGGAATCCCT | Yes | 12.90 | 0.00 | 6.50 | 0.00 | 1.96 | 0.00 | 84.38 | 0.00 |
| 666 | hsa-miR-181b-3p_R+1_1ss16AG | CTCACTGAACAATGAGTGCAAT | Diff | 12.90 | 0.00 | 5.69 | 0.00 | 19.59 | 0.00 | 10.23 | 0.00 |
| 667 | bta-mir-502b-p5 | TAATTCTTGCTCCCCAGGTGAGAGT | New | 12.90 | 0.00 | 25.18 | 0.00 | 9.80 | 0.00 | 7.67 | 0.00 |
| 668 | bta-miR-2403_R+2 | CTCGGGAAGCTAGCTGGCCTTGT | Diff | 12.90 | 0.00 | 8.12 | 0.00 | 10.78 | 0.00 | 7.67 | 0.00 |
| 669 | bta-miR-2424_L+2 | ACAGATCTTTGGTAATCTGATGGCT | Diff | 12.90 | 0.00 | 14.62 | 0.00 | 14.69 | 0.00 | 23.01 | 0.00 |
| 670 | dre-miR-135a_R+1 | TATGGCTTTTTATTCCTATGTGAA | Diff | 12.90 | 0.00 | 25.18 | 0.00 | 6.86 | 0.00 | 19.18 | 0.00 |
| 671 | dre-miR-34a_L+3 | ATCTGGCAGTGTCTTAGCTGGTTGT | Diff | 12.90 | 0.00 | 15.44 | 0.00 | 10.78 | 0.00 | 6.39 | 0.00 |
| 672 | hsa-miR-320b_1ss22AT | AAAAGCTGGGTTGAGAGGGCAT | Diff | 12.90 | 0.00 | 8.94 | 0.00 | 9.80 | 0.00 | 10.23 | 0.00 |
| 673 | PC-3p-18810_26 | AGAACCTGAGTGGACTTTTGGT | New | 12.90 | 0.00 | 21.12 | 0.00 | 18.61 | 0.00 | 16.62 | 0.00 |
| 674 | PC-5p-36165_10 | ACTCCCGGTGGGCAACGCGCC | New | 12.90 | 0.00 | 9.75 | 0.00 | 2.94 | 0.00 | 8.95 | 0.00 |
| 675 | hsa-miR-339-5p_R+1_1ss22CA | TCCCTGTCCTCCAGGAGCTCAAGT | Diff | 12.73 | 0.00 | 22.34 | 0.00 | 14.86 | 0.00 | 10.65 | 0.00 |
| 676 | bta-miR-219 | AGAGTTGAGTCTGGACGTCCCG | Yes | 11.91 | 0.00 | 17.06 | 0.00 | 19.59 | 0.00 | 15.34 | 0.00 |
| 677 | hsa-miR-874-5p_1ss23AT | CGGCCCCACGCACCAGGGTAAGT | Diff | 11.91 | 0.00 | 4.87 | 0.00 | 4.90 | 0.00 | 19.18 | 0.00 |
| 678 | bta-miR-2323 | TGACCTGATCATGCTTACTGAGC | Yes | 11.91 | 0.00 | 4.06 | 0.00 | 6.86 | 0.00 | 12.78 | 0.00 |
| 679 | bta-miR-2367-5p_R+1 | ACCCTGTAACTCAGCCATCAGAGT | Diff | 11.91 | 0.00 | 18.69 | 0.00 | 10.78 | 0.00 | 23.01 | 0.00 |
| 680 | bta-miR-2382-3p_L-1R+1 | TACTTCCTGGTGCCTCGCCCCACT | Diff | 11.91 | 0.00 | 16.25 | 0.00 | 9.80 | 0.00 | 3.84 | 0.00 |
| 681 | bta-miR-2422_R+1 | TTGAGGGGACTGAGGTGCGGAGA | Diff | 11.91 | 0.00 | 13.00 | 0.00 | 8.82 | 0.00 | 5.11 | 0.00 |
| 682 | bta-miR-2475 | TGAAGTGTGTGAAACTGCAGCGG | Yes | 11.91 | 0.00 | 4.87 | 0.00 | 10.78 | 0.00 | 6.39 | 0.00 |
| 683 | bta-mir-6524-p5 | GTTAGGCCACTCAGAGTAACT | New | 11.91 | 0.00 | 8.12 | 0.00 | 6.86 | 0.00 | 8.95 | 0.00 |
| 684 | ptr-miR-449a_2ss20CG22TA | TGGCAGTGTATTGTTAGCTGGA | Diff | 11.91 | 0.00 | 9.75 | 0.00 | 9.80 | 0.00 | 7.67 | 0.00 |
| 685 | xtr-miR-15c_L+3R-1_1ss15CA | ATCTAGCAGCACATAATGGTTTGT | Diff | 11.91 | 0.00 | 16.25 | 0.00 | 11.76 | 0.00 | 11.51 | 0.00 |
| 686 | PC-5p-33637_11 | ATGCCCACACGCGTACGCCAGC | New | 11.91 | 0.00 | 18.69 | 0.00 | 21.55 | 0.00 | 8.95 | 0.00 |
| 687 | PC-3p-7112_105 | TTAACCTTCGGGCACTGG | New | 11.91 | 0.00 | 13.00 | 0.00 | 57.80 | 0.00 | 24.29 | 0.00 |
| 688 | PC-5p-10592_58 | GAAAGTTTGTTGGGGTTTTTT | New | 11.91 | 0.00 | 11.37 | 0.00 | 21.55 | 0.00 | 16.62 | 0.00 |
| 689 | PC-5p-37904_10 | TCTTGAACTGGCCCAGTGGTC | New | 11.91 | 0.00 | 8.94 | 0.00 | 12.73 | 0.00 | 2.56 | 0.00 |
| 690 | hsa-miR-320b_R-2_1ss20CT | AAAAGCTGGGTTGAGAGGGT | Diff | 11.90 | 0.00 | 11.38 | 0.00 | 22.54 | 0.00 | 5.12 | 0.00 |
| 691 | cgr-miR-532-5p_R+2_1 | CATGCCTTGAGTGTAGGACCGTAA | Diff | 11.90 | 0.00 | 6.50 | 0.00 | 5.88 | 0.00 | 12.78 | 0.00 |
| 692 | mdo-miR-106-5p_L-1R+1_1ss12TC | AAAGTGCTTACAGTGCAGGTAGA | Diff | 11.41 | 0.00 | 37.37 | 0.00 | 12.24 | 0.00 | 13.42 | 0.00 |
| 693 | PC-5p-19814_24 | CTCCCCTTAAGGACTTCATATT | New | 11.41 | 0.00 | 20.72 | 0.00 | 13.22 | 0.00 | 3.84 | 0.00 |
| 694 | PC-5p-13166_42 | AAAGGTTCGTTCGGGTTTTCC | New | 11.41 | 0.00 | 6.09 | 0.00 | 6.37 | 0.00 | 7.03 | 0.00 |
| 695 | PC-5p-50755_7 | TGTCCAATGAGATTTCAAACTG | New | 11.41 | 0.00 | 5.28 | 0.00 | 3.43 | 0.00 | 6.39 | 0.00 |
| 696 | PC-3p-15101_35 | AAAACCTGAATGAACTTATTGGT | New | 10.92 | 0.00 | 10.56 | 0.00 | 9.80 | 0.00 | 8.94 | 0.00 |
| 697 | PC-3p-21689_21 | AAAACCTGAAGAGACTTTTTGGT | New | 10.92 | 0.00 | 8.94 | 0.00 | 6.86 | 0.00 | 7.68 | 0.00 |
| 698 | PC-3p-61534_5 | AAAAACCCGAACAAACTTTGT | New | 10.92 | 0.00 | 2.44 | 0.00 | 6.86 | 0.00 | 2.56 | 0.00 |
| 699 | PC-3p-50818_7 | CCTGAATGAACTTTTTGGCTAAGT | New | 10.92 | 0.00 | 4.06 | 0.00 | 0.98 | 0.00 | 7.68 | 0.00 |
| 700 | bta-mir-449c-p3 | CAGTTGCTAGTTGCACTCCCCT | New | 10.92 | 0.00 | 8.12 | 0.00 | 7.84 | 0.00 | 7.67 | 0.00 |
| 701 | bta-mir-2443-p3 | TCAGCCGGCCCTTCCCTCCCCT | New | 10.92 | 0.00 | 4.06 | 0.00 | 5.88 | 0.00 | 5.11 | 0.00 |
| 702 | bta-miR-2457_R+1 | TTGCCAACTGCAGAGCCGCGCTT | Diff | 10.92 | 0.00 | 11.37 | 0.00 | 16.65 | 0.00 | 16.62 | 0.00 |
| 703 | cgr-miR-1260_R+2 | ATCCCACCGCTGCCACCATA | Diff | 10.92 | 0.00 | 23.97 | 0.00 | 36.25 | 0.00 | 22.37 | 0.00 |
| 704 | PC-3p-26311_16 | ATTGGAGCCAGTCTGCAGAAGC | New | 10.92 | 0.00 | 4.06 | 0.00 | 3.92 | 0.00 | 8.95 | 0.00 |
| 705 | PC-3p-40931_9 | GAAAAACTCAAATGAACTTCT | New | 10.92 | 0.00 | 3.25 | 0.00 | 5.88 | 0.00 | 5.11 | 0.00 |
| 706 | PC-5p-29476_14 | TTGGCTCTGTGAGGTCGGCTCT | New | 10.92 | 0.00 | 14.62 | 0.00 | 9.80 | 0.00 | 6.39 | 0.00 |
| 707 | PC-3p-29481_14 | TTGATATGTCCTGAGACGCGGA | New | 10.92 | 0.00 | 3.25 | 0.00 | 8.82 | 0.00 | 11.51 | 0.00 |
| 708 | PC-5p-31671_12 | AGAAAGTTAATTCGAGTTTTT | New | 10.92 | 0.00 | 9.75 | 0.00 | 14.69 | 0.00 | 7.67 | 0.00 |
| 709 | PC-3p-13910_39 | TTGATGTATGTATTCTTGCAGT | New | 10.92 | 0.00 | 8.94 | 0.00 | 13.71 | 0.00 | 11.51 | 0.00 |
| 710 | PC-3p-22560_20 | AAGATTTATATTTAGCCT | New | 10.92 | 0.00 | 4.06 | 0.00 | 3.92 | 0.00 | 3.84 | 0.00 |
| 711 | bta-mir-2284ac-p3 | AAAAACCTGAGTGAACTTTTC | New | 10.67 | 0.00 | 7.72 | 0.00 | 3.92 | 0.00 | 6.87 | 0.00 |
| 712 | bta-miR-18b_R-3 | TAAGGTGCATCTAGTGCAG | Diff | 10.42 | 0.00 | 6.50 | 0.00 | 9.80 | 0.00 | 3.20 | 0.00 |
| 713 | PC-3p-20887_22 | AAAAACCTGAACAAACTTTTTGT | New | 10.42 | 0.00 | 4.87 | 0.00 | 4.90 | 0.00 | 2.56 | 0.00 |
| 714 | bta-miR-181d_R+1 | AACATTCATTGTTGTCGGTGGGTT | Diff | 10.25 | 0.00 | 17.06 | 0.00 | 9.14 | 0.00 | 15.77 | 0.00 |
| 715 | bta-mir-328-p5 | TGTTTCGGAGCCTGGAGCG | New | 9.92 | 0.00 | 3.25 | 0.00 | 0.98 | 0.00 | 3.84 | 0.00 |
| 716 | hsa-miR-499a-3p_L+1R-1 | GAACATCACAGCAAGTCTGTGC | Diff | 9.92 | 0.00 | 7.31 | 0.00 | 13.71 | 0.00 | 17.90 | 0.00 |
| 717 | bta-miR-2313-5p_R+2 | AGTGCAGCTGAGGACCAAGGCAGG | Diff | 9.92 | 0.00 | 6.50 | 0.00 | 8.82 | 0.00 | 21.73 | 0.00 |
| 718 | bta-miR-2331-5p | GGCTTCCGTGCCTGCAGATGTCT | Yes | 9.92 | 0.00 | 4.87 | 0.00 | 9.80 | 0.00 | 5.11 | 0.00 |
| 719 | bta-miR-2397-5p_R-1 | TGCGTTCTTACCACTGGCCTGT | Diff | 9.92 | 0.00 | 4.06 | 0.00 | 14.69 | 0.00 | 5.11 | 0.00 |
| 720 | bta-miR-6516 | TTTGCAGTAACAGGTGTGAAC | Yes | 9.92 | 0.00 | 34.12 | 0.00 | 51.92 | 0.00 | 47.30 | 0.00 |
| 721 | bta-miR-6520_R+3 | TTGAGTATTGTCAGAGAGAGCGAT | Diff | 9.92 | 0.00 | 16.25 | 0.00 | 15.67 | 0.00 | 10.23 | 0.00 |
| 722 | hsa-miR-219b-5p_L-1 | GATGTCCAGCCACAATTCTCG | Diff | 9.92 | 0.00 | 17.06 | 0.00 | 13.71 | 0.00 | 14.06 | 0.00 |
| 723 | ssc-miR-7134-5p_1ss18AG | ATGTCCGCGGGTTCCCTGTCC | Diff | 9.92 | 0.00 | 3.25 | 0.00 | 7.84 | 0.00 | 0 | 0.00 |
| 724 | efu-miR-9226_L-4 | GTCCCTGTTCGGGCGCCG | Diff | 9.92 | 0.00 | 9.75 | 0.00 | 14.69 | 0.00 | 12.78 | 0.00 |
| 725 | ola-let-7g-p3 | CTATACAGTCTACTGTCTTTCTT | Yes | 9.92 | 0.00 | 21.12 | 0.00 | 6.86 | 0.00 | 16.62 | 0.00 |
| 726 | PC-3p-31147_13 | CAACATTCTCAACCCCCAGGCT | New | 9.92 | 0.00 | 3.66 | 0.00 | 3.92 | 0.00 | 2.56 | 0.00 |
| 727 | PC-3p-19286_25 | TATAGTCAGGTGAATAACAGGT | New | 9.92 | 0.00 | 10.97 | 0.00 | 8.33 | 0.00 | 6.39 | 0.00 |
| 728 | PC-5p-16518_31 | AAAAAGTTTGTTAAGGTTTTT | New | 9.92 | 0.00 | 11.37 | 0.00 | 12.73 | 0.00 | 9.59 | 0.00 |
| 729 | PC-3p-68803_5 | CCTCAGCCGCCCCCCCTCACAC | New | 9.92 | 0.00 | 8.12 | 0.00 | 3.92 | 0.00 | 7.67 | 0.00 |
| 730 | PC-3p-22567_20 | CTGCAGTTTCTTCCTTGACCA | New | 9.92 | 0.00 | 8.94 | 0.00 | 15.67 | 0.00 | 15.34 | 0.00 |
| 731 | PC-3p-23592_19 | TCGAACAAACTTTTTGGCCAAT | New | 9.92 | 0.00 | 10.56 | 0.00 | 8.82 | 0.00 | 6.40 | 0.00 |
| 732 | bta-mir-2285af-1-p3_1ss21GT | AAAAACTCAAACAAACTTTTTG | New | 9.26 | 0.00 | 7.31 | 0.00 | 8.49 | 0.00 | 8.10 | 0.00 |
| 733 | bta-mir-2284i-p3_1ss12TG | AAACCCGGAATGAACTTTTTGG | New | 9.18 | 0.00 | 16.65 | 0.00 | 1.96 | 0.00 | 2.56 | 0.00 |
| 734 | bta-mir-2284s-p3_1ss20GT | AAGACCTGAATGAACTTTTTGG | New | 9.06 | 0.00 | 11.48 | 0.00 | 6.18 | 0.00 | 10.72 | 0.00 |
| 735 | mdo-miR-219-5p_R+3 | TGATTGTCCAAACGCAATTCTCGT | Diff | 8.94 | 0.00 | 9.74 | 0.00 | 2.94 | 0.00 | 3.84 | 0.00 |
| 736 | chi-miR-345-3p_R+1_1ss22GA | CCCTGAACTAGGGGTCTGGAGAT | Diff | 8.94 | 0.00 | 7.32 | 0.00 | 5.88 | 0.00 | 12.78 | 0.00 |
| 737 | PC-3p-31419_13 | AAAACCTGAACGAACTTTTTGGTT | New | 8.94 | 0.00 | 7.32 | 0.00 | 6.86 | 0.00 | 5.12 | 0.00 |
| 738 | bta-miR-202 | TTCCTATGCATATACTTCTTT | Yes | 8.93 | 0.00 | 11.37 | 0.00 | 23.51 | 0.00 | 1.28 | 0.00 |
| 739 | bta-miR-677_R-5 | CTCACTGATGAGCAGCTT | Diff | 8.93 | 0.00 | 11.37 | 0.00 | 15.67 | 0.00 | 21.73 | 0.00 |
| 740 | bta-miR-2411-3p_R-3 | GCTGAACTGTCTTACTCCCACA | Diff | 8.93 | 0.00 | 14.62 | 0.00 | 15.67 | 0.00 | 20.45 | 0.00 |
| 741 | bta-miR-2459 | GCCAGATGATGGGAGCTGATT | Yes | 8.93 | 0.00 | 1.62 | 0.00 | 6.86 | 0.00 | 2.56 | 0.00 |
| 742 | bta-miR-2474 | TACCGGGCCAGCTGGAAGGAGA | Yes | 8.93 | 0.00 | 9.75 | 0.00 | 15.67 | 0.00 | 20.45 | 0.00 |
| 743 | mml-miR-7180-5p_1ss14AG | AGTGTACTTCCTGGGGCCTCT | Diff | 8.93 | 0.00 | 6.50 | 0.00 | 4.90 | 0.00 | 7.67 | 0.00 |
| 744 | ssc-mir-9851-p5 | CGCTGGCAGTGTTGGTGGCACT | New | 8.93 | 0.00 | 3.25 | 0.00 | 5.88 | 0.00 | 0 | 0.00 |
| 745 | efu-mir-16-2-p3_1ss13TC | GCGTGACAGGGACATAGCAACT | New | 8.93 | 0.00 | 6.50 | 0.00 | 11.76 | 0.00 | 5.11 | 0.00 |
| 746 | oan-miR-30d-5p_R+1_1ss12CA | TGTAAACATCCACGACTGGAAGCT | Diff | 8.93 | 0.00 | 84.08 | 0.00 | 16.65 | 0.00 | 26.85 | 0.00 |
| 747 | pma-miR-204-5p_R+2 | TTCCCTTTGTCATCCTATGCCTGT | Diff | 8.93 | 0.00 | 21.12 | 0.00 | 23.51 | 0.00 | 23.01 | 0.00 |
| 748 | mdo-miR-210-3p_L-2R+2 | TGTGCGTGTGACAGCGGCTGT | Diff | 8.93 | 0.00 | 12.19 | 0.00 | 15.67 | 0.00 | 14.06 | 0.00 |
| 749 | pma-miR-18a-5p_R+2 | TAAGGTGCATCTAGTGCAGATAGTT | Diff | 8.93 | 0.00 | 26.00 | 0.00 | 1.96 | 0.00 | 17.90 | 0.00 |
| 750 | dre-miR-155_R+4 | TTAATGCTAATCGTGATAGGGGTTAA | Diff | 8.93 | 0.00 | 13.81 | 0.00 | 2.94 | 0.00 | 6.39 | 0.00 |
| 751 | dre-miR-193a-3p_R+2 | AACTGGCCTACAAAGTCCCAGTTT | Diff | 8.93 | 0.00 | 40.62 | 0.00 | 6.86 | 0.00 | 11.51 | 0.00 |
| 752 | PC-5p-38864_10 | TAATTCTTTTGTTTTGAATACT | New | 8.93 | 0.00 | 6.50 | 0.00 | 11.76 | 0.00 | 5.11 | 0.00 |
| 753 | PC-3p-37419_10 | CCTGTTAAAGACTTTACCACT | New | 8.93 | 0.00 | 8.12 | 0.00 | 13.71 | 0.00 | 6.39 | 0.00 |
| 754 | PC-3p-17393_29 | ACACTGGTGCTCTGTGGGA | New | 8.93 | 0.00 | 4.06 | 0.00 | 13.71 | 0.00 | 1.28 | 0.00 |
| 755 | PC-5p-24989_17 | TGTGTGGTTTTTAGGATT | New | 8.93 | 0.00 | 0 | 0.00 | 4.90 | 0.00 | 3.84 | 0.00 |
| 756 | PC-5p-33536_12 | AACGGAAATTGTCCTCTCTCCT | New | 8.93 | 0.00 | 9.75 | 0.00 | 4.90 | 0.00 | 0 | 0.00 |
| 757 | PC-3p-28515_14 | AAAACCTGAATGAACTTATTGT | New | 8.93 | 0.00 | 8.94 | 0.00 | 1.96 | 0.00 | 3.84 | 0.00 |
| 758 | PC-3p-35139_11 | TGAGACGGTCTCCTTACCTCACT | New | 8.93 | 0.00 | 5.69 | 0.00 | 3.92 | 0.00 | 6.39 | 0.00 |
| 759 | bta-mir-2284b-p3_1ss10TA | AGAACCTGAATGAACTTTTTGG | New | 8.77 | 0.00 | 11.37 | 0.00 | 8.08 | 0.00 | 12.57 | 0.00 |
| 760 | bta-miR-2285y_L+1R-1_1ss13AG | AAAAACCCAAACGAACTTTTT | Diff | 8.43 | 0.00 | 4.47 | 0.00 | 6.04 | 0.00 | 5.11 | 0.00 |
| 761 | PC-3p-22551_20 | ACTGGTTTCAGGACAGATCTCT | New | 8.43 | 0.00 | 4.06 | 0.00 | 7.84 | 0.00 | 7.03 | 0.00 |
| 762 | PC-5p-38817_10 | TACCAGGTCCACTGATTTGAGA | New | 8.43 | 0.00 | 4.47 | 0.00 | 6.86 | 0.00 | 3.84 | 0.00 |
| 763 | PC-3p-25233_17 | AAAACCCAAATGAACTTATTGT | New | 8.43 | 0.00 | 3.25 | 0.00 | 4.90 | 0.00 | 2.56 | 0.00 |
| 764 | bta-mir-2285af-2-p5_1ss7CT | AAAAAGTTTGTTCAGGTTTTT | New | 8.30 | 0.00 | 1.49 | 0.00 | 3.43 | 0.00 | 4.26 | 0.00 |
| 765 | bta-mir-2285af-1-p5_1ss7CT | AAAAAGTTTGTTCAGGTTTTT | New | 8.30 | 0.00 | 1.49 | 0.00 | 3.43 | 0.00 | 4.26 | 0.00 |
| 766 | bta-mir-2285f-1-p5_1ss15AG | AAAAAGTTCATTTGGGTTTTCC | New | 8.27 | 0.00 | 12.59 | 0.00 | 14.37 | 0.00 | 7.24 | 0.00 |
| 767 | mmu-miR-196a-1-3p_R-1_2ss10CT19TC | CAACGACATTAAACCACCCGA | Diff | 7.94 | 0.00 | 6.50 | 0.00 | 14.69 | 0.00 | 11.51 | 0.00 |
| 768 | bta-mir-664a-p3 | TTCGTTTACTCCCTAGCCTCCA | New | 7.94 | 0.00 | 8.94 | 0.00 | 8.82 | 0.00 | 16.62 | 0.00 |
| 769 | bta-mir-2300a-p5 | TCAGCTAGTTTTGTCTCCCTGT | New | 7.94 | 0.00 | 4.87 | 0.00 | 4.90 | 0.00 | 2.56 | 0.00 |
| 770 | bta-miR-2308_R+1 | TTGGGCTTGCAGCAGAGAGTAAT | Diff | 7.94 | 0.00 | 7.31 | 0.00 | 4.90 | 0.00 | 10.23 | 0.00 |
| 771 | bta-mir-2357-p3_1ss21GT | CACACCTGTGCCAGCCTGAGT | New | 7.94 | 0.00 | 1.62 | 0.00 | 5.88 | 0.00 | 1.28 | 0.00 |
| 772 | bta-miR-2376 | CCTCTGAGATCTTGCTAGGCGCT | Yes | 7.94 | 0.00 | 1.62 | 0.00 | 10.78 | 0.00 | 8.95 | 0.00 |
| 773 | bta-miR-2377 | ACTGCACAGACCTAGGCTCT | Yes | 7.94 | 0.00 | 1.62 | 0.00 | 6.86 | 0.00 | 10.23 | 0.00 |
| 774 | bta-miR-2448-5p_R+1 | CTGCGGATACAACCAAACACGGT | Diff | 7.94 | 0.00 | 7.31 | 0.00 | 0 | 0.00 | 6.39 | 0.00 |
| 775 | bta-miR-2454-3p_L-1R+2 | CTCCTCTGGCCGCTCTCCTCT | Diff | 7.94 | 0.00 | 9.75 | 0.00 | 10.78 | 0.00 | 11.51 | 0.00 |
| 776 | bta-miR-2461-3p | TCAGACTGAGAGCAGTGTGCCT | Yes | 7.94 | 0.00 | 7.31 | 0.00 | 6.86 | 0.00 | 3.84 | 0.00 |
| 777 | bta-miR-6523a | TCTGGGGTAACTTTGAGCAGGG | Yes | 7.94 | 0.00 | 0 | 0.00 | 0.98 | 0.00 | 7.67 | 0.00 |
| 778 | rno-miR-196c-5p_R+1_1ss12GC | TAGGTAGTTTCCTGTTGTTGGGC | Diff | 7.94 | 0.00 | 10.16 | 0.00 | 9.31 | 0.00 | 13.42 | 0.00 |
| 779 | mdo-miR-196b_R+2 | TAGGTAGTTTCCTGTTGTTGGGC | Diff | 7.94 | 0.00 | 10.16 | 0.00 | 9.31 | 0.00 | 13.42 | 0.00 |
| 780 | ola-let-7e-p3 | TATACAATCTACTGTCTTTCCC | Yes | 7.94 | 0.00 | 8.12 | 0.00 | 6.86 | 0.00 | 15.34 | 0.00 |
| 781 | aca-miR-210-3p_R+1 | CTGTGCGTGTGACAGCGGCTAT | Diff | 7.94 | 0.00 | 9.75 | 0.00 | 5.88 | 0.00 | 10.23 | 0.00 |
| 782 | ssc-miR-450b-3p_L+1R-1_1ss21AT | ATTGGGAACATTTTGCATCCT | Diff | 7.94 | 0.00 | 7.31 | 0.00 | 8.16 | 0.00 | 1.28 | 0.00 |
| 783 | mmu-miR-450b-3p_R-1_2ss19GC21AT | ATTGGGAACATTTTGCATCCT | Diff | 7.94 | 0.00 | 7.31 | 0.00 | 8.16 | 0.00 | 1.28 | 0.00 |
| 784 | PC-5p-39161_9 | GGATTAATAAATCAAAGCTTTC | New | 7.94 | 0.00 | 4.87 | 0.00 | 6.86 | 0.00 | 1.28 | 0.00 |
| 785 | PC-5p-36071_10 | TAGCGGCGGCGGCCACCGAGA | New | 7.94 | 0.00 | 5.69 | 0.00 | 2.94 | 0.00 | 7.67 | 0.00 |
| 786 | PC-5p-23423_19 | TCCCCCTGCCTGGACACCTGGT | New | 7.94 | 0.00 | 5.69 | 0.00 | 15.67 | 0.00 | 7.67 | 0.00 |
| 787 | PC-5p-12163_47 | TCTTCCCGGCCAATGCACCA | New | 7.94 | 0.00 | 9.75 | 0.00 | 24.49 | 0.00 | 23.01 | 0.00 |
| 788 | PC-3p-18927_25 | AAATTCGAATGGACTTTTTGGC | New | 7.94 | 0.00 | 8.94 | 0.00 | 6.86 | 0.00 | 10.23 | 0.00 |
| 789 | PC-5p-20945_22 | ACAAAGTTTGTTCGGGTTTTA | New | 7.94 | 0.00 | 12.19 | 0.00 | 13.22 | 0.00 | 8.31 | 0.00 |
| 790 | PC-3p-19190_25 | AAAGGCCCAAATGAACTTTTTGT | New | 7.94 | 0.00 | 14.62 | 0.00 | 5.88 | 0.00 | 8.31 | 0.00 |
| 791 | PC-3p-27531_15 | GACCTGAATGAACTTTTTGGCT | New | 7.94 | 0.00 | 3.66 | 0.00 | 2.94 | 0.00 | 2.56 | 0.00 |
| 792 | bta-miR-1277 | TACGTAGATATATATGTATTTT | Yes | 7.44 | 0.00 | 15.44 | 0.00 | 7.35 | 0.00 | 8.31 | 0.00 |
| 793 | bta-mir-2285ae-p3 | AAACTTGAACGGACTTTTTGGC | New | 7.44 | 0.00 | 13.68 | 0.00 | 11.27 | 0.00 | 6.39 | 0.00 |
| 794 | hsa-miR-219b-3p | AGAATTGCGTTTGGACAATCAGT | Yes | 7.44 | 0.00 | 2.44 | 0.00 | 3.92 | 0.00 | 2.56 | 0.00 |
| 795 | hsa-miR-1277-3p | TACGTAGATATATATGTATTTT | Yes | 7.44 | 0.00 | 15.44 | 0.00 | 7.35 | 0.00 | 8.31 | 0.00 |
| 796 | PC-5p-14143_38 | AAAAGGTTCGTTCGGGTTTTC | New | 7.44 | 0.00 | 2.03 | 0.00 | 4.90 | 0.00 | 5.11 | 0.00 |
| 797 | bta-mir-2284l-p3_1ss8AG | AAAACTCGAGCAAACTTTT | New | 7.28 | 0.00 | 3.25 | 0.00 | 5.55 | 0.00 | 4.26 | 0.00 |
| 798 | mml-let-7a-2-3p | CTGTACAGCCTCCTAGCTTTCC | Yes | 6.95 | 0.00 | 4.06 | 0.00 | 3.92 | 0.00 | 8.95 | 0.00 |
| 799 | bta-mir-147-p5 | GTGGAAACACTTCTGCACAGGCT | New | 6.95 | 0.00 | 4.06 | 0.00 | 0.98 | 0.00 | 1.28 | 0.00 |
| 800 | bta-miR-1434-5p_R-2 | GTACATGATGACTAAAATTT | Diff | 6.95 | 0.00 | 9.75 | 0.00 | 10.78 | 0.00 | 16.62 | 0.00 |
| 801 | bta-mir-2285x-p5_1ss17GT | AAAAAGTTTGTTCAGGTTTTTTCT | New | 6.95 | 0.00 | 7.31 | 0.00 | 2.94 | 0.00 | 2.56 | 0.00 |
| 802 | bta-miR-2339_R+2 | TCACTGGACCTAGCAGAGCTCC | Diff | 6.95 | 0.00 | 3.25 | 0.00 | 4.90 | 0.00 | 2.56 | 0.00 |
| 803 | bta-miR-2388-5p_1ss23AT | AGCTCCCGTCTCCTCTGTGCTGT | Diff | 6.95 | 0.00 | 9.75 | 0.00 | 8.82 | 0.00 | 1.28 | 0.00 |
| 804 | bta-miR-2425-3p | TTCGTAGACCCTGCCCTCCT | Yes | 6.95 | 0.00 | 7.31 | 0.00 | 8.82 | 0.00 | 11.51 | 0.00 |
| 805 | bta-miR-2465_L+1R-1 | TTGAGCCACAGTAGAGCCTTGGA | Diff | 6.95 | 0.00 | 1.62 | 0.00 | 1.96 | 0.00 | 5.11 | 0.00 |
| 806 | bta-miR-3613a_1ss12TA | TGTTGTACTTTATTTTTTGTTC | Diff | 6.95 | 0.00 | 14.62 | 0.00 | 5.39 | 0.00 | 4.47 | 0.00 |
| 807 | bta-miR-6121-3p_L+1 | GCCGGATGATGGACACTGAGG | Diff | 6.95 | 0.00 | 22.75 | 0.00 | 24.49 | 0.00 | 19.18 | 0.00 |
| 808 | gga-miR-31-3p | TGCTATGCCAACATATTGTCATC | Yes | 6.95 | 0.00 | 4.06 | 0.00 | 10.78 | 0.00 | 6.39 | 0.00 |
| 809 | tni-mir-199-3-p3 | ACAGTAGTCTGCACATTGGTTAT | New | 6.95 | 0.00 | 4.87 | 0.00 | 4.90 | 0.00 | 6.39 | 0.00 |
| 810 | hsa-miR-486-5p_R+2 | TCCTGTACTGAGCTGCCCCGAGGT | Diff | 6.95 | 0.00 | 13.00 | 0.00 | 9.80 | 0.00 | 14.06 | 0.00 |
| 811 | hsa-miR-3613-5p_1ss12TA | TGTTGTACTTTATTTTTTGTTC | Diff | 6.95 | 0.00 | 14.62 | 0.00 | 5.39 | 0.00 | 4.47 | 0.00 |
| 812 | mmu-miR-6412_R-2_1ss15AT | TCGAAACCATCCTCTGCTAC | Diff | 6.95 | 0.00 | 7.31 | 0.00 | 2.94 | 0.00 | 3.84 | 0.00 |
| 813 | PC-5p-35473_11 | AAGAAATTTGTTCAGGTTTTT | New | 6.95 | 0.00 | 4.06 | 0.00 | 1.96 | 0.00 | 1.28 | 0.00 |
| 814 | PC-3p-49263_7 | AAAATAATGAATGAACTTTTTG | New | 6.95 | 0.00 | 8.12 | 0.00 | 4.90 | 0.00 | 1.28 | 0.00 |
| 815 | PC-5p-28970_14 | AACCCCCTTACTTCTGTTTAAGC | New | 6.95 | 0.00 | 8.94 | 0.00 | 7.84 | 0.00 | 6.39 | 0.00 |
| 816 | PC-3p-53940_6 | TTAAACAGAACTAAGGTGTTAGT | New | 6.95 | 0.00 | 3.25 | 0.00 | 1.96 | 0.00 | 2.56 | 0.00 |
| 817 | PC-5p-24558_18 | TTCTGCCTTCCTCTGGCCCTAGT | New | 6.95 | 0.00 | 8.12 | 0.00 | 8.82 | 0.00 | 12.78 | 0.00 |
| 818 | PC-3p-101350_3 | TCTACCCGTGATCGCTCAGCTT | New | 6.95 | 0.00 | 0 | 0.00 | 1.96 | 0.00 | 0 | 0.00 |
| 819 | PC-5p-21252_22 | AGTTGTAATAGTTTTGACT | New | 6.95 | 0.00 | 4.87 | 0.00 | 4.90 | 0.00 | 5.11 | 0.00 |
| 820 | PC-5p-51974_7 | CCTGTGAGGGCTTCACCCTGGA | New | 6.95 | 0.00 | 6.50 | 0.00 | 6.86 | 0.00 | 5.11 | 0.00 |
| 821 | PC-3p-35885_11 | AACGGGAGTGATCGTGTCATT | New | 6.95 | 0.00 | 3.25 | 0.00 | 7.84 | 0.00 | 1.28 | 0.00 |
| 822 | PC-5p-34920_11 | CACTGCTTGGACCCGACTCTGT | New | 6.95 | 0.00 | 4.06 | 0.00 | 7.84 | 0.00 | 5.11 | 0.00 |
| 823 | bta-mir-2285m-3-p5_1ss13TC_2 | AAAAGGTTCATTCGGGTTTTTC | New | 6.94 | 0.00 | 6.50 | 0.00 | 3.92 | 0.00 | 15.34 | 0.00 |
| 824 | PC-3p-45405_8 | GATAATTAACTGTCACAGCTGA | New | 6.94 | 0.00 | 4.06 | 0.00 | 1.96 | 0.00 | 1.28 | 0.00 |
| 825 | bta-mir-2285n-7-p5_1ss13CT | AAAAAGTTTGTTTGAGTTTTT | New | 6.65 | 0.00 | 1.64 | 0.00 | 3.33 | 0.00 | 3.74 | 0.00 |
| 826 | bta-miR-2285o | AAACCCGAACGAACTTTTGG | Yes | 6.52 | 0.00 | 3.45 | 0.00 | 5.40 | 0.00 | 4.26 | 0.00 |
| 827 | PC-3p-39223_9 | CGCAAATCAGTGGACCTGGTT | New | 6.45 | 0.00 | 6.50 | 0.00 | 3.43 | 0.00 | 3.20 | 0.00 |
| 828 | PC-3p-20865_22 | AAAACCAGAATGAACTTTTTTG | New | 6.45 | 0.00 | 2.84 | 0.00 | 7.35 | 0.00 | 10.23 | 0.00 |
| 829 | PC-3p-55134_6 | AACAACCTGGATGAACTTTTTGGT | New | 6.45 | 0.00 | 1.62 | 0.00 | 3.92 | 0.00 | 5.75 | 0.00 |
| 830 | PC-5p-25606_17 | AAAAAGTTCATTCAGGGTTTTG | New | 6.45 | 0.00 | 5.28 | 0.00 | 3.92 | 0.00 | 8.31 | 0.00 |
| 831 | PC-3p-28576_14 | CCCAAGTGAACTTGTTGGCCT | New | 6.45 | 0.00 | 0.81 | 0.00 | 2.94 | 0.00 | 1.28 | 0.00 |
| 832 | PC-3p-14724_36 | AAAACTTGAATGAACCTTTTGGT | New | 6.45 | 0.00 | 3.25 | 0.00 | 3.43 | 0.00 | 5.11 | 0.00 |
| 833 | eca-mir-8986a-p5 | GTCGAGGCTAGAGTCACGCTTGGGTA | New | 5.96 | 0.00 | 4.88 | 0.00 | 5.88 | 0.00 | 12.78 | 0.00 |
| 834 | mmu-miR-34b-3p_2ss9AG10CT | AATCACTAGTTCCACTGCCATC | Diff | 5.95 | 0.00 | 4.06 | 0.00 | 2.94 | 0.00 | 5.11 | 0.00 |
| 835 | hsa-miR-132-5p | ACCGTGGCTTTCGATTGTTACT | Yes | 5.95 | 0.00 | 17.06 | 0.00 | 12.73 | 0.00 | 6.39 | 0.00 |
| 836 | hsa-miR-181b-2-3p_R+1_1ss19CT | CTCACTGATCAATGAATGTAA | Diff | 5.95 | 0.00 | 6.50 | 0.00 | 1.96 | 0.00 | 6.39 | 0.00 |
| 837 | bta-miR-200c | TAATACTGCCGGGTAATGATGGA | Yes | 5.95 | 0.00 | 6.50 | 0.00 | 8.82 | 0.00 | 15.34 | 0.00 |
| 838 | bta-mir-660-p3_1ss22AT | CCTCCTATGTGCATGGATTACT | New | 5.95 | 0.00 | 4.06 | 0.00 | 2.94 | 0.00 | 5.11 | 0.00 |
| 839 | bta-miR-2284c_L-1R+2 | AAAAGTTCGTTTTGGTTTTTC | Diff | 5.95 | 0.00 | 12.19 | 0.00 | 15.67 | 0.00 | 6.39 | 0.00 |
| 840 | bta-miR-2289_L+1R+1 | CTCGTGTTCGAGCTCGCGTCCT | Diff | 5.95 | 0.00 | 2.44 | 0.00 | 5.88 | 0.00 | 3.84 | 0.00 |
| 841 | bta-miR-2450a_R+1 | TGGGCAGTAGAGGTGCATGTGT | Diff | 5.95 | 0.00 | 8.94 | 0.00 | 9.80 | 0.00 | 14.06 | 0.00 |
| 842 | bta-miR-6533_L+1 | ATGCGGCAGAGCATTAGAGAAC | Diff | 5.95 | 0.00 | 9.75 | 0.00 | 4.90 | 0.00 | 5.11 | 0.00 |
| 843 | cgr-miR-532-5p_R+2_2 | CATGCCTTGAGTGTAGGACCGTTA | Diff | 5.95 | 0.00 | 3.25 | 0.00 | 2.94 | 0.00 | 6.39 | 0.00 |
| 844 | aca-miR-125a-5p_R+1 | TCCCTGAGACCCTTAACCTGTGA | Diff | 5.95 | 0.00 | 0.81 | 0.00 | 2.94 | 0.00 | 2.56 | 0.00 |
| 845 | fru-miR-200a_R+3 | TAACACTGTCTGGTAACGATGTTTT | Diff | 5.95 | 0.00 | 47.12 | 0.00 | 14.69 | 0.00 | 35.80 | 0.00 |
| 846 | PC-3p-73984_4 | GTCCGGGGTTCCTTTGAAGA | New | 5.95 | 0.00 | 8.94 | 0.00 | 7.84 | 0.00 | 14.06 | 0.00 |
| 847 | PC-3p-43974_8 | TACGGGGAAGCTGGGGAAGGTC | New | 5.95 | 0.00 | 7.31 | 0.00 | 7.84 | 0.00 | 1.28 | 0.00 |
| 848 | PC-5p-25649_17 | TGGCCAAAATCTTTGTTTCAGT | New | 5.95 | 0.00 | 4.06 | 0.00 | 3.92 | 0.00 | 5.11 | 0.00 |
| 849 | PC-5p-41553_9 | ACTGGGGGTTGAGAATGTCGCT | New | 5.95 | 0.00 | 4.06 | 0.00 | 1.47 | 0.00 | 2.56 | 0.00 |
| 850 | PC-3p-26108_16 | CAAACCTGAGTCAACTTTATGG | New | 5.95 | 0.00 | 5.69 | 0.00 | 6.86 | 0.00 | 6.39 | 0.00 |
| 851 | PC-5p-49282_7 | TCTTCCCACCCAGTGCACCCAG | New | 5.95 | 0.00 | 3.25 | 0.00 | 0.98 | 0.00 | 1.28 | 0.00 |
| 852 | PC-3p-52873_6 | CACAGGCACACGTGTGTGAGCA | New | 5.95 | 0.00 | 4.06 | 0.00 | 2.94 | 0.00 | 2.56 | 0.00 |
| 853 | PC-3p-49326_7 | ACAACCTGAATGAACATTTTGG | New | 5.95 | 0.00 | 2.44 | 0.00 | 0 | 0.00 | 0 | 0.00 |
| 854 | PC-3p-56798_6 | CAGTCATCATCACGGCTGATGC | New | 5.95 | 0.00 | 4.06 | 0.00 | 4.90 | 0.00 | 2.56 | 0.00 |
| 855 | PC-5p-23159_19 | GCATGTGATGAAAGGCTGATT | New | 5.95 | 0.00 | 4.06 | 0.00 | 6.86 | 0.00 | 6.39 | 0.00 |
| 856 | PC-3p-54586_6 | TCCCCTTCCTTCCGGCCTCCGCCT | New | 5.95 | 0.00 | 3.25 | 0.00 | 2.94 | 0.00 | 2.56 | 0.00 |
| 857 | PC-3p-31123_13 | AAAAACCCCAGTGAACTTTTTGT | New | 5.95 | 0.00 | 2.44 | 0.00 | 1.96 | 0.00 | 2.56 | 0.00 |
| 858 | PC-3p-17706_28 | GGTGGAGAGGAAGGGACT | New | 5.95 | 0.00 | 9.75 | 0.00 | 3.92 | 0.00 | 7.67 | 0.00 |
| 859 | PC-5p-37885_10 | AAGTAACAGCATCTCCACTGGA | New | 5.95 | 0.00 | 5.69 | 0.00 | 8.82 | 0.00 | 5.11 | 0.00 |
| 860 | PC-3p-82934_4 | CGGGTAGGGCTGTGACCCTCG | New | 5.95 | 0.00 | 1.62 | 0.00 | 3.92 | 0.00 | 6.39 | 0.00 |
| 861 | hsa-miR-181c-3p_L-1R+1 | ACCATCGACCGTTGAGTGGACC | Diff | 5.46 | 0.00 | 5.69 | 0.00 | 4.90 | 0.00 | 5.11 | 0.00 |
| 862 | bta-miR-219-5p_R-1 | TGATTGTCCAAACGCAATTCT | Diff | 5.46 | 0.00 | 2.84 | 0.00 | 6.86 | 0.00 | 4.47 | 0.00 |
| 863 | bta-miR-2319b_L+1R-1_1ss21AT | CTACTCTGTATTAGGCACTTT | Diff | 5.46 | 0.00 | 0 | 0.00 | 1.96 | 0.00 | 1.28 | 0.00 |
| 864 | PC-5p-19314_25 | CTCCCCTTAAGGACTTCATA | New | 5.46 | 0.00 | 7.72 | 0.00 | 4.41 | 0.00 | 3.84 | 0.00 |
| 865 | bta-mir-29e-p3 | TTTTTCCATCTTTGTATC | New | 5.13 | 0.00 | 8.26 | 0.00 | 8.33 | 0.00 | 8.31 | 0.00 |
| 866 | bta-mir-2284k-p3_1ss12AT | AAAAAACCAAATGAACTTTTT | New | 5.13 | 0.00 | 3.66 | 0.00 | 4.93 | 0.00 | 6.39 | 0.00 |
| 867 | bta-miR-2285r_L-1_1ss10GA | GAAACCTGAATGAACTTTTTGG | Diff | 4.99 | 0.00 | 3.34 | 0.00 | 1.18 | 0.00 | 3.88 | 0.00 |
| 868 | bta-miR-127 | TCGGATCCGTCTGAGCTTGGCT | Yes | 4.96 | 0.00 | 26.81 | 0.00 | 3.92 | 0.00 | 2.56 | 0.00 |
| 869 | bta-miR-142-3p_L+3R-3 | TGTAGTGTTTCCTACTTTATGG | Diff | 4.96 | 0.00 | 3.25 | 0.00 | 6.86 | 0.00 | 1.28 | 0.00 |
| 870 | hsa-miR-144-5p_R+1 | GGATATCATCATATACTGTAAGT | Diff | 4.96 | 0.00 | 2.44 | 0.00 | 0.98 | 0.00 | 3.84 | 0.00 |
| 871 | bta-miR-149-3p | GAGGGAGGGACGGGGGCTGTGC | Yes | 4.96 | 0.00 | 8.94 | 0.00 | 7.84 | 0.00 | 2.56 | 0.00 |
| 872 | bta-miR-483_R+2 | TCACTCCTCTCCTCCCGTCTTCT | Diff | 4.96 | 0.00 | 7.31 | 0.00 | 9.80 | 0.00 | 5.11 | 0.00 |
| 873 | bta-mir-497-p3 | CGGCACTGTGGCCACGTC | New | 4.96 | 0.00 | 6.50 | 0.00 | 16.65 | 0.00 | 11.51 | 0.00 |
| 874 | bta-miR-615 | GGGGGTCCCCGGTGCTCGGATC | Yes | 4.96 | 0.00 | 5.69 | 0.00 | 13.71 | 0.00 | 7.67 | 0.00 |
| 875 | bta-mir-1842-p3 | TGAGCAGGCCTGTCAGGGCGTT | New | 4.96 | 0.00 | 0.81 | 0.00 | 7.84 | 0.00 | 3.84 | 0.00 |
| 876 | bta-mir-2285ad-p5 | AAAAGTTCGTTTCAGTTTTTC | New | 4.96 | 0.00 | 3.25 | 0.00 | 4.90 | 0.00 | 1.28 | 0.00 |
| 877 | bta-miR-2380 | GTGACTGTACTTTAAAAGGTTA | Yes | 4.96 | 0.00 | 0 | 0.00 | 0 | 0.00 | 0 | 0.00 |
| 878 | bta-miR-2382-5p | AGGGGAGTGCCTGGGAAGCTGT | Yes | 4.96 | 0.00 | 4.87 | 0.00 | 4.90 | 0.00 | 2.56 | 0.00 |
| 879 | bta-miR-2450c_R-1_1ss3CT | CATGTCAGTAGAGGCGCGTGT | Diff | 4.96 | 0.00 | 1.62 | 0.00 | 4.90 | 0.00 | 8.95 | 0.00 |
| 880 | bta-miR-6120-5p | CTGTTCCCGTTTTTCACATGTG | Yes | 4.96 | 0.00 | 4.87 | 0.00 | 5.88 | 0.00 | 7.67 | 0.00 |
| 881 | bta-miR-6525_R+3 | CTGGGGAAAGCAGGAGTGAGGGT | Diff | 4.96 | 0.00 | 8.94 | 0.00 | 5.88 | 0.00 | 2.56 | 0.00 |
| 882 | bta-miR-6535 | TAGGGGGAGCGGTTGGAGGACT | Yes | 4.96 | 0.00 | 3.25 | 0.00 | 4.90 | 0.00 | 8.95 | 0.00 |
| 883 | mdo-miR-22-5p_R+1_1ss15GA | AGTTCTTCAGTGGCAAGCTTTAC | Diff | 4.96 | 0.00 | 2.44 | 0.00 | 0.98 | 0.00 | 1.28 | 0.00 |
| 884 | ssa-miR-148a-3p | TCAGTGCATTACAGAACTTTGT | Yes | 4.96 | 0.00 | 2.84 | 0.00 | 2.45 | 0.00 | 1.92 | 0.00 |
| 885 | mmu-miR-101c_L+1R+2 | TACAGTACTGTGATAACTGATC | Diff | 4.96 | 0.00 | 19.50 | 0.00 | 10.78 | 0.00 | 8.95 | 0.00 |
| 886 | mmu-miR-5106_L-1R-4_1ss3GC | GCTCTGTAGCTCAGTTGG | Diff | 4.96 | 0.00 | 7.31 | 0.00 | 9.31 | 0.00 | 19.18 | 0.00 |
| 887 | mmu-miR-6238_L-1R-3_1ss12TC | TATTAGTCAGCGGAGGAA | Diff | 4.96 | 0.00 | 0 | 0.00 | 1.96 | 0.00 | 1.28 | 0.00 |
| 888 | PC-5p-41554_9 | AAAATGTTCGCTTGGCTTTTTC | New | 4.96 | 0.00 | 3.25 | 0.00 | 4.90 | 0.00 | 3.84 | 0.00 |
| 889 | PC-5p-67992_5 | AGACCATGGCCTCTGGTCCTC | New | 4.96 | 0.00 | 5.69 | 0.00 | 0 | 0.00 | 1.28 | 0.00 |
| 890 | PC-5p-19890_24 | AATGTTTGTTTGGATGTTTTGT | New | 4.96 | 0.00 | 11.37 | 0.00 | 6.86 | 0.00 | 7.67 | 0.00 |
| 891 | PC-5p-65104_5 | ACAGATCTGTCCTGAAACCAGC | New | 4.96 | 0.00 | 4.06 | 0.00 | 6.37 | 0.00 | 4.47 | 0.00 |
| 892 | PC-5p-22700_20 | ACGGTCAGGGTCAGTTGCT | New | 4.96 | 0.00 | 4.06 | 0.00 | 6.86 | 0.00 | 6.39 | 0.00 |
| 893 | PC-3p-50378_7 | AAACTGGGCTGAACTTTTTGGC | New | 4.96 | 0.00 | 2.44 | 0.00 | 3.92 | 0.00 | 6.39 | 0.00 |
| 894 | PC-3p-33386_12 | AGAACCTGAATGAACTTTTTGT | New | 4.96 | 0.00 | 2.44 | 0.00 | 4.90 | 0.00 | 12.78 | 0.00 |
| 895 | PC-3p-44120_8 | AAACCCAAGTGAACTTGTTGGCT | New | 4.96 | 0.00 | 0.81 | 0.00 | 0.98 | 0.00 | 1.28 | 0.00 |
| 896 | PC-5p-105116_3 | AGAAATGATAAGATTGAGTTA | New | 4.96 | 0.00 | 0.81 | 0.00 | 2.94 | 0.00 | 1.28 | 0.00 |
| 897 | PC-3p-86646_3 | TCATTGTGAAGCAGAATT | New | 4.96 | 0.00 | 0.81 | 0.00 | 0 | 0.00 | 1.28 | 0.00 |
| 898 | PC-5p-89283_3 | GTTGGTCAAGAAGTTCATTTGG | New | 4.96 | 0.00 | 2.44 | 0.00 | 3.92 | 0.00 | 5.11 | 0.00 |
| 899 | PC-3p-68793_5 | GAAGTTAAGGAAGAAACT | New | 4.96 | 0.00 | 0.81 | 0.00 | 0.98 | 0.00 | 2.56 | 0.00 |
| 900 | PC-3p-94323_3 | CGAGCCCGGAGTTCCCAGCCGC | New | 4.96 | 0.00 | 4.87 | 0.00 | 0.98 | 0.00 | 1.28 | 0.00 |
| 901 | PC-3p-63288_5 | TCAGCCTCTGTCTTCCCCAGT | New | 4.96 | 0.00 | 1.62 | 0.00 | 1.96 | 0.00 | 1.28 | 0.00 |
| 902 | PC-3p-66920_5 | AAAAACCCGAATGAACTTTTTT | New | 4.96 | 0.00 | 0 | 0.00 | 2.94 | 0.00 | 1.28 | 0.00 |
| 903 | PC-3p-85746_3 | ACATCCTGAATGAACTTTTTGG | New | 4.96 | 0.00 | 4.87 | 0.00 | 0.98 | 0.00 | 0 | 0.00 |
| 904 | PC-3p-58699_6 | AAACCTGAATGAACTTCTTGGCT | New | 4.96 | 0.00 | 2.03 | 0.00 | 2.45 | 0.00 | 4.47 | 0.00 |
| 905 | PC-3p-45931_8 | TAACAGTCGGGAGAGGAG | New | 4.96 | 0.00 | 4.06 | 0.00 | 3.92 | 0.00 | 5.11 | 0.00 |
| 906 | PC-3p-31996_12 | TCCTGTCACTGTTCCTCACATCT | New | 4.96 | 0.00 | 7.31 | 0.00 | 4.90 | 0.00 | 8.95 | 0.00 |
| 907 | PC-3p-33903_11 | AAAACCCTAATGAACTTTTTGT | New | 4.96 | 0.00 | 0.81 | 0.00 | 3.92 | 0.00 | 1.28 | 0.00 |
| 908 | PC-3p-73142_4 | AAAACCTGAGCAAACTTTTTGGT | New | 4.96 | 0.00 | 0.81 | 0.00 | 0.98 | 0.00 | 0 | 0.00 |
| 909 | PC-3p-24255_18 | CTGAAAGAACTTTCTGGCCAAT | New | 4.96 | 0.00 | 3.24 | 0.00 | 3.92 | 0.00 | 7.68 | 0.00 |
| 910 | PC-3p-26437_16 | AAAACCCGAAGGAACTTTTTGGT | New | 4.96 | 0.00 | 2.44 | 0.00 | 7.84 | 0.00 | 2.56 | 0.00 |
| 911 | PC-3p-58122_6 | GAATCTGAATGAACTTTGTGGC | New | 4.96 | 0.00 | 3.24 | 0.00 | 6.86 | 0.00 | 8.94 | 0.00 |
| 912 | PC-3p-40967_9 | AAACCTGAACAAGCTTTTTGGCAT | New | 4.96 | 0.00 | 13.00 | 0.00 | 6.86 | 0.00 | 7.68 | 0.00 |
| 913 | bta-miR-199c_L-1R+3 | ACAGTAGTCTGCACATTGGTTT | Diff | 4.47 | 0.00 | 2.44 | 0.00 | 5.63 | 0.00 | 3.20 | 0.00 |
| 914 | bta-miR-2284b_1ss9GT | AAAAGTTCTTTTGGTTTTTTC | Diff | 4.47 | 0.00 | 5.28 | 0.00 | 6.20 | 0.00 | 7.83 | 0.00 |
| 915 | PC-3p-84824_4 | AGGAAAACCAGAATGAACTTAT | New | 4.47 | 0.00 | 0 | 0.00 | 1.96 | 0.00 | 3.20 | 0.00 |
| 916 | PC-5p-20920_22 | CCAAAAGCTCATTCAGGTTTT | New | 4.47 | 0.00 | 2.03 | 0.00 | 2.94 | 0.00 | 1.28 | 0.00 |
| 917 | PC-5p-26150_16 | AAAAAGTTCGTTCGATTTTTCC | New | 4.47 | 0.00 | 4.47 | 0.00 | 0.98 | 0.00 | 1.28 | 0.00 |
| 918 | ggo-mir-128-p5 | TGTTCCTGAGCTGTTGGATT | New | 3.97 | 0.00 | 4.87 | 0.00 | 8.82 | 0.00 | 5.11 | 0.00 |
| 919 | bta-miR-135b | TATGGCTTTTCATTCCTATGTGA | Yes | 3.97 | 0.00 | 1.62 | 0.00 | 0.98 | 0.00 | 0 | 0.00 |
| 920 | bta-miR-1343-5p | TGGGGAGCGGCCCCCGGGCGGG | Yes | 3.97 | 0.00 | 4.87 | 0.00 | 4.90 | 0.00 | 7.67 | 0.00 |
| 921 | bta-miR-1434-3p | GAAGAAATCTAAGGTCTGAGG | Yes | 3.97 | 0.00 | 5.69 | 0.00 | 2.94 | 0.00 | 8.95 | 0.00 |
| 922 | bta-miR-2285m_L-1R+2_1ss6GC | AAACCCAAATGAACTTTTTGGCT | Diff | 3.97 | 0.00 | 0.81 | 0.00 | 1.96 | 0.00 | 2.56 | 0.00 |
| 923 | bta-miR-2330-5p | TGGGCTTGGGCAGTGAGGACTGG | Yes | 3.97 | 0.00 | 1.62 | 0.00 | 0.98 | 0.00 | 0 | 0.00 |
| 924 | bta-miR-2349_R-2 | TGGCACTTCTGGTCTCAGACT | Diff | 3.97 | 0.00 | 0.81 | 0.00 | 0 | 0.00 | 0 | 0.00 |
| 925 | bta-miR-2357_R-1 | TTGGTCTGGCACAGAGCATGTGA | Diff | 3.97 | 0.00 | 0.81 | 0.00 | 0 | 0.00 | 5.11 | 0.00 |
| 926 | bta-miR-2370-3p_R+2 | TAAGCAACTTTCCTTTCTCCACC | Diff | 3.97 | 0.00 | 16.25 | 0.00 | 10.78 | 0.00 | 3.84 | 0.00 |
| 927 | bta-mir-2381-p5 | CCTAGGACAGAGGAGCCCGATG | New | 3.97 | 0.00 | 2.44 | 0.00 | 3.92 | 0.00 | 5.11 | 0.00 |
| 928 | bta-miR-2381_1ss2AG | CGGGCTGCTCTGTGCTTGGCT | Diff | 3.97 | 0.00 | 1.62 | 0.00 | 7.84 | 0.00 | 5.11 | 0.00 |
| 929 | bta-miR-2396 | TCATGCACTGGGGTCTTGATGG | Yes | 3.97 | 0.00 | 6.50 | 0.00 | 3.92 | 0.00 | 3.84 | 0.00 |
| 930 | bta-miR-2398 | ATCTAGGGACGCGTCGGCAACT | Yes | 3.97 | 0.00 | 5.69 | 0.00 | 1.96 | 0.00 | 3.84 | 0.00 |
| 931 | bta-miR-2436-3p_L+2R-3 | TCCCCGCCCGACCGCCTCGCC | Diff | 3.97 | 0.00 | 0.81 | 0.00 | 12.73 | 0.00 | 6.39 | 0.00 |
| 932 | bta-mir-2447-p3 | CAGCCCCTCCTTTTCCCTGCT | New | 3.97 | 0.00 | 3.25 | 0.00 | 3.92 | 0.00 | 6.39 | 0.00 |
| 933 | bta-mir-2466-p3 | TGACGCAGCCCCTCCGTCTGACT | New | 3.97 | 0.00 | 0.81 | 0.00 | 0.98 | 0.00 | 0 | 0.00 |
| 934 | bta-miR-3064_L+1R-2 | TTTGCCACACTGCAACACCTTA | Diff | 3.97 | 0.00 | 7.31 | 0.00 | 3.92 | 0.00 | 3.84 | 0.00 |
| 935 | hsa-miR-4683_1ss23TC | TGGAGATCCAGTGCTCGCCCGAC | Diff | 3.97 | 0.00 | 0.81 | 0.00 | 4.90 | 0.00 | 0 | 0.00 |
| 936 | hsa-miR-7705_R+1_1ss18GC | AATAGCTCAGAATGTCACTTCTGT | Diff | 3.97 | 0.00 | 8.94 | 0.00 | 1.96 | 0.00 | 2.56 | 0.00 |
| 937 | ssa-miR-30d-3p_R+1_1ss18GA | CTTTCAGTCGGATGTTTACAGCA | Diff | 3.97 | 0.00 | 7.31 | 0.00 | 7.84 | 0.00 | 8.95 | 0.00 |
| 938 | pma-miR-31_R-1 | TGGCAAGATGCTGGCATAGC | Diff | 3.97 | 0.00 | 3.25 | 0.00 | 10.78 | 0.00 | 6.39 | 0.00 |
| 939 | dre-miR-34a_L+1 | CTGGCAGTGTCTTAGCTGGTTGT | Diff | 3.97 | 0.00 | 9.75 | 0.00 | 7.84 | 0.00 | 1.28 | 0.00 |
| 940 | dre-miR-152_R-1_2ss20TG21GA | TCAGTGCATGACAGAACTTGA | Diff | 3.97 | 0.00 | 2.84 | 0.00 | 2.45 | 0.00 | 1.92 | 0.00 |
| 941 | dre-miR-338_R+1 | TCCAGCATCAGTGATTTTGTTGT | Diff | 3.97 | 0.00 | 3.25 | 0.00 | 1.96 | 0.00 | 3.84 | 0.00 |
| 942 | mmu-let-7j_R-2 | TGAGGTATTAGTTTGTGCTGTT | Diff | 3.97 | 0.00 | 10.56 | 0.00 | 1.96 | 0.00 | 7.67 | 0.00 |
| 943 | cgr-miR-130a-3p_R+2 | CAGTGCAATGTTAAAAGGGCATCT | Diff | 3.97 | 0.00 | 11.37 | 0.00 | 4.90 | 0.00 | 6.39 | 0.00 |
| 944 | ccr-miR-132a_R+2 | TAACAGTCTACAGCCATGGTCGTT | Diff | 3.97 | 0.00 | 3.25 | 0.00 | 5.88 | 0.00 | 2.56 | 0.00 |
| 945 | hsa-miR-4448_1ss6CG | GGCTCGTTGGTCTAGGGGTA | Diff | 3.97 | 0.00 | 2.44 | 0.00 | 6.86 | 0.00 | 19.18 | 0.00 |
| 946 | mml-miR-6134_R+4_1ss18GT | TGAGGTAGTAGGATGTATAGTTA | Diff | 3.97 | 0.00 | 0 | 0.00 | 2.94 | 0.00 | 0 | 0.00 |
| 947 | PC-5p-55343_6 | AAAAAGTTCATTCGGGTTTCT | New | 3.97 | 0.00 | 1.22 | 0.00 | 0 | 0.00 | 3.84 | 0.00 |
| 948 | PC-5p-26036_16 | ATCCCAGCCGGGTCGAGGGACA | New | 3.97 | 0.00 | 8.12 | 0.00 | 8.82 | 0.00 | 7.67 | 0.00 |
| 949 | PC-3p-48027_7 | TCGGGAGACGGCTGACGGCCGT | New | 3.97 | 0.00 | 0.81 | 0.00 | 3.92 | 0.00 | 3.84 | 0.00 |
| 950 | PC-3p-52015_6 | AATTTTCAGTGCCTCCATCT | New | 3.97 | 0.00 | 6.50 | 0.00 | 3.92 | 0.00 | 0 | 0.00 |
| 951 | PC-3p-48235_7 | AAAACTCGAACGAACTTTTTT | New | 3.97 | 0.00 | 2.03 | 0.00 | 2.94 | 0.00 | 0 | 0.00 |
| 952 | PC-5p-27516_15 | TGGCCCCGAGGAAGCAAGGCTGT | New | 3.97 | 0.00 | 7.31 | 0.00 | 1.96 | 0.00 | 6.39 | 0.00 |
| 953 | PC-3p-112306_3 | CAGCTCTGCTCTTGGCTGGCC | New | 3.97 | 0.00 | 4.06 | 0.00 | 4.90 | 0.00 | 1.28 | 0.00 |
| 954 | PC-3p-74470_4 | TCTAGCTTCCTGTCTCAGCTGCT | New | 3.97 | 0.00 | 0 | 0.00 | 0.98 | 0.00 | 1.28 | 0.00 |
| 955 | PC-3p-85935_3 | AAAAACCCATGAACTTTTTGTT | New | 3.97 | 0.00 | 1.62 | 0.00 | 0.98 | 0.00 | 2.56 | 0.00 |
| 956 | PC-3p-181497_2 | AAAAACCCGAGTGAACTTTTGGT | New | 3.97 | 0.00 | 0.81 | 0.00 | 0 | 0.00 | 1.28 | 0.00 |
| 957 | PC-3p-47233_7 | TGTGTGGACGTACATACGCT | New | 3.97 | 0.00 | 0.81 | 0.00 | 3.92 | 0.00 | 5.11 | 0.00 |
| 958 | PC-5p-29340_14 | TCAAGAAAATTTTTGCACC | New | 3.97 | 0.00 | 4.87 | 0.00 | 2.94 | 0.00 | 1.28 | 0.00 |
| 959 | PC-5p-11284_53 | AAAAAGTTAGTTTAGGTTTTT | New | 3.97 | 0.00 | 9.34 | 0.00 | 6.37 | 0.00 | 5.75 | 0.00 |
| 960 | PC-3p-44324_8 | AAAACCTGAACGAACTTTTTGTT | New | 3.97 | 0.00 | 3.25 | 0.00 | 2.45 | 0.00 | 3.84 | 0.00 |
| 961 | PC-5p-55171_6 | ATAATTCAACTTATTCGT | New | 3.97 | 0.00 | 0 | 0.00 | 2.94 | 0.00 | 1.28 | 0.00 |
| 962 | PC-5p-40318_9 | AGTTCTACAGTCCACGATT | New | 3.97 | 0.00 | 0 | 0.00 | 4.90 | 0.00 | 5.11 | 0.00 |
| 963 | PC-5p-36429_10 | ATTATGTGCGTCAATTAT | New | 3.97 | 0.00 | 1.62 | 0.00 | 4.90 | 0.00 | 1.28 | 0.00 |
| 964 | PC-5p-23374_19 | CAGCCTCTGGCATGTTGGA | New | 3.97 | 0.00 | 6.50 | 0.00 | 24.49 | 0.00 | 3.84 | 0.00 |
| 965 | PC-5p-54741_6 | AGGAAGGGGCTTCTGAGCTTCT | New | 3.97 | 0.00 | 2.44 | 0.00 | 1.96 | 0.00 | 3.84 | 0.00 |
| 966 | PC-5p-47741_7 | TTGGTCAGAAAGTTCGTTTGGT | New | 3.97 | 0.00 | 4.06 | 0.00 | 0 | 0.00 | 1.28 | 0.00 |
| 967 | PC-3p-86133_3 | AAAACCCGAACTAACTTTTGAGT | New | 3.97 | 0.00 | 0 | 0.00 | 0.98 | 0.00 | 0 | 0.00 |
| 968 | PC-5p-132549_2 | CAGCGGTGGAGGTAGGAATCTCT | New | 3.97 | 0.00 | 6.50 | 0.00 | 0.98 | 0.00 | 0 | 0.00 |
| 969 | PC-5p-25160_17 | TAGGCCAAAAAGTTAACTTGGG | New | 3.97 | 0.00 | 10.56 | 0.00 | 4.90 | 0.00 | 3.84 | 0.00 |
| 970 | PC-3p-53531_6 | AAAACCTGAATGAATTTTTGGG | New | 3.97 | 0.00 | 1.62 | 0.00 | 0.98 | 0.00 | 1.28 | 0.00 |
| 971 | PC-5p-51171_7 | TAGAGAGAACCCCCAGACTTGG | New | 3.97 | 0.00 | 3.25 | 0.00 | 1.96 | 0.00 | 3.84 | 0.00 |
| 972 | bta-miR-1248_R-4 | ACCTTCTTGTATAAGCACTGTGC | Diff | 3.96 | 0.00 | 4.06 | 0.00 | 8.82 | 0.00 | 7.68 | 0.00 |
| 973 | PC-5p-23052_19 | GTCAAAAAGTTTATTTGGGTTT | New | 3.96 | 0.00 | 4.88 | 0.00 | 8.82 | 0.00 | 2.56 | 0.00 |
| 974 | PC-3p-26899_16 | AAAAACCTGAATGACCCTTTTGA | New | 3.96 | 0.00 | 4.88 | 0.00 | 3.92 | 0.00 | 6.40 | 0.00 |
| 975 | PC-3p-48980_7 | TCCTTTTCCCGCCTGTGCCGCCT | New | 3.96 | 0.00 | 3.24 | 0.00 | 4.90 | 0.00 | 6.40 | 0.00 |
| 976 | PC-3p-36672_10 | TTTCATCAAATTTTCAGGAAGA | New | 3.96 | 0.00 | 2.44 | 0.00 | 8.82 | 0.00 | 6.40 | 0.00 |
| 977 | bta-mir-2284z-1-p3_1ss20GT | AAAACCTGAACAAACTTTTTG | New | 3.92 | 0.00 | 4.57 | 0.00 | 3.26 | 0.00 | 3.41 | 0.00 |
| 978 | bta-mir-2285m-2-p5_1ss1CA | AAAAAGTTCATTTGGGTTTTC | New | 3.47 | 0.00 | 2.49 | 0.00 | 3.18 | 0.00 | 1.17 | 0.00 |
| 979 | bta-mir-2285m-3-p5_1ss13TC_1 | AAAAGGTTCATTCGGGTTTTT | New | 3.47 | 0.00 | 3.25 | 0.00 | 1.96 | 0.00 | 7.67 | 0.00 |
| 980 | rno-miR-196c-5p_R+1_1ss12GA | TAGGTAGTTTCATGTTGTTGGGG | Diff | 3.47 | 0.00 | 6.09 | 0.00 | 4.90 | 0.00 | 4.47 | 0.00 |
| 981 | oan-let-7f-5p_R+3 | TGAGGTAGTAGATTGTATAGTTATT | Diff | 3.47 | 0.00 | 30.47 | 0.00 | 5.39 | 0.00 | 31.32 | 0.00 |
| 982 | PC-3p-76500_4 | GAAAACCCGAAGGAACTTTTTT | New | 3.47 | 0.00 | 0 | 0.00 | 0 | 0.00 | 0 | 0.00 |
| 983 | PC-3p-37246_10 | AAAACCCGAAGGAACTTTTTTG | New | 3.47 | 0.00 | 2.84 | 0.00 | 1.96 | 0.00 | 0.64 | 0.00 |
| 984 | PC-5p-14701_36 | AAAAGGTTCGTTCGGGTTTTCC | New | 3.47 | 0.00 | 8.12 | 0.00 | 3.92 | 0.00 | 6.39 | 0.00 |
| 985 | bta-mir-2284y-4-p3_1ss11TC | CCCAAACAAACTTTTTGGCCC | New | 3.31 | 0.00 | 2.03 | 0.00 | 3.92 | 0.00 | 1.28 | 0.00 |
| 986 | bta-mir-2284p-p3_1ss22GT | AAAACCAGAACAAATTTTTTGT | New | 3.31 | 0.00 | 1.22 | 0.00 | 0 | 0.00 | 0 | 0.00 |
| 987 | hsa-miR-504-3p_L+1R+1_1ss7TC | AGGGAGCGCAGGGCAGGGTTTCT | Diff | 2.98 | 0.00 | 3.25 | 0.00 | 6.86 | 0.00 | 8.95 | 0.00 |
| 988 | bta-miR-2383 | CTGAGTGATGACTGCTGACC | Yes | 2.98 | 0.00 | 3.25 | 0.00 | 3.92 | 0.00 | 1.28 | 0.00 |
| 989 | cfa-miR-2387_L-1R-2 | CTGCAGACCCGGCCTTGGCAA | Diff | 2.98 | 0.00 | 0 | 0.00 | 0 | 0.00 | 5.11 | 0.00 |
| 990 | bta-miR-2408_R+1 | CACGTGTGTGAGCTCAGCCGGT | Diff | 2.98 | 0.00 | 3.25 | 0.00 | 5.88 | 0.00 | 3.84 | 0.00 |
| 991 | bta-miR-2416_R+1 | TGCAGTGCCCACTGTGGAAGATGC | Diff | 2.98 | 0.00 | 8.12 | 0.00 | 6.86 | 0.00 | 2.56 | 0.00 |
| 992 | bta-miR-6522 | TCGGAATTGTTTGTGTACCTGT | Yes | 2.98 | 0.00 | 8.12 | 0.00 | 6.86 | 0.00 | 8.95 | 0.00 |
| 993 | bta-miR-6534_R+1 | TCTAAGAGCAGTCAGGACGGATT | Diff | 2.98 | 0.00 | 4.06 | 0.00 | 2.94 | 0.00 | 3.84 | 0.00 |
| 994 | bta-mir-7865-p5 | AGGCTGTGTGCAGGCCTGCC | New | 2.98 | 0.00 | 8.12 | 0.00 | 11.76 | 0.00 | 5.11 | 0.00 |
| 995 | rno-miR-1843b-5p_L+1R-3_1ss19AG | CATGGAGGTCTCTGTCTGGCT | Diff | 2.98 | 0.00 | 7.31 | 0.00 | 3.92 | 0.00 | 2.56 | 0.00 |
| 996 | hsa-mir-1973-p3 | CCTGACCGTGCAAAGGTAGCA | New | 2.98 | 0.00 | 0.81 | 0.00 | 1.96 | 0.00 | 3.84 | 0.00 |
| 997 | mmu-miR-322-5p_R-1 | CAGCAGCAATTCATGTTTTGG | Diff | 2.98 | 0.00 | 4.06 | 0.00 | 2.94 | 0.00 | 2.56 | 0.00 |
| 998 | ssa-let-7i-2-3p | CTGCGCAAGCTACTGCCTTGCC | Yes | 2.98 | 0.00 | 3.25 | 0.00 | 2.94 | 0.00 | 1.28 | 0.00 |
| 999 | cin-miR-33_R+4 | GTGCATTGTAGTTGCATTGCAAT | Diff | 2.98 | 0.00 | 8.12 | 0.00 | 16.65 | 0.00 | 6.39 | 0.00 |
| 1,000 | mdo-miR-152-3p_R+3 | TCAGTGCATGACAGAACTTGGGTTT | Diff | 2.98 | 0.00 | 7.31 | 0.00 | 2.94 | 0.00 | 10.23 | 0.00 |
| 1,001 | pma-miR-204-5p_R+3 | TTCCCTTTGTCATCCTATGCCTGTT | Diff | 2.98 | 0.00 | 8.94 | 0.00 | 2.94 | 0.00 | 11.51 | 0.00 |
| 1,002 | efu-miR-503_L-2R+2 | GAGTATTGTTTCTGCTGCCCGGTT | Diff | 2.98 | 0.00 | 4.06 | 0.00 | 4.90 | 0.00 | 6.39 | 0.00 |
| 1,003 | PC-3p-155400_2 | GTACCAGCTGCCATGGTCCCG | New | 2.98 | 0.00 | 1.62 | 0.00 | 3.92 | 0.00 | 5.11 | 0.00 |
| 1,004 | PC-3p-62085_5 | GGTATCCGAATCAAAAGAACTT | New | 2.98 | 0.00 | 2.44 | 0.00 | 1.96 | 0.00 | 6.39 | 0.00 |
| 1,005 | PC-3p-52016_6 | AAACCAGAACGAACTTTTTGTT | New | 2.98 | 0.00 | 9.75 | 0.00 | 0.98 | 0.00 | 2.56 | 0.00 |
| 1,006 | PC-5p-57530_6 | CCGTGGGACAGAGTGGAAGGAGT | New | 2.98 | 0.00 | 4.06 | 0.00 | 2.94 | 0.00 | 6.39 | 0.00 |
| 1,007 | PC-5p-63636_5 | TCCTTCAGAAGTCACCCGGCT | New | 2.98 | 0.00 | 4.87 | 0.00 | 0.98 | 0.00 | 0 | 0.00 |
| 1,008 | PC-3p-43945_8 | TCTGACTTCACCTGGGATT | New | 2.98 | 0.00 | 3.25 | 0.00 | 3.92 | 0.00 | 3.84 | 0.00 |
| 1,009 | PC-5p-43657_8 | TATGTTATTCACCTGACTATAT | New | 2.98 | 0.00 | 3.25 | 0.00 | 9.31 | 0.00 | 1.28 | 0.00 |
| 1,010 | PC-3p-51349_7 | AAAACACGAACGAACTTTTCGG | New | 2.98 | 0.00 | 7.31 | 0.00 | 3.92 | 0.00 | 0 | 0.00 |
| 1,011 | PC-5p-45241_8 | ATGAAGTTTGTTTCAGGTTTTC | New | 2.98 | 0.00 | 4.06 | 0.00 | 6.86 | 0.00 | 0 | 0.00 |
| 1,012 | PC-5p-79822_4 | AAAAGTTCTCTTGGGTTTGCC | New | 2.98 | 0.00 | 0 | 0.00 | 3.92 | 0.00 | 0 | 0.00 |
| 1,013 | PC-5p-58920_6 | GCGCGCGCCGCGGCTGGAC | New | 2.98 | 0.00 | 8.94 | 0.00 | 5.88 | 0.00 | 7.67 | 0.00 |
| 1,014 | PC-5p-21831_21 | ACCTTTGTTCGGGTTTTTCTGT | New | 2.98 | 0.00 | 11.37 | 0.00 | 4.90 | 0.00 | 7.67 | 0.00 |
| 1,015 | PC-5p-23331_19 | ACAAAGTTTGTTCGGGTTTT | New | 2.98 | 0.00 | 4.06 | 0.00 | 6.37 | 0.00 | 1.92 | 0.00 |
| 1,016 | PC-3p-58827_6 | ACCTGAGAACTTTTTGGCCAAC | New | 2.98 | 0.00 | 1.62 | 0.00 | 0.98 | 0.00 | 5.11 | 0.00 |
| 1,017 | PC-5p-40890_9 | TAGCACTTGTGTTTGTTTTTGT | New | 2.98 | 0.00 | 5.69 | 0.00 | 4.90 | 0.00 | 1.28 | 0.00 |
| 1,018 | PC-3p-85153_3 | CACTGGTCGGGAGAGGGGAGGGT | New | 2.98 | 0.00 | 3.25 | 0.00 | 5.88 | 0.00 | 5.11 | 0.00 |
| 1,019 | PC-3p-36858_10 | AGATGTGGAACTTGAATTT | New | 2.98 | 0.00 | 5.69 | 0.00 | 2.94 | 0.00 | 3.84 | 0.00 |
| 1,020 | PC-3p-19385_25 | TAAAGTTGAAAAGTTAAGCAGT | New | 2.98 | 0.00 | 2.84 | 0.00 | 3.92 | 0.00 | 2.56 | 0.00 |
| 1,021 | PC-5p-16864_30 | AACGTAGGTTTTATTTTTT | New | 2.98 | 0.00 | 12.19 | 0.00 | 11.76 | 0.00 | 1.28 | 0.00 |
| 1,022 | PC-3p-37437_10 | TCTCATCATCCTTCTTCCCCAGT | New | 2.98 | 0.00 | 3.25 | 0.00 | 2.94 | 0.00 | 2.56 | 0.00 |
| 1,023 | PC-3p-24611_18 | AAAAACCTAAATGAACCTTTTGT | New | 2.98 | 0.00 | 5.28 | 0.00 | 6.37 | 0.00 | 5.75 | 0.00 |
| 1,024 | PC-3p-33062_12 | TGACCCAATTGAACTTTTTGGT | New | 2.98 | 0.00 | 2.44 | 0.00 | 5.88 | 0.00 | 0 | 0.00 |
| 1,025 | PC-5p-62254_5 | ATAAGATTTAAAATAAGATGCT | New | 2.98 | 0.00 | 6.50 | 0.00 | 1.96 | 0.00 | 5.11 | 0.00 |
| 1,026 | PC-5p-56825_6 | TCCCGAGCCTCTCGAATCCCGA | New | 2.98 | 0.00 | 5.69 | 0.00 | 0.98 | 0.00 | 2.56 | 0.00 |
| 1,027 | PC-5p-58650_6 | TAACAGGGATGAGCTATTGTTTT | New | 2.98 | 0.00 | 3.25 | 0.00 | 1.96 | 0.00 | 3.84 | 0.00 |
| 1,028 | bta-mir-2285n-5-p5_1ss12TG | CAAAAAGTTCAGTTGAGTTTTT | New | 2.81 | 0.00 | 5.98 | 0.00 | 11.92 | 0.00 | 5.11 | 0.00 |
| 1,029 | bta-mir-2284aa-1-p3_1ss21CT | AAAAACCCAAATGAACTTTTT | New | 2.61 | 0.00 | 5.31 | 0.00 | 7.72 | 0.00 | 2.93 | 0.00 |
| 1,030 | ssa-miR-9b-5p_R+1 | TCTTTGGTTATCTAGCTGAATG | Diff | 2.48 | 0.00 | 1.62 | 0.00 | 4.90 | 0.00 | 0.64 | 0.00 |
| 1,031 | bta-miR-34b_R+1 | AGGCAGTGTAATTAGCTGATTGT | Diff | 1.98 | 0.00 | 4.06 | 0.00 | 1.96 | 0.00 | 2.56 | 0.00 |
| 1,032 | bta-miR-149-3p_R+1 | GAGGGAGGGACGGGGGCTGTGCT | Diff | 1.98 | 0.00 | 0 | 0.00 | 0 | 0.00 | 3.84 | 0.00 |
| 1,033 | hsa-miR-197-5p | CGGGTAGAGAGGGCAGTGGGAGG | Yes | 1.98 | 0.00 | 0 | 0.00 | 0 | 0.00 | 5.11 | 0.00 |
| 1,034 | bta-miR-212 | ACCTTGGCTCTAGACTGCTTACT | Yes | 1.98 | 0.00 | 2.44 | 0.00 | 5.88 | 0.00 | 7.67 | 0.00 |
| 1,035 | hsa-miR-212-3p | TAACAGTCTCCAGTCACGGCC | Yes | 1.98 | 0.00 | 4.87 | 0.00 | 8.82 | 0.00 | 2.56 | 0.00 |
| 1,036 | bta-miR-223_R+1 | TGTCAGTTTGTCAAATACCCCAA | Diff | 1.98 | 0.00 | 6.50 | 0.00 | 0.98 | 0.00 | 0 | 0.00 |
| 1,037 | bta-miR-2284k_R+2_1ss11GT | GAAAAGTTCGTTCGGGTTTTTCT | Diff | 1.98 | 0.00 | 8.73 | 0.00 | 6.12 | 0.00 | 3.20 | 0.00 |
| 1,038 | bta-mir-2284r-p3 | TTACGAACTTTTGGCCCAACC | New | 1.98 | 0.00 | 1.62 | 0.00 | 4.90 | 0.00 | 1.28 | 0.00 |
| 1,039 | bta-mir-2285m-3-p5_1ss1AC | CAAAGGTTCATTTGGGTTTTT | New | 1.98 | 0.00 | 1.62 | 0.00 | 3.92 | 0.00 | 1.28 | 0.00 |
| 1,040 | bta-miR-2296_R+1 | TGGACAACTGAGGTTCCTGCTT | Diff | 1.98 | 0.00 | 3.25 | 0.00 | 0 | 0.00 | 0 | 0.00 |
| 1,041 | bta-miR-2350_R+1 | CAGCTCCTGTTTCTCTCCTCAGT | Diff | 1.98 | 0.00 | 8.12 | 0.00 | 3.92 | 0.00 | 16.62 | 0.00 |
| 1,042 | bta-miR-2368-3p_L+3R-1 | CAGACTGTCAGACCACCTCTGC | Diff | 1.98 | 0.00 | 3.25 | 0.00 | 5.88 | 0.00 | 2.56 | 0.00 |
| 1,043 | bta-miR-2392_L-1R+1 | TGGATGGGGGTGAGGGGTGCAT | Diff | 1.98 | 0.00 | 0.81 | 0.00 | 4.90 | 0.00 | 1.28 | 0.00 |
| 1,044 | bta-mir-2426-p3_1ss23CT | CTGACACCGGTCCACTTCCTTTT | New | 1.98 | 0.00 | 1.62 | 0.00 | 0.98 | 0.00 | 3.84 | 0.00 |
| 1,045 | bta-miR-2446_R+1 | TAAAATATATTTGAAATCGGGCCT | Diff | 1.98 | 0.00 | 3.25 | 0.00 | 1.96 | 0.00 | 1.28 | 0.00 |
| 1,046 | bta-miR-2471-5p | GAGGATCTGTCTAATCTCAGAGG | Yes | 1.98 | 0.00 | 0.81 | 0.00 | 0 | 0.00 | 3.84 | 0.00 |
| 1,047 | rno-miR-1843b-3p_R+1 | TCTGATCGTTCCCCTCCATACA | Diff | 1.98 | 0.00 | 6.50 | 0.00 | 8.82 | 0.00 | 5.11 | 0.00 |
| 1,048 | mdo-miR-499-5p_R+3 | TTAAGACTTGCAGTGATGTTTAAT | Diff | 1.98 | 0.00 | 5.69 | 0.00 | 0.98 | 0.00 | 2.56 | 0.00 |
| 1,049 | xtr-miR-19b_R+1_1ss11CT | TGTGCAAATCTATGCAAAACTGAC | Diff | 1.98 | 0.00 | 13.81 | 0.00 | 4.90 | 0.00 | 7.67 | 0.00 |
| 1,050 | dre-miR-27b-5p_R+1 | AGAGCTTAGCTGATTGGTGAACAT | Diff | 1.98 | 0.00 | 4.06 | 0.00 | 3.92 | 0.00 | 1.28 | 0.00 |
| 1,051 | cgr-miR-322-5p_R+2 | CAGCAGCAATTCATGTTTTGGAT | Diff | 1.98 | 0.00 | 1.62 | 0.00 | 0 | 0.00 | 6.39 | 0.00 |
| 1,052 | chi-miR-1271-3p_R+4 | AGTGCCTGCTATGTGCCAGGCATT | Diff | 1.98 | 0.00 | 6.09 | 0.00 | 1.96 | 0.00 | 0 | 0.00 |
| 1,053 | hsa-miR-1271-3p_R+2 | AGTGCCTGCTATGTGCCAGGCATT | Diff | 1.98 | 0.00 | 6.09 | 0.00 | 1.96 | 0.00 | 0 | 0.00 |
| 1,054 | PC-5p-61350_5 | TTCTGTAGATAAGCCCCAGTTA | New | 1.98 | 0.00 | 0.81 | 0.00 | 2.94 | 0.00 | 5.11 | 0.00 |
| 1,055 | PC-3p-55697_6 | AAAAACCCCAGTGAACTTTTTG | New | 1.98 | 0.00 | 0.81 | 0.00 | 3.92 | 0.00 | 0 | 0.00 |
| 1,056 | PC-3p-87128_3 | TCCTGTCCGATCAGCTCTAGACT | New | 1.98 | 0.00 | 0.81 | 0.00 | 0.98 | 0.00 | 3.84 | 0.00 |
| 1,057 | PC-3p-67478_5 | TCACTGTCTCTGCTCTGCAGT | New | 1.98 | 0.00 | 4.06 | 0.00 | 2.94 | 0.00 | 2.56 | 0.00 |
| 1,058 | PC-3p-57214_6 | AAAAACCCAAATGAACGTTTTGT | New | 1.98 | 0.00 | 2.44 | 0.00 | 2.94 | 0.00 | 6.39 | 0.00 |
| 1,059 | PC-5p-40921_9 | AAAAAGTTCATTTGGGTTGTCT | New | 1.98 | 0.00 | 1.62 | 0.00 | 3.92 | 0.00 | 1.28 | 0.00 |
| 1,060 | PC-5p-79326_4 | CGCTGAGCCTGTCACCTG | New | 1.98 | 0.00 | 0.81 | 0.00 | 4.90 | 0.00 | 7.67 | 0.00 |
| 1,061 | PC-5p-40383_9 | AAAAAGTTCGTTTGGGTTTTCTT | New | 1.98 | 0.00 | 4.87 | 0.00 | 2.94 | 0.00 | 1.28 | 0.00 |
| 1,062 | PC-5p-34175_11 | CTGGGTTGGCCCAAAAGTTCGT | New | 1.98 | 0.00 | 5.69 | 0.00 | 4.41 | 0.00 | 2.56 | 0.00 |
| 1,063 | PC-3p-59118_6 | TAGAAAAACTCGAATGAACATT | New | 1.98 | 0.00 | 4.06 | 0.00 | 3.92 | 0.00 | 1.28 | 0.00 |
| 1,064 | PC-5p-124153_2 | TACCTTGGCTGTTCTAACCAT | New | 1.98 | 0.00 | 2.44 | 0.00 | 3.92 | 0.00 | 0 | 0.00 |
| 1,065 | PC-5p-42018_9 | AAAAGGTTCGTTTGGGTTTTCT | New | 1.98 | 0.00 | 3.25 | 0.00 | 2.94 | 0.00 | 0 | 0.00 |
| 1,066 | PC-5p-72672_4 | CTGGGTTGGCCAAAGAGATCTT | New | 1.98 | 0.00 | 6.50 | 0.00 | 4.90 | 0.00 | 3.84 | 0.00 |
| 1,067 | PC-3p-55879_6 | AATAACTCGAGTAAACTTTTGG | New | 1.98 | 0.00 | 4.06 | 0.00 | 0.98 | 0.00 | 3.84 | 0.00 |
| 1,068 | PC-3p-147277_2 | CTACTCTTTCAATTTCAT | New | 1.98 | 0.00 | 0 | 0.00 | 3.92 | 0.00 | 0 | 0.00 |
| 1,069 | PC-3p-34499_11 | AGACGGAATAATGGTTGCT | New | 1.98 | 0.00 | 2.44 | 0.00 | 4.90 | 0.00 | 1.28 | 0.00 |
| 1,070 | PC-3p-97593_3 | CGTGGGCAGCTAGACCACCT | New | 1.98 | 0.00 | 3.25 | 0.00 | 0.98 | 0.00 | 0 | 0.00 |
| 1,071 | PC-5p-77182_4 | TAAAACATGGAAGCACTTACT | New | 1.98 | 0.00 | 4.06 | 0.00 | 1.96 | 0.00 | 2.56 | 0.00 |
| 1,072 | PC-3p-30287_13 | TGAAGTTGGAAAAGGCCCT | New | 1.98 | 0.00 | 3.25 | 0.00 | 2.94 | 0.00 | 5.11 | 0.00 |
| 1,073 | PC-5p-38422_10 | TCTTTGATTCCACAGACGGGAT | New | 1.98 | 0.00 | 3.25 | 0.00 | 2.94 | 0.00 | 1.28 | 0.00 |
| 1,074 | PC-3p-45943_8 | AAAAACCTGAGTGAACTTTTCT | New | 1.98 | 0.00 | 2.44 | 0.00 | 5.88 | 0.00 | 6.40 | 0.00 |
| 1,075 | PC-3p-81578_4 | AACAACCTGAATGAACTTTTTGGT | New | 1.98 | 0.00 | 7.32 | 0.00 | 0 | 0.00 | 0 | 0.00 |
| 1,076 | hsa-miR-129-1-3p | AAGCCCTTACCCCAAAAAGTAT | Yes | 1.49 | 0.00 | 8.12 | 0.00 | 5.88 | 0.00 | 6.39 | 0.00 |
| 1,077 | bta-mir-2284o-p5_1ss17GT | GGAAAGTTCGTTCGGGTTTTT | New | 1.49 | 0.00 | 10.97 | 0.00 | 2.56 | 0.00 | 3.20 | 0.00 |
| 1,078 | mml-miR-28-3p_1ss11TC | CACTAGATTGCGAGCTCCTGGA | Diff | 1.49 | 0.00 | 4.06 | 0.00 | 4.41 | 0.00 | 2.56 | 0.00 |
| 1,079 | aca-miR-301a-3p_R+3 | CAGTGCAATAGTATTGTCAAAGCACT | Diff | 1.49 | 0.00 | 3.25 | 0.00 | 0.49 | 0.00 | 3.20 | 0.00 |
| 1,080 | xtr-miR-301_R+3_1ss4TC | CAGCGCAATAGTATTGTCAAAGCATT | Diff | 1.49 | 0.00 | 27.62 | 0.00 | 1.47 | 0.00 | 9.59 | 0.00 |
| 1,081 | PC-3p-75837_4 | TCGAGGGGACGCCGCCGTCCTT | New | 1.49 | 0.00 | 0.81 | 0.00 | 3.43 | 0.00 | 0.64 | 0.00 |
| 1,082 | PC-3p-383363_1 | CTCAAATCAGTGGACCTGGTAGT | New | 1.49 | 0.00 | 0.81 | 0.00 | 4.41 | 0.00 | 1.92 | 0.00 |
| 1,083 | bta-mir-2285w-p5_1ss20CT | AAAAGTTCATTCAGATTTTTCT | New | 1.32 | 0.00 | 3.25 | 0.00 | 0 | 0.00 | 0 | 0.00 |
| 1,084 | bta-mir-3596-p5_1ss1CT | TAGTAGGTTGTATAGTTA | New | 1.24 | 0.00 | 3.11 | 0.00 | 2.69 | 0.00 | 5.75 | 0.00 |
| 1,085 | bta-miR-2329-3p_L+1R-3 | ATCTGTGATGTGAGCTGATA | Diff | 1.00 | 0.00 | 4.06 | 0.00 | 2.94 | 0.00 | 6.40 | 0.00 |
| 1,086 | bta-miR-187 | TCGTGTCTTGTGTTGCAGCCGG | Yes | 0.99 | 0.00 | 0.81 | 0.00 | 2.94 | 0.00 | 5.11 | 0.00 |
| 1,087 | bta-miR-205_R+1 | TCCTTCATTCCACCGGAGTCTGT | Diff | 0.99 | 0.00 | 9.75 | 0.00 | 5.88 | 0.00 | 85.65 | 0.00 |
| 1,088 | bta-mir-2285v-p5_1ss16GA | AGAAAGTTCATTTGGATTTT | New | 0.99 | 0.00 | 3.39 | 0.00 | 2.53 | 0.00 | 3.84 | 0.00 |
| 1,089 | bta-mir-2285x-p5 | TTGGGTTGGCCAAAAAGTTTGT | New | 0.99 | 0.00 | 2.44 | 0.00 | 5.22 | 0.00 | 1.49 | 0.00 |
| 1,090 | bta-miR-2332_L-6 | AAGGTCTTGGAGACAAAG | Diff | 0.99 | 0.00 | 0.81 | 0.00 | 5.88 | 0.00 | 5.11 | 0.00 |
| 1,091 | bta-mir-2336-p5 | CATTTCAAAGCTAAGGTTATGG | New | 0.99 | 0.00 | 1.62 | 0.00 | 1.96 | 0.00 | 3.84 | 0.00 |
| 1,092 | bta-miR-2388-3p_L-1R+2 | CTCGAGGAAGCTGGAGATGACT | Diff | 0.99 | 0.00 | 3.25 | 0.00 | 0.98 | 0.00 | 0 | 0.00 |
| 1,093 | bta-miR-2394_L-1 | TCTCCCCTCAATCTCAGGACT | Diff | 0.99 | 0.00 | 1.62 | 0.00 | 0 | 0.00 | 3.84 | 0.00 |
| 1,094 | bta-mir-2422-p3_1ss23GT | TCCGCTTCCCACGTCTCCTCAGT | New | 0.99 | 0.00 | 2.44 | 0.00 | 3.92 | 0.00 | 3.84 | 0.00 |
| 1,095 | bta-mir-2428-p5 | CGGCGGAGGTGAGCCGAC | New | 0.99 | 0.00 | 0.81 | 0.00 | 1.96 | 0.00 | 3.84 | 0.00 |
| 1,096 | bta-miR-2449_R+2 | TGGGCAGGAGACAGCAGGGCCT | Diff | 0.99 | 0.00 | 1.62 | 0.00 | 2.94 | 0.00 | 3.84 | 0.00 |
| 1,097 | bta-miR-2454-5p_R+1 | CTGGGGGCTGCCAGGCAGGAGGCT | Diff | 0.99 | 0.00 | 4.87 | 0.00 | 3.92 | 0.00 | 1.28 | 0.00 |
| 1,098 | bta-miR-2903_R+4 | TTCCGCGCTCTACGCCAGCCCGT | Diff | 0.99 | 0.00 | 2.44 | 0.00 | 3.92 | 0.00 | 5.11 | 0.00 |
| 1,099 | bta-miR-4449_L-1R+2 | GTCCCGGGGCCGCTCGAGGCACC | Diff | 0.99 | 0.00 | 1.62 | 0.00 | 5.88 | 0.00 | 2.56 | 0.00 |
| 1,100 | bta-mir-6520-p3_1ss22CT | CGTCTCTCCGCCTGTACTCAGT | New | 0.99 | 0.00 | 0.81 | 0.00 | 4.90 | 0.00 | 3.84 | 0.00 |
| 1,101 | hsa-mir-9-1-p5 | AGTGGTGTGGAGTCTTCA | New | 0.99 | 0.00 | 4.87 | 0.00 | 1.96 | 0.00 | 6.39 | 0.00 |
| 1,102 | hsa-miR-3065-3p_R+1_1ss22AG | TCAGCACCAGGATATTGTTGGGGT | Diff | 0.99 | 0.00 | 2.44 | 0.00 | 3.92 | 0.00 | 1.28 | 0.00 |
| 1,103 | eca-mir-9027-p3_1ss9GA | AGGAGCTGACCAATGAGTTGT | New | 0.99 | 0.00 | 3.25 | 0.00 | 2.94 | 0.00 | 8.95 | 0.00 |
| 1,104 | dre-miR-99_R+1 | AACCCGTAGATCCGATCTTGTGA | Diff | 0.99 | 0.00 | 3.25 | 0.00 | 1.96 | 0.00 | 7.67 | 0.00 |
| 1,105 | dre-miR-181b-5p_R+4 | AACATTCATTGCTGTCGGTGGGTTTC | Diff | 0.99 | 0.00 | 2.44 | 0.00 | 0 | 0.00 | 3.84 | 0.00 |
| 1,106 | mdo-miR-22-3p_R+1 | AAGCTGCCAGTTGAAGAACTGCA | Diff | 0.99 | 0.00 | 6.50 | 0.00 | 6.86 | 0.00 | 6.39 | 0.00 |
| 1,107 | pma-miR-181a-5p_L+3 | ATCAACATTCAACGCTGTCGGTGAGT | Diff | 0.99 | 0.00 | 4.87 | 0.00 | 0 | 0.00 | 3.84 | 0.00 |
| 1,108 | mmu-miR-434-3p | TTTGAACCATCACTCGACTCCT | Yes | 0.99 | 0.00 | 4.06 | 0.00 | 0 | 0.00 | 0 | 0.00 |
| 1,109 | hsa-miR-4443_L+1 | CTTGGAGGCGTGGGTTTT | Diff | 0.99 | 0.00 | 0 | 0.00 | 0.98 | 0.00 | 6.39 | 0.00 |
| 1,110 | hsa-miR-4508_L+2R-1 | AAGCGGGGCTGGGCGCGC | Diff | 0.99 | 0.00 | 4.06 | 0.00 | 7.84 | 0.00 | 6.39 | 0.00 |
| 1,111 | hsa-miR-4792_R+1_1ss9GT | CGGTGAGCTCTCGCTGGCC | Diff | 0.99 | 0.00 | 4.87 | 0.00 | 1.96 | 0.00 | 3.84 | 0.00 |
| 1,112 | ssc-miR-7857-3p_R+4 | ATTGTTCTCCAACCTGGCTCTTTACT | Diff | 0.99 | 0.00 | 1.62 | 0.00 | 0.98 | 0.00 | 3.84 | 0.00 |
| 1,113 | ola-let-7g_R+5_1ss18TC | TGAGGTAGTAGTTTGTACAGTTTTT | Diff | 0.99 | 0.00 | 34.12 | 0.00 | 4.90 | 0.00 | 16.62 | 0.00 |
| 1,114 | PC-5p-68676_5 | AAAAGTCCATTCATATTTTTCT | New | 0.99 | 0.00 | 5.69 | 0.00 | 0.98 | 0.00 | 0 | 0.00 |
| 1,115 | PC-3p-46497_8 | GAATGTGTCATTCCTCTGTAGT | New | 0.99 | 0.00 | 2.44 | 0.00 | 4.90 | 0.00 | 0 | 0.00 |
| 1,116 | PC-3p-109911_3 | TGTCATATATAGGCATTTATGT | New | 0.99 | 0.00 | 0 | 0.00 | 0 | 0.00 | 3.84 | 0.00 |
| 1,117 | PC-5p-85923_3 | TGGTAAAGTTTTTAACATGTAG | New | 0.99 | 0.00 | 0 | 0.00 | 0 | 0.00 | 3.84 | 0.00 |
| 1,118 | PC-3p-160902_2 | TCCCGGAGCCCCTGGCGGGGAGA | New | 0.99 | 0.00 | 0.81 | 0.00 | 2.94 | 0.00 | 5.11 | 0.00 |
| 1,119 | PC-3p-85105_3 | ACTGGGGCTTATCTACAGAAGT | New | 0.99 | 0.00 | 0 | 0.00 | 0 | 0.00 | 3.84 | 0.00 |
| 1,120 | PC-5p-55401_6 | TTGGCCAAAAACTTTTTTCAAGG | New | 0.99 | 0.00 | 1.62 | 0.00 | 2.94 | 0.00 | 5.11 | 0.00 |
| 1,121 | PC-3p-84257_4 | TGGACAAATTTGAATGAACTTT | New | 0.99 | 0.00 | 3.25 | 0.00 | 1.96 | 0.00 | 3.84 | 0.00 |
| 1,122 | PC-5p-109816_3 | AGATATAGCACTTTTGATT | New | 0.99 | 0.00 | 0 | 0.00 | 3.92 | 0.00 | 0 | 0.00 |
| 1,123 | PC-3p-80458_4 | AACCCCAAATGAACTTTTTAAGC | New | 0.99 | 0.00 | 1.62 | 0.00 | 0.98 | 0.00 | 5.11 | 0.00 |
| 1,124 | PC-3p-63546_5 | AAAACCGGAATGAACTTTTTGTA | New | 0.99 | 0.00 | 4.87 | 0.00 | 1.96 | 0.00 | 6.39 | 0.00 |
| 1,125 | PC-3p-94349_3 | TGCGGCCTGGGGAAGTGCTGTCT | New | 0.99 | 0.00 | 3.25 | 0.00 | 2.94 | 0.00 | 0 | 0.00 |
| 1,126 | PC-3p-113555_2 | AGCCAGGGTCCTTGCAGCGG | New | 0.99 | 0.00 | 0.81 | 0.00 | 0 | 0.00 | 3.84 | 0.00 |
| 1,127 | PC-5p-41210_9 | TCAGTGGCAGAGTGCAGGGACCT | New | 0.99 | 0.00 | 10.56 | 0.00 | 4.90 | 0.00 | 7.67 | 0.00 |
| 1,128 | PC-3p-51148_7 | AAAACCTGAACAAACTTTTTGTT | New | 0.99 | 0.00 | 3.25 | 0.00 | 2.94 | 0.00 | 1.28 | 0.00 |
| 1,129 | PC-3p-56946_6 | CTCTCCAGTGCGCACCCCCGTC | New | 0.99 | 0.00 | 3.25 | 0.00 | 2.94 | 0.00 | 10.23 | 0.00 |
| 1,130 | PC-3p-58388_6 | TTGTCCAGGAGGGGCGGCACT | New | 0.99 | 0.00 | 3.25 | 0.00 | 3.92 | 0.00 | 5.11 | 0.00 |
| 1,131 | PC-3p-36267_10 | TCGATCCCGGGTTTCGGCACC | New | 0.99 | 0.00 | 3.25 | 0.00 | 4.90 | 0.00 | 6.39 | 0.00 |
| 1,132 | PC-3p-43159_8 | AGAAAGTGTTAAGTATTCT | New | 0.99 | 0.00 | 1.62 | 0.00 | 4.90 | 0.00 | 0 | 0.00 |
| 1,133 | PC-3p-88831_3 | AAGATCCGAGTGAACTTTTTGG | New | 0.99 | 0.00 | 0 | 0.00 | 0 | 0.00 | 3.84 | 0.00 |
| 1,134 | PC-5p-36170_10 | AATGATGATGAATATGCTGTC | New | 0.99 | 0.00 | 4.06 | 0.00 | 6.86 | 0.00 | 5.11 | 0.00 |
| 1,135 | PC-3p-147231_2 | ACTCTCTGTAACACACCCTCCCT | New | 0.99 | 0.00 | 0 | 0.00 | 0 | 0.00 | 5.11 | 0.00 |
| 1,136 | PC-5p-51692_7 | ACGTGTGTACGTAGGCACAGA | New | 0.99 | 0.00 | 1.62 | 0.00 | 5.88 | 0.00 | 2.56 | 0.00 |
| 1,137 | PC-5p-610407_1 | GAAAGTCATGTTTGTTTCAGAAC | New | 0.99 | 0.00 | 3.25 | 0.00 | 0.98 | 0.00 | 0 | 0.00 |
| 1,138 | PC-5p-210905_1 | TTGTAGGGTCAGCCTCCCAGCCT | New | 0.99 | 0.00 | 0.81 | 0.00 | 0 | 0.00 | 3.84 | 0.00 |
| 1,139 | PC-3p-28815_14 | TCTGCAGAAGCTGTCAGTGCCT | New | 0.99 | 0.00 | 6.50 | 0.00 | 2.94 | 0.00 | 5.11 | 0.00 |
| 1,140 | bta-miR-2435 | AAACTCGAATGAACTCTTTGGC | Yes | 0.50 | 0.00 | 1.62 | 0.00 | 0.98 | 0.00 | 5.11 | 0.00 |
| 1,141 | mmu-miR-219c-3p_L+1R-1 | TCGAGAATTGCGTTTGGACAAT | Diff | 0.50 | 0.00 | 0 | 0.00 | 1.96 | 0.00 | 3.84 | 0.00 |
| 1,142 | cin-miR-200-3p_2ss19GA22AG | TAATACTGCCTGGTAATGATGG | Diff | 0.50 | 0.00 | 0.81 | 0.00 | 2.45 | 0.00 | 3.20 | 0.00 |
| 1,143 | PC-3p-42382_9 | AAAACCTGAACAGACTTTTGGGT | New | 0.50 | 0.00 | 1.22 | 0.00 | 4.41 | 0.00 | 3.84 | 0.00 |
| 1,144 | PC-3p-73428_4 | TCCGAATGAACTTTTTGGTCACC | New | 0.50 | 0.00 | 1.22 | 0.00 | 4.90 | 0.00 | 0 | 0.00 |
| 1,145 | PC-3p-73464_4 | CTGAAAGAACTTTCTGGCCAATT | New | 0.50 | 0.00 | 3.25 | 0.00 | 0.98 | 0.00 | 0 | 0.00 |
| 1,146 | hsa-miR-122-3p_L+1R-3 | AAACGCCATTATCACACTAA | Diff | 0 | 0.00 | 4.87 | 0.00 | 1.96 | 0.00 | 3.84 | 0.00 |
| 1,147 | hsa-miR-143-5p | GGTGCAGTGCTGCATCTCTGGT | Yes | 0 | 0.00 | 0 | 0.00 | 0.98 | 0.00 | 5.11 | 0.00 |
| 1,148 | hsa-miR-301b-5p | GCTCTGACGAGGTTGCACTACT | Yes | 0 | 0.00 | 0.81 | 0.00 | 4.90 | 0.00 | 1.28 | 0.00 |
| 1,149 | bta-miR-708 | AAGGAGCTTACAATCTAGCTGGG | Yes | 0 | 0.00 | 4.06 | 0.00 | 4.90 | 0.00 | 3.84 | 0.00 |
| 1,150 | mmu-miR-1839-3p_L-1 | GACCTACTTATCTACCAACAGC | Diff | 0 | 0.00 | 5.69 | 0.00 | 6.86 | 0.00 | 0 | 0.00 |
| 1,151 | bta-miR-2290 | GCCTCTGGTGGTCGGTTTGT | Yes | 0 | 0.00 | 2.44 | 0.00 | 1.96 | 0.00 | 5.11 | 0.00 |
| 1,152 | bta-mir-2396-p5_1ss23GA | ATCGAGAGCCCAGTGACTGGGAA | New | 0 | 0.00 | 5.69 | 0.00 | 0 | 0.00 | 0 | 0.00 |
| 1,153 | bta-miR-2439-3p_1ss22AT | TATCAGAAGACAGGTAGGCAGT | Diff | 0 | 0.00 | 2.44 | 0.00 | 1.96 | 0.00 | 3.84 | 0.00 |
| 1,154 | oan-miR-98_1ss17AG | TGAGGTAGTAAGTTGTGTTGTT | Diff | 0 | 0.00 | 1.62 | 0.00 | 9.80 | 0.00 | 5.12 | 0.00 |
| 1,155 | pma-miR-140_R+1 | CAGTGGTTTTACCCTATGGTAGC | Diff | 0 | 0.00 | 0 | 0.00 | 0.98 | 0.00 | 5.11 | 0.00 |
| 1,156 | dre-miR-141-3p | TAACACTGTCTGGTAACGATGC | Yes | 0 | 0.00 | 0 | 0.00 | 3.92 | 0.00 | 1.28 | 0.00 |
| 1,157 | gga-let-7d_R+1 | AGAGGTAGTGGGTTGCATAGTT | Diff | 0 | 0.00 | 0 | 0.00 | 4.90 | 0.00 | 0 | 0.00 |
| 1,158 | oan-miR-30f_1ss19CA | TGTAAACATCCTCCCCTCAGCT | Diff | 0 | 0.00 | 9.75 | 0.00 | 0 | 0.00 | 0 | 0.00 |
| 1,159 | aca-miR-31-5p_R+2_1ss4CA | AGGAAAGATGTTGGCATAGCTGA | Diff | 0 | 0.00 | 3.25 | 0.00 | 0 | 0.00 | 1.28 | 0.00 |
| 1,160 | oha-miR-34a-5p_R+3 | TGGCAGTGTCTTAGCTGGTTGTTGAA | Diff | 0 | 0.00 | 2.44 | 0.00 | 0 | 0.00 | 3.84 | 0.00 |
| 1,161 | oan-miR-153-1-5p_R+1 | GTCATTTTTGTGATCTGCAGCTT | Diff | 0 | 0.00 | 0 | 0.00 | 0 | 0.00 | 3.20 | 0.00 |
| 1,162 | ssa-miR-199a-3p_R+2 | ACAGTAGTCTGCACATTGGTTTT | Diff | 0 | 0.00 | 5.69 | 0.00 | 0 | 0.00 | 1.28 | 0.00 |
| 1,163 | cin-miR-200-3p_R+2 | TAATACTGCCTGGTAATGGTGACT | Diff | 0 | 0.00 | 1.62 | 0.00 | 0.98 | 0.00 | 5.11 | 0.00 |
| 1,164 | hsa-miR-708-3p_R+1_1ss3AT | CATCTAGACTGTGAGCTTCTAGA | Diff | 0 | 0.00 | 4.06 | 0.00 | 1.96 | 0.00 | 3.84 | 0.00 |
| 1,165 | ola-miR-1388-5p_R+1_1ss4AC | AGGCCTGTCCAACCTGAGAATGT | Diff | 0 | 0.00 | 8.12 | 0.00 | 0.98 | 0.00 | 5.11 | 0.00 |
| 1,166 | mmu-miR-5100_R-1 | TCGAATCCCAGCGGTGCCTC | Diff | 0 | 0.00 | 0 | 0.00 | 4.90 | 0.00 | 1.28 | 0.00 |
| 1,167 | cin-let-7a-5p_R+1_1ss15AG | TGAGGTAGTAGGTTGTGCAGTT | Diff | 0 | 0.00 | 3.25 | 0.00 | 0.98 | 0.00 | 0 | 0.00 |
| 1,168 | PC-5p-127186_2 | AAAAGTTCTTTTGGTTTTTTCA | New | 0 | 0.00 | 6.50 | 0.00 | 0.98 | 0.00 | 2.56 | 0.00 |
| 1,169 | PC-5p-27994_15 | TAACATGGAGGTGGCCAGAGT | New | 0 | 0.00 | 6.50 | 0.00 | 15.67 | 0.00 | 5.11 | 0.00 |
| 1,170 | PC-5p-27132_15 | AAACAGTTCGTTTGGGTTTTCT | New | 0 | 0.00 | 14.62 | 0.00 | 0 | 0.00 | 0 | 0.00 |
| 1,171 | PC-5p-105326_3 | AATGGAAGAAACAATGCTATCTGT | New | 0 | 0.00 | 3.25 | 0.00 | 0.98 | 0.00 | 0 | 0.00 |
| 1,172 | PC-3p-50310_7 | AAACCCTGAACGAACTTTTTGGT | New | 0 | 0.00 | 8.12 | 0.00 | 0 | 0.00 | 0 | 0.00 |
| 1,173 | PC-5p-69755_4 | ATAAAAGTTCGTTCGGGTTTTT | New | 0 | 0.00 | 3.25 | 0.00 | 0 | 0.00 | 0 | 0.00 |
| 1,174 | PC-3p-449774_1 | AAACCTCAAACAAACTTTTTGGA | New | 0 | 0.00 | 3.66 | 0.00 | 0 | 0.00 | 0 | 0.00 |
| 1,175 | PC-5p-64693_5 | TCAAATGCGTCAGGACCCTTGG | New | 0 | 0.00 | 1.62 | 0.00 | 4.90 | 0.00 | 0 | 0.00 |
| 1,176 | PC-5p-59883_5 | CCCCGCGGGGCGCGCGCC | New | 0 | 0.00 | 0 | 0.00 | 0.98 | 0.00 | 5.11 | 0.00 |
| 1,177 | PC-3p-90647_3 | CCAGGTGGGGAGTTTGACT | New | 0 | 0.00 | 0.81 | 0.00 | 0 | 0.00 | 3.84 | 0.00 |
| 1,178 | PC-3p-39828_9 | TGGGGAAGGTTTCCTGGGC | New | 0 | 0.00 | 1.62 | 0.00 | 2.94 | 0.00 | 5.11 | 0.00 |
| 1,179 | PC-3p-476293_1 | AGAAGCCCAAATGAACTTTTTTGT | New | 0 | 0.00 | 0 | 0.00 | 0 | 0.00 | 3.84 | 0.00 |
| 1,180 | PC-5p-76219_4 | AAGTTCATTCGGGCTTTTCCA | New | 0 | 0.00 | 3.25 | 0.00 | 0 | 0.00 | 0 | 0.00 |
| 1,181 | PC-3p-50596_7 | AAACCCCGAATGAACTTTTTGG | New | 0 | 0.00 | 8.12 | 0.00 | 0 | 0.00 | 0 | 0.00 |
| 1,182 | PC-3p-151557_2 | CTCAGCGGTACCTGCCCACA | New | 0 | 0.00 | 0.81 | 0.00 | 0 | 0.00 | 3.84 | 0.00 |
| 1,183 | PC-5p-448166_1 | CAGACTCCACCAGCCTCGGCTGT | New | 0 | 0.00 | 0 | 0.00 | 0 | 0.00 | 3.84 | 0.00 |
| 1,184 | PC-5p-57641_6 | ATAAAGTTCATTCGGGTTTTTT | New | 0 | 0.00 | 2.84 | 0.00 | 4.41 | 0.00 | 7.03 | 0.00 |
| 1,185 | PC-5p-84568_4 | CAAAAAGTTTATTTGGGTTTTTT | New | 0 | 0.00 | 3.25 | 0.00 | 0 | 0.00 | 0 | 0.00 |
| 1,186 | PC-5p-75273_4 | TCTTTGGCATGTTTAAGGCTGA | New | 0 | 0.00 | 0 | 0.00 | 3.92 | 0.00 | 3.84 | 0.00 |
| 1,187 | PC-3p-99511_3 | TTCCCAGTGGCTGAGTTCCGC | New | 0 | 0.00 | 4.87 | 0.00 | 0 | 0.00 | 0 | 0.00 |
| 1,188 | PC-5p-362862_1 | CCAAGGAGAGTATATGGCTCTCAACC | New | 0 | 0.00 | 0 | 0.00 | 3.92 | 0.00 | 2.56 | 0.00 |
| 1,189 | PC-5p-101281_3 | TGGGCGTTTGGCACTGTTCATG | New | 0 | 0.00 | 3.25 | 0.00 | 1.96 | 0.00 | 1.28 | 0.00 |
| 1,190 | PC-3p-24121_18 | AGAGCCCAAATGAACTTTTTGT | New | 0 | 0.00 | 8.94 | 0.00 | 9.80 | 0.00 | 1.92 | 0.00 |
| 1,191 | PC-3p-92413_3 | TTTCCAGAGCTCTAGTTGTT | New | 0 | 0.00 | 0 | 0.00 | 0 | 0.00 | 3.84 | 0.00 |
| 1,192 | PC-5p-98541_3 | TTGGGCCTTCTCTCCCTAGT | New | 0 | 0.00 | 0.81 | 0.00 | 3.92 | 0.00 | 0 | 0.00 |
| 1,193 | PC-5p-169941_2 | AAAAAGTTTGTTTGGTTTTCT | New | 0 | 0.00 | 3.25 | 0.00 | 0 | 0.00 | 0 | 0.00 |
| 1,194 | PC-5p-61386_5 | AAGTTTGTTCAAGTTTTTCTGT | New | 0 | 0.00 | 3.25 | 0.00 | 0.98 | 0.00 | 0 | 0.00 |
| 1,195 | PC-5p-95471_3 | TTCCTAATAGACAAAGAGCTGA | New | 0 | 0.00 | 1.62 | 0.00 | 4.90 | 0.00 | 6.39 | 0.00 |
| 1,196 | PC-5p-53042_6 | TTCTCAGTGGCTCAGTTCTGC | New | 0 | 0.00 | 10.56 | 0.00 | 0.98 | 0.00 | 1.28 | 0.00 |
| 1,197 | PC-5p-97406_3 | CGGTGGTGTTTGCCGGCGCCCGGCC | New | 0 | 0.00 | 4.87 | 0.00 | 6.86 | 0.00 | 5.11 | 0.00 |
| 1,198 | PC-5p-49666_7 | AGACTAATCGAACCATCT | New | 0 | 0.00 | 1.62 | 0.00 | 3.92 | 0.00 | 2.56 | 0.00 |
| 1,199 | PC-3p-73188_4 | AATGAACTTTTTGGCCAGCCCT | New | 0 | 0.00 | 3.25 | 0.00 | 3.92 | 0.00 | 0 | 0.00 |
| 1,200 | PC-3p-28930_14 | GTTCGGACATATATTTTT | New | 0 | 0.00 | 12.19 | 0.00 | 0.98 | 0.00 | 0 | 0.00 |
| 1,201 | PC-5p-80192_4 | TACACCTGGGACCAAGAGACAGT | New | 0 | 0.00 | 0.81 | 0.00 | 0 | 0.00 | 7.67 | 0.00 |
| 1,202 | PC-3p-42231_9 | TGTCCCTGGCCCTCTCCCCAGT | New | 0 | 0.00 | 1.62 | 0.00 | 6.86 | 0.00 | 0 | 0.00 |
| 1,203 | PC-3p-107076_3 | ATACCCTGAATGAACTTTTTGG | New | 0 | 0.00 | 4.06 | 0.00 | 0 | 0.00 | 0 | 0.00 |
| 1,204 | PC-3p-52034_6 | AAAACCTGAATGAACTTTTTGTT | New | 0 | 0.00 | 3.25 | 0.00 | 0.98 | 0.00 | 1.28 | 0.00 |
| 1,205 | PC-5p-85210_3 | ACTGTACTTGCAGCACTG | New | 0 | 0.00 | 0 | 0.00 | 0 | 0.00 | 3.84 | 0.00 |
| 1,206 | PC-3p-68603_5 | TCGAGGGGACGCCGCCGTCCTC | New | 0 | 0.00 | 3.25 | 0.00 | 0.98 | 0.00 | 1.28 | 0.00 |
| 1,207 | PC-3p-128483_2 | TCTGACCACCTGTCTCTCCGCA | New | 0 | 0.00 | 2.44 | 0.00 | 0.98 | 0.00 | 3.84 | 0.00 |
| 1,208 | PC-5p-106697_3 | TTGGCCAAGAATTTCATTTGGATT | New | 0 | 0.00 | 4.06 | 0.00 | 1.96 | 0.00 | 1.28 | 0.00 |
| 1,209 | PC-3p-31500_13 | TAAGAGGACAGTGTGTGGT | New | 0 | 0.00 | 4.06 | 0.00 | 9.80 | 0.00 | 0 | 0.00 |
| 1,210 | PC-5p-37844_10 | AGTTGCAGCTGATTTTCC | New | 0 | 0.00 | 3.25 | 0.00 | 3.92 | 0.00 | 5.11 | 0.00 |
| 1,211 | PC-5p-170446_2 | CGAGCTGGAGGATTCTGCGTT | New | 0 | 0.00 | 1.62 | 0.00 | 5.88 | 0.00 | 3.84 | 0.00 |
| 1,212 | PC-3p-61575_5 | AAACTGAATGAACATTTTGGCC | New | 0 | 0.00 | 0 | 0.00 | 0.98 | 0.00 | 5.11 | 0.00 |
| 1,213 | PC-5p-52312_6 | TGGGAATGATGATTTCTC | New | 0 | 0.00 | 0 | 0.00 | 4.90 | 0.00 | 1.28 | 0.00 |
| 1,214 | PC-3p-112126_3 | ATAGGTAGTAGGTTGTATAG | New | 0 | 0.00 | 4.06 | 0.00 | 0 | 0.00 | 0 | 0.00 |
| 1,215 | PC-3p-113897_2 | AAACCCCGAATGAACTTTTTGT | New | 0 | 0.00 | 4.06 | 0.00 | 0 | 0.00 | 0 | 0.00 |
| total |  |  |  | 6500189.44 | 1.00 | 7487817.98 | 1.00 | 7166600.98 | 1.00 | 7518015.62 | 1.00 |

Note: 1. New, miRNA identified in this study and not reported in miRBase,mainly for new reported 5p or 3p sequence; 2. Diff, confirming miRNA sequences in miRBase, but different sequences are reported in our study; 3. Yes, confirming miRNA sequences in miRBase. 4. Norm: Normalized data. 5. High: The number of reads in the following reported miRNAs is higher than the average copy of the data set. 6. Medium: The number of reads in the following reported miRNAs is higher than 10 and less than average copy of the data set. 7. Low: The number of reads in the following reported miRNAs is less than 10.
